# Gene expression atlas of energy balance brain regions

**DOI:** 10.1172/jci.insight.149137

**Published:** 2021-08-23

**Authors:** Maria Caterina De Rosa, Hannah J. Glover, George Stratigopoulos, Charles A. LeDuc, Qi Su, Yufeng Shen, Mark W. Sleeman, Wendy K. Chung, Rudolph L. Leibel, Judith Y. Altarejos, Claudia A. Doege

**Affiliations:** 1 Department of Pediatrics and Molecular Genetics,; 2 Naomi Berrie Diabetes Center, College of Physicians and Surgeons,; 3 Columbia Stem Cell Initiative, and; 4 New York Obesity Nutrition Research Center, Department of Medicine, Columbia University Irving Medical Center, New York, New York, USA.; 5 Regeneron Pharmaceuticals Inc., Tarrytown, New York, USA.; 6 Department of Systems Biology,; 7 Department of Biomedical Informatics,; 8 Department of Medicine,; 9 Herbert Irving Comprehensive Cancer Center,; 10 Institute of Human Nutrition,; 11 Department of Pathology and Cell Biology, Columbia University Irving Medical Center, New York, New York, USA.

**Keywords:** Genetics, Metabolism, Molecular genetics, Obesity

## Abstract

Energy balance is controlled by interconnected brain regions in the hypothalamus, brainstem, cortex, and limbic system. Gene expression signatures of these regions can help elucidate the pathophysiology underlying obesity. RNA sequencing was conducted on P56 C57BL/6NTac male mice and E14.5 C57BL/6NTac embryo punch biopsies in 16 obesity-relevant brain regions. The expression of 190 known obesity-associated genes (monogenic, rare, and low-frequency coding variants; GWAS; syndromic) was analyzed in each anatomical region. Genes associated with these genetic categories of obesity had localized expression patterns across brain regions. Known monogenic obesity causal genes were highly enriched in the arcuate nucleus of the hypothalamus and developing hypothalamus. The obesity-associated genes clustered into distinct “modules” of similar expression profile, and these were distinct from expression modules formed by similar analysis with genes known to be associated with other disease phenotypes (type 1 and type 2 diabetes, autism, breast cancer) in the same energy balance–relevant brain regions.

## Graphical abstract

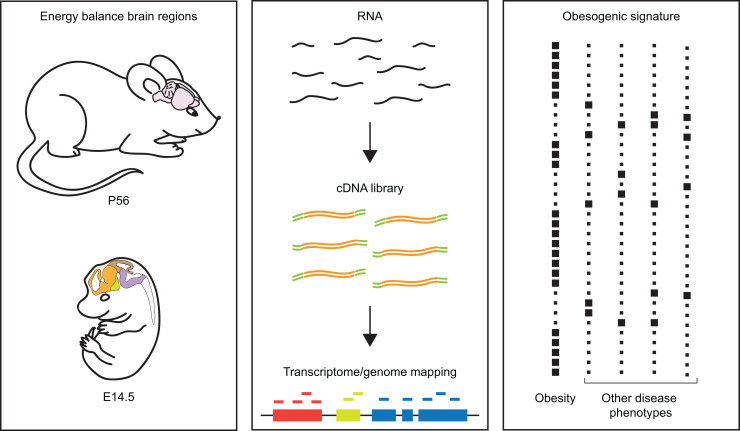

## Introduction

**Figure 1:**
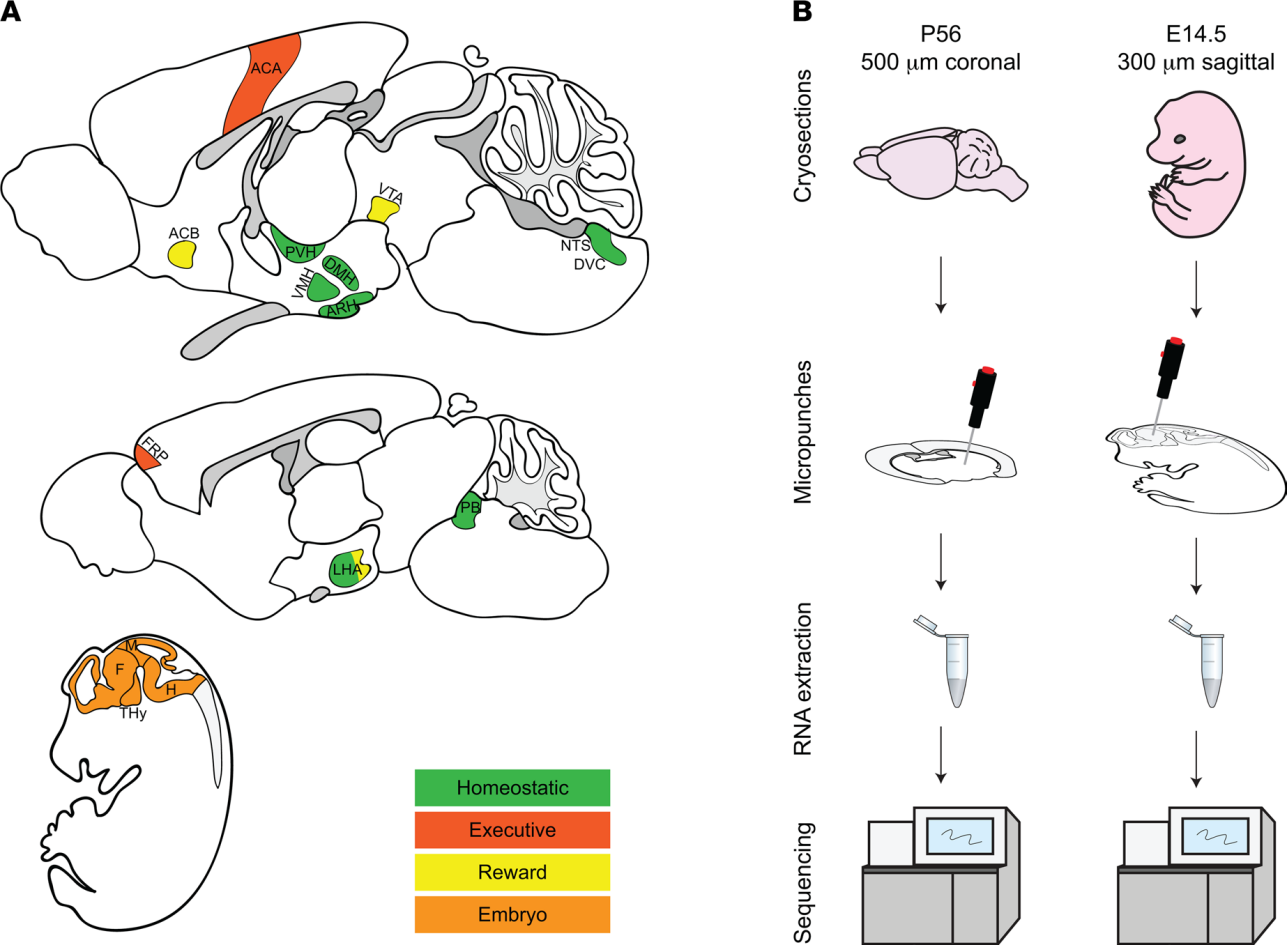
Bulk RNA-Seq of brain regions involved in the regulation of energy balance. (**A**) Regions of the hypothalamus and brainstem (green) include the paraventricular hypothalamic nucleus (PVH), the dorsomedial nucleus of the hypothalamus (DMH), the ventromedial hypothalamic nucleus (VMH), the arcuate hypothalamic nucleus (ARH), the nucleus of the solitary tract (NTS), the dorsal-vagal complex (DVC), the lateral hypothalamic area (LHA), and the parabrachial nucleus (PB). Regions of the brain executive system (red) include the anterior cingulate area (ACA) and the frontal cortex (FRP). Regions of the brain reward system (yellow) include the nucleus accumbens (ACB), ventral tegmental area (VTA) and LHA. The regions in the developing brain include forebrain (F), terminal hypothalamus (THy) and peduncular hypothalamus (PHy), midbrain (M), and hindbrain (H). (**B**) Schematic of workflow from cryosections to micropunches and RNA extraction and sequencing for the adult (left-hand side of panel) and embryonic (right-hand side of panel) mouse brain.

Energy balance is controlled by the intricate interplay of gene expression in the hypothalamus, brainstem, cortex, and limbic system (Figure 1A). The hypothalamus and brainstem are part of the homeostatic circuitry involved in sensing and controlling the energy status of the organism by integrating multiple peripheral metabolic inputs — homeostatic signals — such as circulating metabolites, gut-derived hormones, and adiposity-related signals (1, 2). Cortical and limbic brain regions form the executive and reward systems of the forebrain corticolimbic appetitive network. The executive system is involved in the conscious and voluntary decision to eat (3, 4). Regions of the reward system establish the motivational (incentive salience) and pleasurable (hedonic) values of energy balance–associated stimuli and behaviors (5). Homeostatic, reward, and executive regions are interconnected by extensive neuronal circuits (5). Disturbances in any of these regions or their interconnecting neurocircuitry can lead to an imbalance of food intake and energy expenditure resulting in obesity. To understand the pathogenetic mechanisms of obesity, detailed knowledge about qualitative and quantitative gene expression patterns of these brain regions is essential.

Monogenic obesities confirm the essential roles of specific genes in body weight homeostasis in mice and humans (6–8). The vast majority of human obesity is not monogenic. Many genes of small effect account for only approximately 10% of the apparent approximately 40% risk variance for obesity within a specific environment (9–11). Efforts to find the missing inheritance — in less prevalent genetic variants of novel genes — have led to the extensive use of whole-exome sequencing (WES) in pedigrees or association analyses of extremely obese individuals (12). The vetting of novel variants for functional relevance can be conducted in cell-based and animal transgenic systems (13, 14). To assist in prioritizing genes/alleles for such resource-intensive strategies, brain regional expression patterns can be used.

Although there are several comprehensive public resources (e.g., GTExPortal, Brain Architecture Project) reporting large-scale gene expression data from many tissues, none of them allows the direct comparison of the molecular signature based on bulk RNA sequencing (bulk RNA-Seq) profiling of specific brain regions involved in the regulation of energy balance. Therefore, we performed bulk RNA-Seq of obesity-relevant brain regions comprising the nominal homeostatic, reward, and executive regions in both adult and embryonic mouse brains (Figure 1, A and B; and Supplemental Table 1; supplemental material available online with this article; https://doi.org/10.1172/jci.insight.149137DS1). Additional sequencing was obtained from brain regions considered not to be involved in the regulation of energy balance, as well as mouse embryonic stem cells (Supplemental Figure 1, A–C; Figure 1B; and Supplemental Table 1). Our study also aims to characterize the utility of this database for vetting obesity-associated genes of interest. Thus, we mapped known obesity-associated genes — including monogenic obesity genes, rare coding variants, low-frequency coding variants, syndromic obesity genes, and variants identified in GWAS for BMI — onto the expression profiles of the brain regions with functional roles in energy balance. Moreover, we identified specific expression patterns for obesity-associated genes in obesity-relevant brain regions compared with genes associated with other disease phenotypes — type 1 diabetes (T1D), type 2 diabetes (T2D), autism, and breast cancer. Furthermore, to facilitate usage of these data, we provide access via a publicly available web portal, the Brain Energy Balance Atlas, accessible via http://doegelab.com, permitting the region-specific analyses of any gene of interest.

## Results

### Samples cluster according to brain area specification and developmental stage.

**Figure 2:**
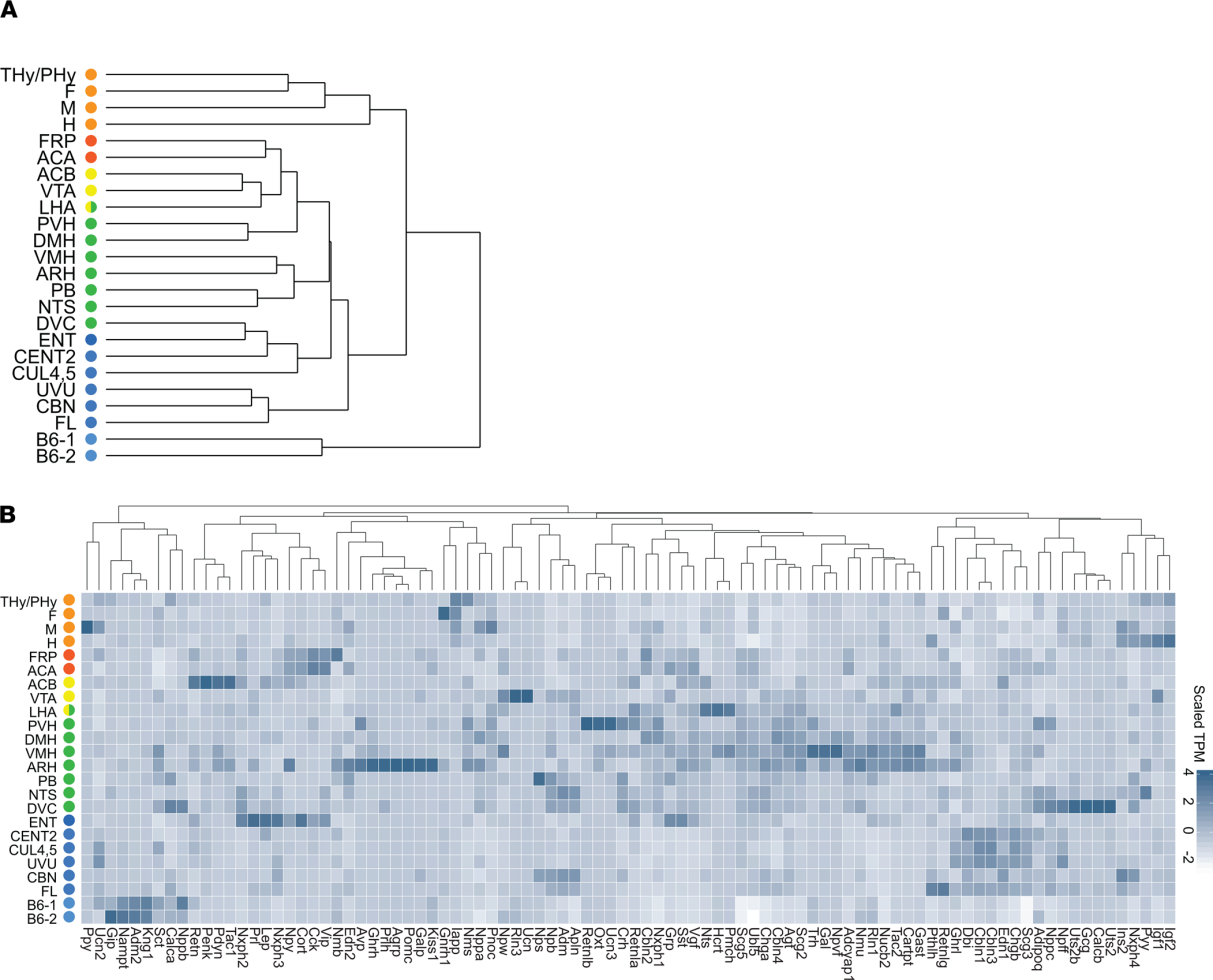
Brain regions cluster according to brain area specification and developmental stage and express known neuropeptides. (**A**) Dendrogram (unsupervised hierarchical clustering) showing similarity of samples according to the anatomical location within the brain with clear distinction of the embryonic samples and the mESCs. (**B**) Heatmap of known neuropeptides. Genes and brain regions were sorted by hierarchical clustering of scaled genes. Scaled TPM values are given in shades of blue. B6-1/B6-2, mouse embryonic stem cell; TPM, transcripts per million.

RNA-Seq was performed on 57 samples from brain regions (regions of energy balance and additional regions) from P56 C57BL/6NTac male mice (Figure 1, A and B, Supplemental Figure 1, and Supplemental Figure 2); 9 samples from 4 brain regions from E14.5 C57BL/6NTac mouse embryos (Figure 1, A and B, and Supplemental Figure 3); and 4 samples from 2 mouse embryonic stem cell (mESC) lines derived from C57BL/6 mice (Supplemental Figure 1); sample description including replicates is given in Supplemental Table 2 and Supplemental Table 3. We performed hierarchical clustering of brain regions and mESC samples using expression profiles of all protein-coding genes. As expected, samples clustered according to brain area specification and developmental stage, with a clear separation of mESC samples (Figure 2A).

We analyzed the distribution of known neuropeptides (*n* = 92; ref. 15). Well-characterized neuropeptides such as pro-opiomelanocortin (*Pomc*), agouti-related protein (*Agrp*), neuropeptide Y, cart prepropeptide (*Cartpt*), and kisspeptin were specifically expressed in the arcuate hypothalamic nucleus (ARH); oxytocin (*Oxt*), corticotropin-releasing hormone (*Crh*), thyrotropin-releasing hormone (*Trh*) in the paraventricular hypothalamic nucleus (PVH; Figure 2B); and hypocretin neuropeptide precursor (*Hcrt*) in the lateral hypothalamic area (LHA; Figure 2B). These findings suggest that the punch biopsies were highly specific for the region of interest (ROI).

### Genetic categories of obesity have specific frequency distributions and enrichment scores across brain regions mediating energy balance.

We mapped known obesity-associated genes to our data set. The obesity-associated genes included in this study belong to 5 genetic categories: (a) monogenic obesity (*n* = 9), caused by single gene defect (e.g., *Lepr*; ref. 16); (b) rare coding variants that have been associated with increased BMI (*n* = 12) with a human minor allele frequency (MAF) of less than 1% (e.g., *Plxna3*; refs. 14, 17, 18); (c) low-frequency coding variants associated with BMI (*n* = 9), MAF = 1% to 5% (e.g., *Ache*; ref. 18); (d) syndromic obesity (*n* = 25), which are genes that cause a known syndrome that has obesity as 1 phenotypic manifestation (e.g., *Phip*; refs. 19–23); (e) genes inside loci identified by GWAS of BMI (*n* = 144) (e.g., *Tcf7l2*; refs. 24–30; and Supplemental Table 4 and Supplemental Table 5). For genes identified in more than 1 modality (*Pomc*, *Mc4r*, *Lepr*, *Bdnf*, *Tub*, *Ntrk2*, *Bbs4*, *Sh2b1*, *Gipr*), the genes were counted in all relevant categories.

**Figure 3:**
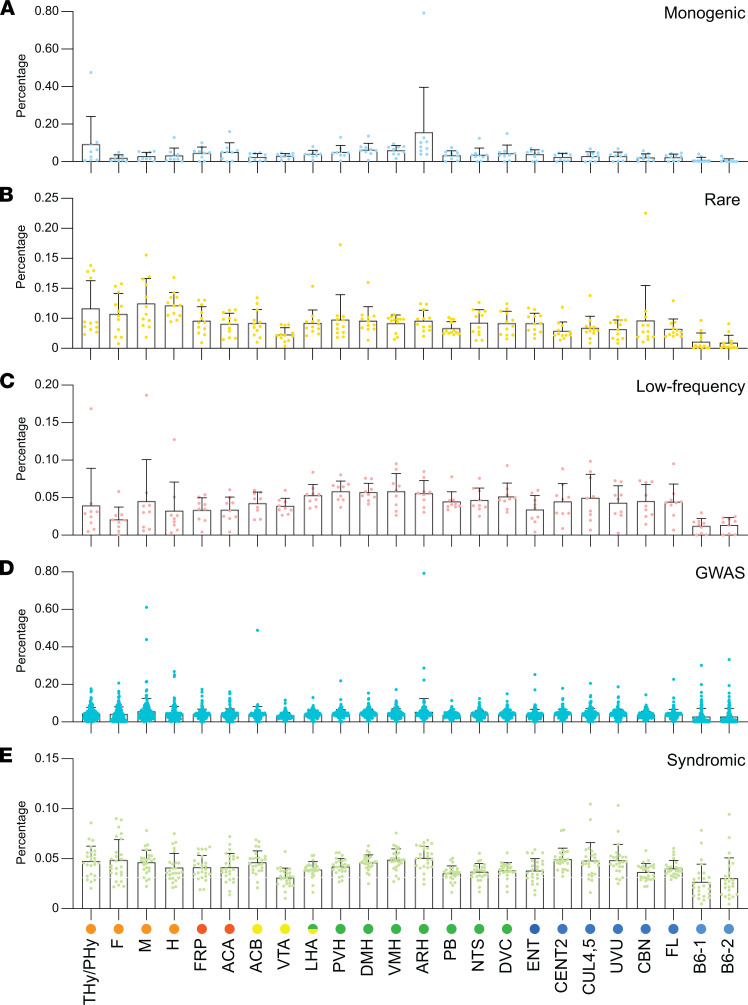
Frequency distribution of obesity-associated genes across the brain regions of energy balance for each genetic category. The frequency distribution of each obesity-associated gene was determined by calculating the TPM percentage for each region compared with the sum of TPM values for all samples (brain regions and mESCs, averaged across each replicate). For each genetic category, the frequency score was calculated by compiling the percentage for all the genes in a gene list, shown as mean ± SEM. Individual percentage values for each gene were displayed as dots. (**A**) Monogenic, *n* = 9; (**B**) rare, *n* = 12; (**C**) low-frequency, *n* = 9; (**D**) GWAS, *n* = 144; (**E**) syndromic, *n* = 25. B6-1/B6-2, mouse embryonic stem cell; TPM, transcripts per million.

All obesity-associated genes were detected in the brain at variable levels in at least 1 region per gene (Supplemental Figure 4 and Supplemental Table 6). To assess whether these obesity-associated genes are enriched in any of the brain regions involved in energy balance, the frequency distribution was calculated for each obesity-associated gene, by calculating the percentage a sample contributes to the total transcripts per million (TPM) for each gene. For each genetic category, the data were compiled, and the average was plotted (Figure 3, A–E). This was repeated for all categories together (Supplemental Figure 5A).

Monogenic obesity genes were significantly enriched in the ARH compared with any other brain region; monogenic obesity genes also showed enrichment in the terminal hypothalamus (THy) and peduncular hypothalamus (PHy). Statistical comparisons between brain regions are given in Supplemental Table 7. The other genetic categories showed more equal distribution across brain regions. There were trends for relative enrichments in some regions, such as among genes associated with rare coding variants in embryonic brain, low-frequency coding variants in hypothalamic regions, GWAS genes in ARH and embryonic midbrain, and syndromic genes in hypothalamus and cerebellum (Figure 3 and Supplemental Table 7). For all obesity-associated genes as a group (*n* = 190), we observed a trend for enrichment in some regions, such as ARH and embryonic midbrain (Supplemental Figure 5A and Supplemental Table 8).

**Figure 4:**
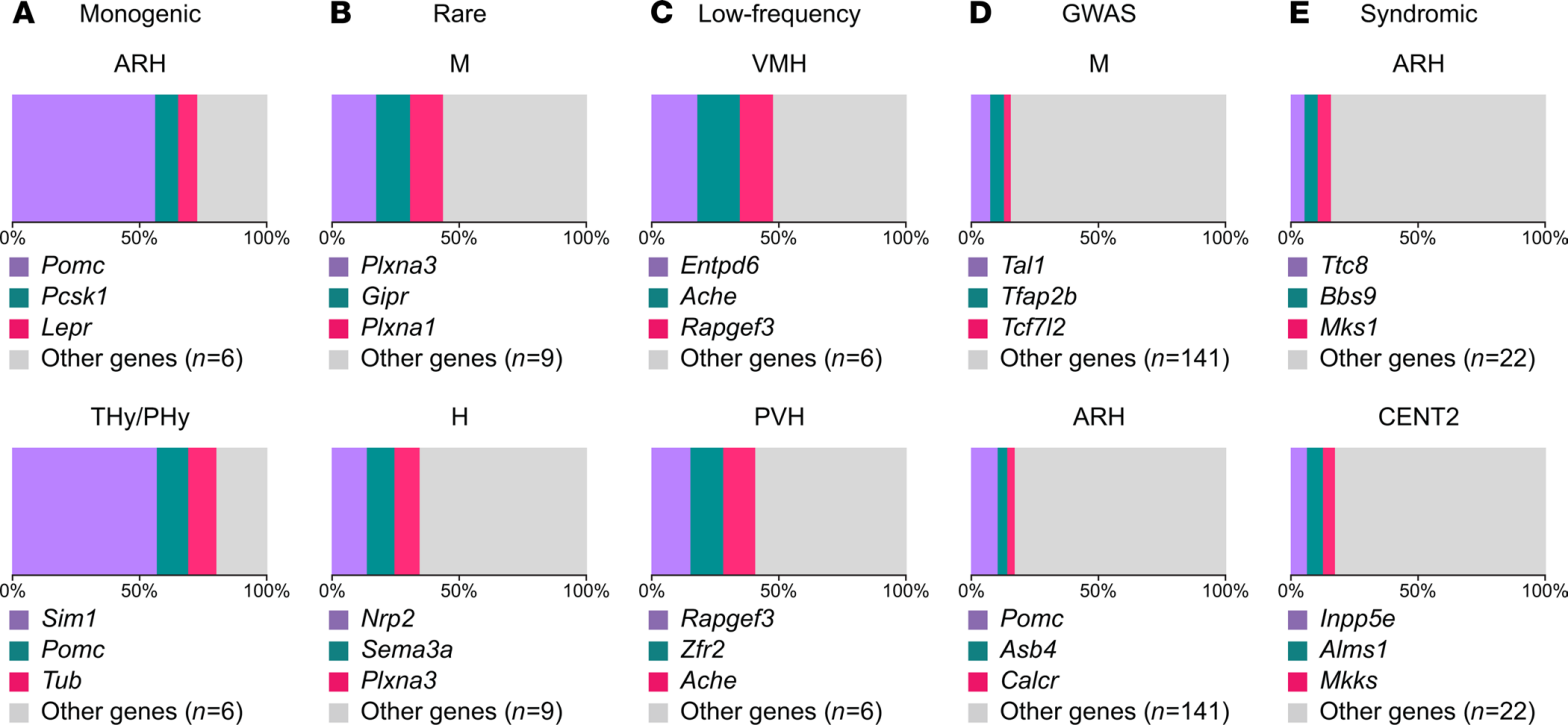
Detailed analysis of the 2 most enriched brain regions for each form of obesity inheritance. Visualization of the 3 genes with the highest percentage expression in the 2 most enriched regions for (**A**) monogenic genes; (**B**) rare coding variants; (**C**) low-frequency coding variants; (**D**) GWAS loci; (**E**) syndromic genes. Each slice represents the percentage of expression for a specific gene or group of genes (as indicated in the legend), and the sum of all the slices represent the total expression (100%) of genes for a specific genetic category in a selected brain region.

To better understand the genetics underlying these frequency distributions, we identified the 3 genes with the highest percentage expression among the obesity-associated genes in the 2 most enriched regions, within each genetic category (Figure 4) as well as within all the obesity-associated genes (Supplemental Figure 5B). The genes in the top 2 enriched regions for monogenic obesity were *Pomc*, *Pcsk1*, and *Lepr* in ARH, and *Sim1*, *Pomc*, and *Tub* in THy/PHy. All of these genes are expected to be present in these regions and the majority of them are components of the leptin–melanocortin pathway. These results are consistent with prior studies that have classified monogenic obesity mutations into genes with roles in the hypothalamic melanocortin system of feeding regulation (e.g., *Lepr*, *Pomc*) and genes that are essential for the development of the hypothalamus (e.g., *Sim1*; ref. 16). The embryonic midbrain with *Plxna3*, *Gpr*, and *Plxna1* and embryonic hindbrain with *Nrp2*, *Sema3a*, and *Plxna3* are the 2 regions (and top genes) identified as the most enriched for rare coding variants. Among genes associated with low-frequency coding variants, we identified *Entpd6*, *Ache*, and *Rapgef3* as genes with the highest TPM values in the ventromedial hypothalamic nucleus (VMH) and *Rapgef3*, *Zfr2*, and *Ache* in the PVH. Among syndromic genes, we identified *Ttc8*, *Bbs9*, and *Mks1* in ARH, and *Inpp5e*, *Alms1*, and *Mkks* in central lobule II (CENT2; Figure 4). The genes in the top 2 enriched regions for GWAS loci were *Tal1*, *Tfap2b*, and *Tcf7l2* in embryonic hindbrain and *Pomc*, *Asb4*, and *Calcr* in the ARH (Figure 4). These regions and genes were also identified, when combining all genes of all genetic categories (Supplemental Figure 5B).

In conclusion, we observed that among all the analyzed genetic categories associated with obesity, monogenic obesity genes were most enriched in arcuate nucleus and embryonic hypothalamus. We also observed that for all obesity-associated genes, there was a trend for enrichment in ARH and embryonic brain regions.

Next, we performed gene set enrichment analysis (GSEA; Supplemental Figure 6, A–F). All replicates were input, alongside the obesity genetic category gene lists (monogenic, rare coding variance, low-frequency coding variants, GWAS, and syndromic). Results of GSEA are as follows: monogenic obesity genes were significantly enriched in the arcuate nucleus of the hypothalamus and showed a trend of enrichment in the embryonic hypothalamus (Supplemental Figure 6, A and B); rare coding variants were significantly enriched in embryonic hypothalamus and hindbrain (Supplemental Figure 6, A and C); low-frequency coding variants were enriched in hypothalamic regions, significantly in the dorsomedial nucleus of the hypothalamus (DMH; Supplemental Figure 6, A and D); GWAS genes were enriched in the embryonic midbrain, among hypothalamic regions significantly in the ARH and DMH, and in regions of the cerebellum (Supplemental Figure 6, A and E); and syndromic genes were enriched in regions of hypothalamus and cerebellum, significantly in the ARH, VMH, and CENT2 (Supplemental Figure 6, A and F). In conclusion, as shown above for the frequency distribution, GSEA determined that obesity-associated genes are enriched in the ARH and embryonic hypothalamus. In addition, GSEA also identified the enrichment of obesity-associated genes in the VMH, DMH, and regions of the cerebellum.

### Obesity-associated genes cluster into modules.

**Figure 5:**
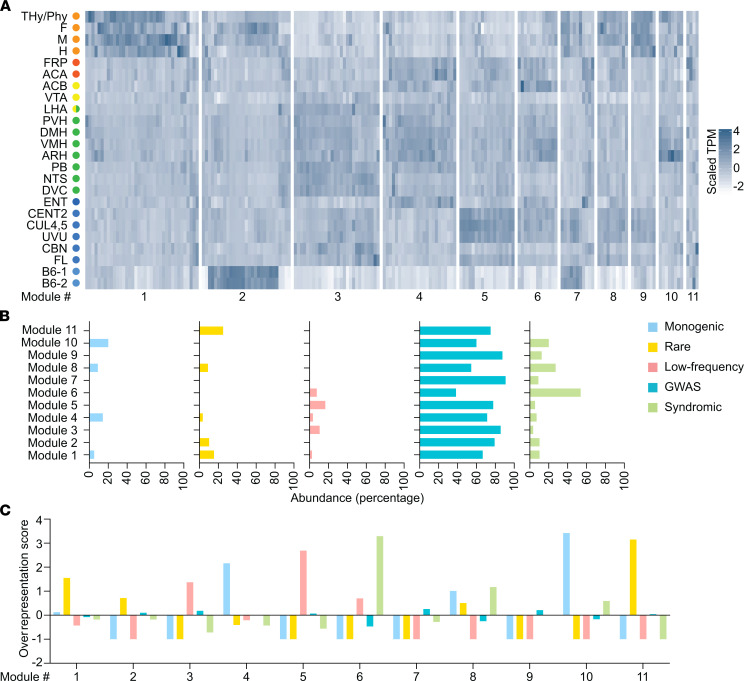
Organization of obesity-associated genes into modules using WGCNA. (**A**) Heatmap of obesity-associated genes. TPM values were scaled for each gene to improve visualization. Higher expression is denoted by darker shades of blue. The order of genes on the heatmap was determined by the module number, followed by the order the gene appears in hierarchical clustering. (**B**) Relative abundance (given as percentage) of the genes within the modules, based on the genetic categories. (**C**) Overrepresentation score of the genes within the modules, based on the genetic categories, showing a positive deflection where a gene list is overrepresented. WGCNA, weighted correlation network analysis.

To assess whether obesity genes from different genetic categories show similar or different expression profiles across the brain regions of energy balance, we subjected all obesity-associated genes to weighted correlation network analysis (WGCNA), obtaining 11 modules (Supplemental Figure 7). The heatmap in Figure 5A visualizes the modules, and detailed information (e.g., genes annotation) is given in Supplemental Table 9. In module 1, we observed enrichment of genes in embryonic regions. Genes in module 2 had a higher expression in embryonic regions and stem cells. Module 3 featured genes with a high expression in regions of homeostatic system and cerebellum. Module 4 was characterized by genes with a high expression in regions of the executive system and hypothalamic regions. In module 5, we detected enrichment of genes in regions of the cerebellum. In module 6, the expression was highest in regions of the executive system, reward system, hypothalamus, and cerebellum. Genes defining module 7 had a high expression in regions of the embryonic brain, cerebellum, and in stem cells. In modules 8 and 9, we observed an increased expression of genes in regions of embryonic brain and cerebellum; in differentiating 8 from 9, module 8 had an increased expression in executive regions. Module 10 showed the presence of genes specifically enriched in embryonic and hypothalamic regions. In module 11, genes were more widespread among the regions, with no specific localization (Figure 5A).

To address whether there is an association between expression modules and the genetic categories of obesity evidence, for each module, the number of genes associated to each genetic category was normalized to the size of the module, and the normalized gene number was expressed as a percentage (Figure 5B). All modules are characterized by the presence of genes belonging to more than 1 genetic category, with modules 1 and 4 being defined by genes associated with all 5 genetic categories. Except for GWAS genes that were distributed across all modules, genes from the remaining genetic categories were present only in a subset of the modules. Genes associated with monogenic obesity were primarily expressed in module 10, but also in modules 1, 4, and 8 (Figure 5B), the modules with predominance of genes in embryonic brain and hypothalamus (Figure 5A). These results are in agreement with our previous observations of enrichment of monogenic obesity genes in hypothalamic (ARH) and embryonic (THy/PHy) regions (Figure 3, Figure 4, and Supplemental Figure 6, A and B). Rare coding variants were distributed across modules 1, 2, 4, 8, and 11. Low-frequency coding variant genes were identified in modules 1, 3, 4, 5, and 6, and in particular, in module 5 (Figure 5B), in which there was an abundance of genes in cerebellar regions (Figure 5A). Syndromic genes were distributed across all modules, except module 11, with predominance in module 6 (Figure 5B), as characterized by genes in regions of the executive system, reward system, hypothalamus, and cerebellum (Figure 5A).

Next, we determined whether a genetic category is overrepresented in a given module. We developed an overrepresentation score that indicates the deviation from an equal distribution across modules, normalizing for module size and gene list size. The deviation from the equal distribution was plotted, where a positive deflection denotes enrichment (Figure 5C). Each cluster was enriched with genes specifically associated with 1 of the genetic categories. Genes associated with monogenic obesity were enriched in module 10 (Figure 5C), comprised of genes with a high expression in the embryonic and hypothalamic regions (Figure 5B), again supporting the importance of this genetic category in these regions (Figures 3 and 4 and Supplemental Figure 6, A and B); and module 4 (Figure 5C), with a predominance of genes in the executive and hypothalamic regions (Figure 5B). Rare coding variants were enriched in module 1 (Figure 5C), suggesting the involvement of these genes in embryonic brain (Figure 5B, Figure 3, and Supplemental Figure 6, A and C). Futhermore, rare coding variants were also enriched in modules 2 and 11 (Figure 5C). Low-frequency coding variants were enriched in module 3 and 5 (Figure 5C). These modules feature high gene expression in homeostatic and cerebellar regions (Figure 5A), which is in agreement with the previously identified association of these genetic category to hypothalamic regions (Figure 3 and Supplemental Figure 6, A and D). GWAS genes were enriched in module 7 and 9 (Figure 5C), showing again a predominance of this category in embryonic regions (Figure 5B, Figure 3, and Supplemental Figure 6, A and E). Syndromic genes were enriched in module 6 and 8 (Figure 5C), confirming the presence of these genes in regions of the cerebellum and hypothalamus (Figure 5B, Figure 3, and Supplemental Figure 6, A and F). Taken together, these results show that different genetic evidence for association with obesity can result in similar expression profiles across brain regions.

To further strengthen our findings, we utilized an independent approach to assess the expression profile of obesity-associated genes across brain regions of energy balance. All obesity-associated genes were subjected to hierarchical clustering, resulting in 11 distinct modules, each defined by more than 1 genetic category (Supplemental Figure 8 and Supplemental Table 5). This analysis also found the abundance of genes associated with monogenic obesity in embryonic and hypothalamic regions; the enrichment of rare coding variants in embryonic regions; and the prevalence of low-frequency coding variant genes in regions of the executive system, reward system, and hypothalamus (Supplemental Figure 8). Clustering into additional smaller modules did not lead to the assignment of just 1 genetic category per module (Supplemental Figure 9).

We then looked at changes in the composition of the modules, comparing the 11 modules obtained using WGCNA clustering with the 11 modules obtained performing hierarchical clustering, and observed that the majority of obesity-associated genes had the tendency to cluster in similar ways in the 2 distinct analyses (Supplemental Figure 10), strengthening the validity of both approaches and the biological relevance of our data set.

To confirm that a genetic category is not assignable to a distinct module, we performed hierarchical clustering on all the genetic categories individually and found that genes associated with each of the categories were distributed across all anatomic regions, without distinct preference for homeostatic, executive, or reward system (Supplemental Figure 4, A–E). Thus, the genetic category per se did not determine regional gene expression profile.

### Obesity-associated genes have specific enrichment scores across energy balance–relevant brain regions.

To determine whether the expression patterns of the obesity-associated genes are specific to obesity, we examined the expression profiles of genes known to be associated with 4 other diseases: (a) autoimmune disease (T1D, *n* = 61; ref. 31); (b) metabolic disease (T2D, *n* = 140; refs. 32–36); (c) complex brain disorder (autism, *n* = 190; ref. 37); and (d) cancer (breast cancer, *n* = 98; refs. 38–40; Supplemental Table 10, Supplemental Table 11, Supplemental Table 12, and Supplemental Table 13). The expression values for genes associated with obesity, T1D, T2D, autism, and breast cancer are given in Supplemental Table 14. Transcripts from all genes were detected in the brain at variable levels in at least 1 region per gene. These diseases have some genes in common with obesity (15 genes of 650 total genes; Supplemental Table 15), which may have been suggestive of the presence of shared pathways between diseases and/or obesity as a risk factor for the other diseases and/or vice versa.

**Figure 6:**
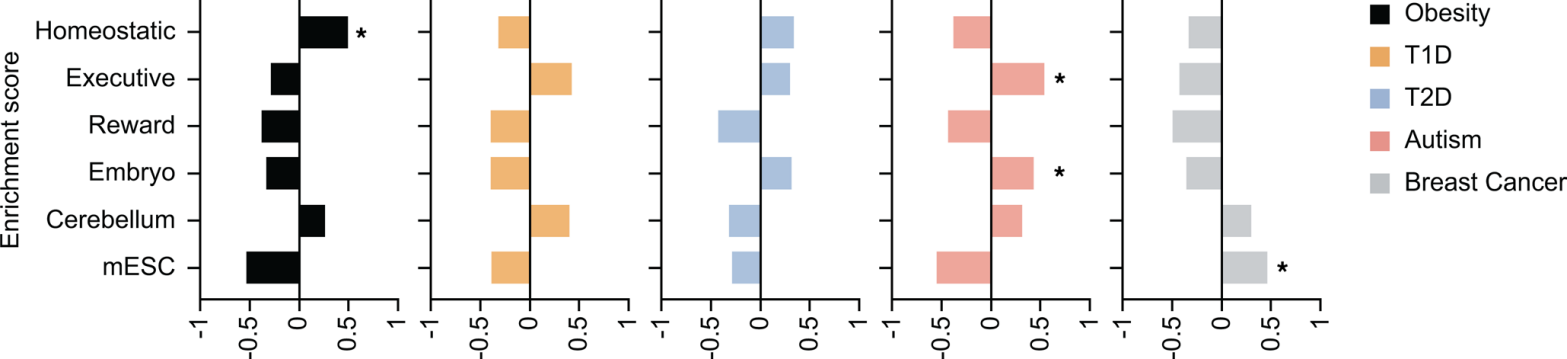
GSEA of obesity-, T1D-, T2D-, autism-, and breast cancer–associated genes across the brain regions of energy balance. For each system (homeostatic, executive, reward, embryonic brain, cerebellum, mESC), samples from all the regions were aggregated. GSEA was run on the 5 disease gene lists. The enrichment score was plotted, and regions with a positive enrichment score and FDR-adjusted nominal *P* < 0.05 (based on GSEA output) were denoted with an asterisk (ref. 139). GSEA, gene set enrichment analysis; T1D, type 1 diabetes; T2D, type 2 diabetes; mESC, mouse embryonic stem cell.

To assess if there was region-specific enrichment for 1 or the other disease, we performed GSEA, where all replicates were input alongside the gene list comprised of all obesity-, T1D-, T2D-, autism- and breast cancer–associated genes (Figure 6 and Supplemental Figure 11, A–E). When analyzed with GSEA, obesity-associated genes were significantly enriched in regions of the homeostatic system (Figure 6 and Supplemental Figure 11A). Autism-associated genes were significantly enriched in regions of the executive system and embryonic brain (Figure 6 and Supplemental Figure 11D). Breast cancer–associated genes were significantly enriched in mESC samples (Figure 6 and Supplemental Figure 11E). T1D- and T2D-associated genes were not significantly enriched in any region but showed a trend of enrichment in some of the regions: T1D-associated genes in regions of the executive system and cerebellum and T2D-associated genes in regions of the homeostatic and executive system and embryonic brain (Figure 6 and Supplemental Figure 11, B and C).

These results show that obesity-associated genes have specific enrichment across brain regions mediating energy balance.

### Clustering of obesity-associated genes together with genes associated with other diseases.

**Figure 7:**
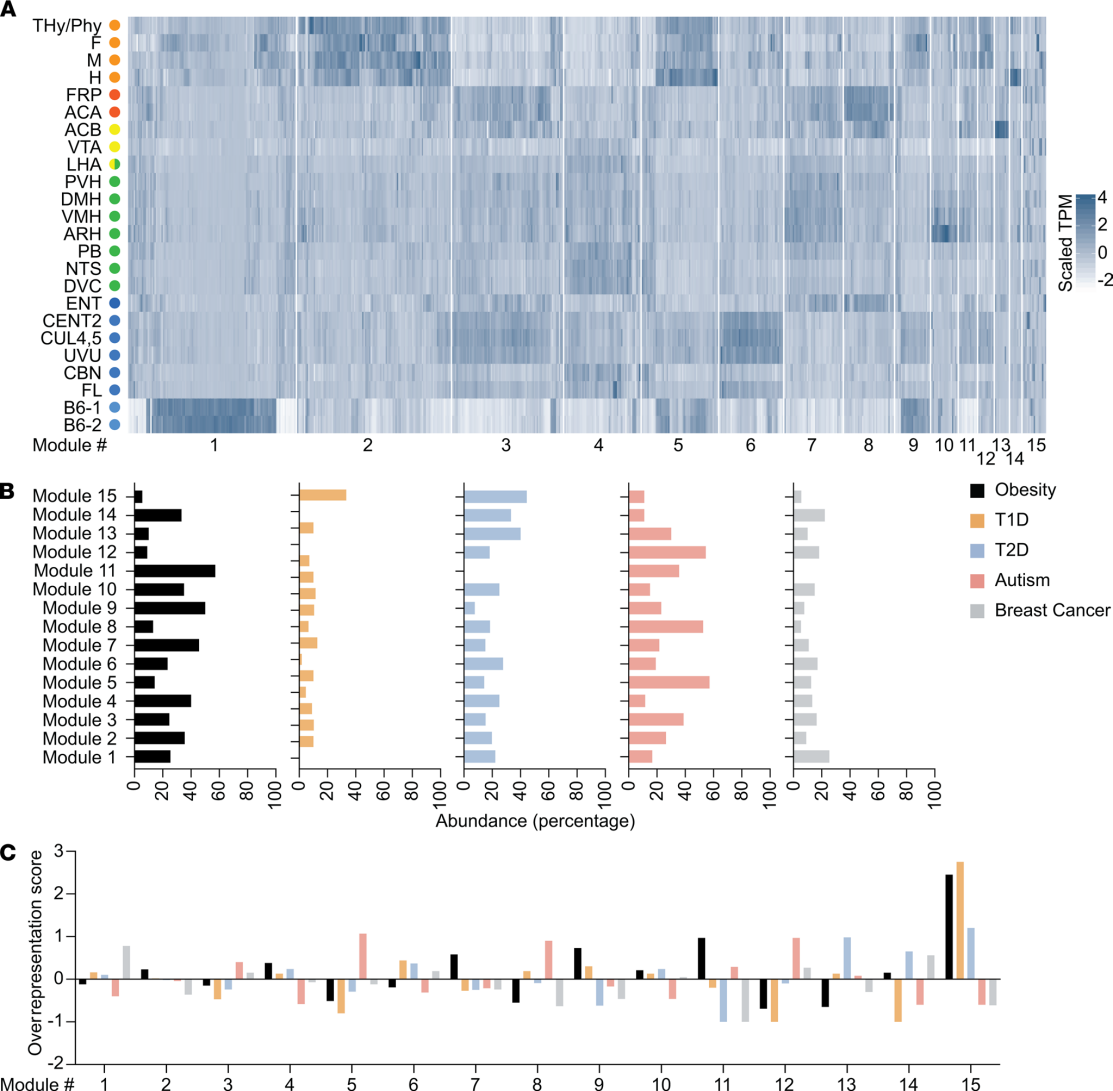
Clustering of obesity-associated genes with genes associated with other diseases using WGCNA. (**A**) Heatmap of obesity-, T1D-, T2D-, autism-, and breast cancer–associated genes. TPM values were scaled for each gene to improve visualization. Higher expression is denoted by darker shades of blue. The order of genes on the heatmap was determined by the module number, followed by the order the gene appears in hierarchical clustering. (**B**) Relative abundance (given as percentage) of the genes within the modules, based on type of disease. (**C**) Overrepresentation score of the genes within the modules, based on the type of disease, showing a positive deflection where a gene list is overrepresented. WGCNA, weighted correlation network analysis; T1D, type 1 diabetes; T2D, type 2 diabetes; TPM, transcripts per million.

To assess if genes associated with the 4 aforementioned diseases cluster with obesity, we compared obesity-associated genes with T1D-, T2D-, autism-, and/or breast cancer–associated genes using WGCNA, resulting in 15 distinct modules (Supplemental Figure 12), each defined by admixed genes associated with all diseases, and each composed of genes with specific regional localization. The heatmap in Figure 7A visualizes the modules, and detailed information (e.g., genes’ annotation) is given in Supplemental Table 16. The percentage of the contribution of each disease to each module, calculated by normalizing the number of genes associated to each disease normalized to the size of the module and expressed as percentage, revealed that all diseases are present, at different levels, in every module. The only exceptions were for T1D-associated genes, absent in modules 1, 12, and 14; and T2D-associated genes, absent in module 11 (Figure 7B).

To evaluate whether the modules were enriched for disease-specific genes, even if composed of genes from all diseases, we generated an overrepresentation score for each gene list by calculating the number of genes that should be in each module if they were equally distributed, normalizing for module size and gene list size. The deviation from the equal distribution was plotted, where a positive deflection denotes overrepresentation (Figure 7C). Module 1 was enriched by breast cancer–associated genes (Figure 7C). This module was characterized by genes with a high expression specifically in stem cells (Figure 7A), confirming the previous GSEA results that show significant enrichment of breast cancer–associated genes in the mESC samples (Figure 6 and Supplemental Figure 11E). Module 2, with a high expression of genes in the embryonic regions (Figure 7A), was enriched by obesity-associated genes (Figure 7C), which was expected to be highly present in these regions. Module 3, characterized by a high expression of genes in regions of the executive system and cerebellum (Figure 7A), is enriched with autism-associated genes (Figure 7C), supporting the significant enrichment previously shown for genes associated with this disease in these regions (Figure 6 and Supplemental Figure 11D). Module 4 was enriched with obesity-associated genes (Figure 7C). This module had a high expression of genes in regions of the homeostatic system and cerebellum (Figure 7A), in agreement with our previous findings (Figure 6 and Supplemental Figure 11A). Autism-associated genes were enriched in module 5 (Figure 7C), characterized by genes highly expressed in embryonic regions and mESCs (Figure 7A). Module 6 was enriched with T1D-associated genes (Figure 7C). This module had an increased expression of genes in cerebellum regions (Figure 7A). We observed a nonsignificant trend of enrichment of T1D-associated genes in the cerebellum in the GSEA analysis (Figure 6 and Supplemental Figure 11A). Obesity-associated genes were enriched in module 7 (Figure 7C). This module showed the highest expression of genes in regions of the executive system and homeostatic system (Figure 7A), confirming the previously obtained results for the latter system (Figure 6 and Supplemental Figure 11D). Module 8, comprised of genes highly expressed in the executive system (Figure 7A), was enriched by autism-associated genes (Figure 7C), confirming our previous findings (Figure 6 and Supplemental Figure 11D). Module 9 was enriched by obesity-associated genes (Figure 7C) and characterized by genes with a high expression in embryonic regions, cerebellum, and mESCs (Figure 7A). Module 10, with a high expression of genes in hypothalamic regions (Figure 7A), was enriched by T2D-associated genes (Figure 7C). Module 11 was enriched by obesity-associated genes (Figure 7C). Module 12 showed enrichment of autism-associated genes (Figure 7C), with genes primarily localized in embryonic regions (Figure 7A). T2D-associated genes were enriched in module 13 (Figure 7C), characterized by a high expression of genes in regions of the reward system (Figure 7A), and module 14 (Figure 7A), defined by genes with a high expression in the embryonic hindbrain (Figure 7A). Taken together, this analysis reveals that clustering of obesity-associated genes together with genes associated with other diseases resulted in modules with a higher contribution from 1 or another disease. Such findings indicate that despite some similarities in the genetic signature between diseases, they clearly showed the presence of different gene expression patterns.

We confirmed these findings with an independent method, k-means clustering. First, we compared obesity with each of the other diseases individually. We combined obesity-associated genes with genes associated with 1 of the other 4 diseases and performed k-means clustering, resulting in 4 distinct clusters, each cluster defined by the genes associated with the 2 diseases (Supplemental Figure 13, A–D, F, G, and I; Supplemental Table 17; Supplemental Table 18; Supplemental Table 19; and Supplemental Table 20). For each combination, the results show that more than half of the clusters had a higher contribution from 1 or the other disease, whereas the remaining clusters had a more equal contribution from both diseases, confirming the presence of unique gene expression patterns, even with the existence of small similarities (Supplemental Figure 13, A–D, F, G, and I; Supplemental Table 17; Supplemental Table 18; Supplemental Table 19; and Supplemental Table 20).

Secondly, we also combined together obesity-, T1D-, T2D-, autism-, and breast cancer–associated genes and performed k-means clustering, resulting in 6 distinct clusters, each defined by admixed genes associated with all diseases (Supplemental Figure 13, E and J; and Supplemental Table 21). Each cluster was enriched with genes specifically associated with 1 of the diseases, and as consequence, there was no cluster with equal; genetic contribution from all diseases (Supplemental Figure 13, E and J, and Supplemental Table 21).

In summary, although there was some overlap in the genetic signature between obesity, T1D, T2D, autism, and breast cancer, there was a large genetic component characterized by a clear separation, suggesting an obesogenic signature of obesity-associated genes in brain regions that function in the regulation of energy balance.

## Discussion

In this study, gene expression profiles of 16 energy balance–relevant brain regions were obtained from P56 mice and E14.5 embryos using bulk RNA-Seq. The brain regions chosen were compiled from a comprehensive literature search of regions known to be involved in the control of energy balance, as reviewed by Caron and Richards (5). To punch the ROIs with the highest possible accuracy, we chose P56 as the age of adult mice to allow the use of landmarks from the mouse brain map of the Allen Brain Atlas with the punch size chosen by the size of the ROI. Canonical neuropeptides involved in the regulation of body weight (e.g., *Pomc*, *Agrp*, *Oxt*) and those involved in other functions that are known to map to distinct regions (e.g., *Cartpt*, *Hcrt*, *Trh*, *Crh*) show the expected region-specific expression pattern. This neuropeptidergic expression pattern supports the accuracy of the regional identifications and sample ascertainment.

As expected, known obesity-associated genes (*n* = 190) were enriched in at least 1 of the brain regions of nominal homeostatic, reward, and executive circuitry. Monogenic obesity genes were expressed in specific regions of the homeostatic circuits as well as the developing hypothalamus. The arcuate nucleus was found to be the region most enriched by the 9 known human monogenic obesity genes, with the highest expression of *Pomc*, *Pcsk1*, and *Lepr* genes, followed by the embryonic hypothalamus, with prevalent expression of *Sim1*, *Pomc*, and *Tub* genes. Our results are in agreement with published literature that strongly associates monogenic forms of obesity, characterized by severe, early-onset obesity, with loss-of-function mutations in genes of the hypothalamic leptin–melanocortin pathway, which plays a critical role in the regulation of food intake and body weight (41), e.g., *POMC* (42–51), *LEPR* (52–69), *PCSK1* (70–77), *SIM1* (78–87), and tubby-like protein *TUB* (88–92). Furthermore, we observed that genes carrying rare coding variants, low-frequency coding variants, and genes identified in GWAS, as well as genes associated with syndromic obesity, were enriched in at least 1 of the energy balance brain regions: rare coding variants in the embryonic hindbrain; low-frequency coding variants and GWAS genes in homeostatic regions; and syndromic genes in hypothalamic regions. Interestingly, our data show specific enrichment for syndromic and GWAS genes in regions of the cerebellum, a region whose link with obesity has not yet been well defined. For these genes, we have to consider the following 2 options: (a) expression in these samples is a measure for additional functions of these genes, independent of their role in obesity, and (b) cerebellum is truly contributing to the regulation of body weight. At this point, experimental testing of the above hypothesis is needed to understand such expression pattern.

WGCNA clustering of known obesity-associated genes revealed that these genes cluster in distinct modules, and each module is defined by more than 1 genetic category (similar results were obtained performing an independent hierarchical clustering analysis). Monogenic obesity genes were predominantly expressed in module 10, characterized by enrichment for genes expressed in the embryonic brain and hypothalamus. Furthermore, the genes defining this module include *Pomc* and *Tub*. Six modules (modules 1, 2, 7, 8, 9, 10) were characterized by enrichment of genes in the embryonic regions, consistent with a role for neurodevelopmental processes mediating susceptibility to obesity (93, 94). In fact, these modules contained genes known to participate in brain development, but not functionally characterized with regard to obesity, such as *Hmgcr* (95), *Klf7* (96), and *Lmo1* (97, 98), as well as genes whose role in both development and obesity have been functionally characterized, such as *Sim1* (78–87), *Creb1* (99, 100), *Nrp2* (14), and *Phip* (22, 23, 101, 102).

In addition, we identified modules with enrichment of obesity-associated genes in mESCs or cerebellum. Modules 2 and 7 are characterized by enrichment of genes in the mESC lines and embryonic brain. Genes in these modules implicated in obesity are *Gdf15* (103), *Alms1* (104), and *Rab23* (20). Their dysfunction during neurodevelopment could contribute to susceptibility to obesity. Modules 3, 5, 6, 8, and 9 are characterized by enrichment of genes in regions of the cerebellum. Some of the genes present in these modules have been functionally associated with obesity, including *Irs1* (105), *Sdccag8* (106, 107), *Negr1* (108–111), *Ksr2* (112, 113), *Tlr4* (114, 115), and *Sh2b1* (116, 117). Mice with either *Lepr* neuron–specific or adult-onset, hypothalamus-specific ablation of *Sh2b1* develop obesity, insulin resistance, and liver steatosis (118, 119). It would be interesting to test whether knocking out *Sh2b1* specifically in the cerebellum the mice would result in the same phenotype. *Cep290* (120, 121) and *Inpp5e* (19), associated with syndromic ciliopathies that include obesity, and Bardet-Biedl syndrome (BBS) causal genes, *Arl6*, *Bbs1*, *Bbs2*, *Bbs5*, *Bbs7* (122), were also present in these modules. Whether genes with an enriched expression in the cerebellum are truly causal of obesity is unclear, because the majority of them also showed some enrichment in other brain regions. Some patients with BBS display characteristic structural brain abnormalities, including within the cerebellum (123, 124). The literature implicating the cerebellum in weight regulation does not identify specific neuronal circuitry or molecular mechanisms for such an effect (125–132). A role in anticipatory aspects of ingestive behaviors — similar to the cerebellum’s classical role in motor activities (133) — is an interesting possibility, the study of which may be assisted by analytic strategies and the tools developed in this project reported here.

To address whether there is an obesogenic signature of obesity-associated genes in the brain regions of energy balance, in our analysis we included genes associated with 4 additional diseases: T1D, an autoimmune disease; T2D, a metabolic disease; autism, a complex neurobehavioral disorder; and breast cancer. These diseases share some gene overlap with obesity (15 genes out of 650 total genes), suggesting the presence of shared pathways between diseases and/or obesity as a risk factor for the other diseases and/or vice versa. We observed an enrichment of obesity-associated genes in regions of the homeostatic system. Genes associated with autism, a neurodevelopmental disorder, were enriched, as expected, in regions of the embryonic brain and the executive system. Breast cancer–associated genes showed enrichment in the stem cells samples. T1D and T2D did not show region-specific enrichment. WGCNA clustering of obesity-associated genes together with T1D-, T2D-, autism-, and breast cancer–associated genes across the regions of energy balance revealed that these genes clustered in distinct modules, and that each module was defined by genes associated to each disease, with few exceptions, and was characterized by the prevalence of 1 disease over the others. Similar findings were observed in k-means clustering of genes associated with these 5 diseases together. Regardless of the comparison examined, some clusters had more equal contribution from the diseases, whereas more than half of the clusters showed predominant contribution from 1 disease over the other. These results suggest that there was a clear separation between the diseases and thus an obesogenic signature of obesity-associated genes in the brain regions of energy balance.

The online database and analytic strategies presented here can be utilized to vet novel obesity candidate genes by “positioning” them within specific gene clusters and neural circuits. This information can be used to determine next steps with regard to functional analyses. More specifically, to fulfill the promise of precision medicine in obesity, we envision the following workflow: clinical genetics using WES/GWAS to identify novel obesity candidate genes; determine brain region/s of enrichment for a given novel obesity candidate gene for prioritization using the Brain Energy Balance Atlas portal (http://doegelab.com); determine the specific cell type/s expressing the candidate gene using single-cell RNA-Seq (scRNA-Seq) in the determined region/s; generation of induced pluripotent stem cells (iPSCs) from patient’s peripheral blood mononuclear cells; correction of obesity candidate mutation using CRISPR to generate isogenic control iPSC; differentiation of iPSC (mutation carrying and isogenic control) into the cell type identified earlier by scRNA-Seq; and phenotypic analysis of these cells to get a molecular phenotype of the mutation. The ultimate goal is to utilize these patient-specific, functional, in vitro cell systems for drug screening and evaluation.

The vetting of genes with this database can also be used to relate CNS region–specific gene expression and circuits to other metabolic phenotypes such as T2D. Despite evidence implicating the role of the brain in glucose homeostasis, the regions of the brain involved have not all been identified and the mechanisms behind them are not fully understood (134–136). This latter use could help to deconvolute complex interactions of the CNS with seemingly remote phenotypes such as autoimmune disease and cancer.

## Methods

### Mice.

Bulk RNA-Seq experiments were performed in C57BL/6NTac male P56 mice and E14.5 embryos harvested from timed pregnant mice. All mice were obtained from Taconic Biosciences.

### Housing and diets.

Mice were housed at 22°C to 24°C temperature with a regular 12-hour light/12-hour dark cycle (lights were turned off at 7 pm), with no more than 5 adult animals per cage and ad libitum access to Purina 5058 chow diet and water.

### Tissue dissection.

P56 mice or pregnant mice with E14.5 embryos were sacrificed via cervical dislocation followed by decapitation.

For P56, brains were immediately removed and embedded in O.C.T. compound (Thermo Fisher Scientific, 23730571) and placed in dry ice–cooled isopentane to flash freeze. Each brain was sectioned into 500 μm thick coronal sections (Figure 1B, left) using a Microm HM 525 cryotome (Thermo Fisher Scientific) at –6°C. The ROIs were microdissected by single or bilateral punching of the brain sections using a dissecting microscope. The diameter of the punch, 0.5, 0.75, or 1 mm, was chosen according to the size of the region.

E14.5 embryos were embedded in O.C.T. compound and immediately placed in dry ice–cooled isopentane to flash freeze. Blocks were sectioned into 300 μm thick sagittal sections (Figure 1B, right) as described above. The ROIs were microdissected by single punches (0.5–0.75 mm diameter) of the sections.

### Sample description.

In total, 17 adult mice (P56), 12 mouse embryos (E14.5), and 2 stem cell lines were used for bulk RNA-Seq. Sample collection and preparation were performed in 9 batches within 1 month (Supplemental Table 2 and Supplemental Table 3). Depending on the size of the brain region, the number of sections punched and pooled together varied (Supplemental Table 2).

For each sample from P56 brains, dissected tissue pieces from 3 or 4 mice were immediately pooled in lysis buffer for subsequent RNA extraction (Supplemental Table 2). The only exception (due to technical issues) was that 1 nucleus accumbens (ACB) sample from only 1 mouse was processed (Supplemental Table 2). The ROI for brain areas of energy balance were as follows: frontal pole cortex, anterior cingulate area, ACB, ventral tegmental area, LHA, PVH, DMH, VMH, ARH, parabrachial nucleus, nucleus of the solitary tract, and dorsal-vagal complex (acronyms were taken from the Allen Brain Atlas; Supplemental Table 1, Supplemental Table 2, and Supplemental Figure 2). Additional brain regions (not thought to be directly involved in the regulation of energy balance) analyzed by bulk RNA-Seq were as follows: entorhinal area, CENT2, culmen lobules IV-V, uvula (IX), cerebellar nuclei, and flocculus (Supplemental Table 1; Supplemental Table 2; Supplemental Figure 1, A and B; and Supplemental Figure 2).

For each sample from E14.5 embryos, dissected tissue pieces from 4 mice were immediately pooled in lysis buffer for subsequent RNA extraction. The ROIs were THy (rostral) and PHy (caudal), forebrain, midbrain, and hindbrain (Supplemental Table 1, Supplemental Table 2, and Supplemental Figure 3).

mESC samples were included; they are not directly involved in the regulation of energy balance. Two mESC lines termed clone 1 (B6-1) and clone 2 (B6-2) were derived from a C57BL/6 mouse strain (Supplemental Table 1, Supplemental Table 3, and Supplemental Figure 1C).

### Cell lines.

mESC B6-1 and B6-2 lines were a gift from Dietrich M. Egli (Columbia University Irving Medical Center).

mESCs were maintained in a humidified incubator at 37°C on irradiated murine embryonic fibroblasts (MEFs; CF-1 MEF 4M IRR, GlobalStem) in mESC medium consisting of DMEM KO medium (catalog 10829018, Thermo Fisher Scientific) supplemented with 15% KnockOut Serum Replacement (catalog 10828028, Thermo Fisher Scientific), 0.1 mM MEM Non-Essential Amino Acids (catalog 11140050, Thermo Fisher Scientific), 2 mM GlutaMAX (catalog 35050061, Thermo Fisher Scientific), 0.06 mM 2-mercaptoethanol (catalog 21985023, Thermo Fisher Scientific), and 1000 U/mL ESGRO Leukemia Inhibitory Factor (LIF; catalog ESG1107, MilliporeSigma). Cells were passaged using TrypLe Express Enzyme (catalog 12605010, Thermo Fisher Scientific).

To obtain RNA without contamination by MEFs, mESCs were plated on gelatin-coated plates and passaged twice to remove remaining feeder cells. More specifically, cultures were collected, filtered through a 40 μm cell strainer (catalog 431750, Corning), and seeded onto gelatin-coated plates in a 1:1 mixture of mESC medium and MEF-conditioned medium (MEF-CM, prepared as below) supplemented with 3 μM CHIR-99021 (catalog S1263, Selleckchem), 0.8 μM CI-1040 (catalog S1020, Selleckchem), 0.4 μM PD0325901 (catalog S1036, Selleckchem), and 1000 U/mL LIF. mESC seeding densities were 6 × 10^5^ cells per 35 mM well for passaging and 3 × 10^5^ cells per 35 mM well for collection.

MEFs were maintained in MEF medium consisting of DMEM (catalog 11995073, Thermo Fisher Scientific) supplemented with 10% heat-inactivated FBS (catalog 10082147, Thermo Fisher Scientific). To obtain MEF-CM, MEFs were incubated with mESC medium for 24 hours. MEF-CM was collected daily and filtered through a 45 μm filter (catalog SCHVU01RE, MilliporeSigma).

### RNA extraction.

For all punches collected from P56 and E14.5 mice, total RNA was extracted using PicoPure RNA Isolation Kit (Thermo Fisher Scientific, KIT0204) with on-column DNase I (QIAGEN, 79254) treatment to remove genomic DNA contamination; RNA was stored at –80°C until further processing.

mESC were homogenized in TRIzol reagent (catalog 15596026, Thermo Fisher Scientific) and total RNA was extracted using RNeasy Plus Micro Kit (catalog 74034, QIAGEN) with on-column DNase I treatment; RNA was stored at –80°C until further processing.

An Agilent 2100 Bioanalyzer was used to assess the total RNA quality. Agilent 2100 Expert software (version B.02.08.SI648) was used with the Eukaryote Total RNA Nano Series II assay settings. Only RNA samples with an RNA integrity number greater than or equal to 7.6 were sequenced.

### Library preparation and sequencing.

Strand-specific RNA-Seq libraries were prepared using the KAPA mRNA-Seq Library Preparation Kit (Kapa Biosystems). Twelve-cycle PCR was performed to amplify libraries. Sequencing was performed on Illumina HiSeq2000 by multiplexed single-read run with 33 cycles. Raw sequence data (BCL files) were converted to FASTQ format via Illumina Casava 1.8.2. Reads were decoded based on their barcodes and read quality was evaluated with FastQC (137).

### Data analysis.

All samples were aligned to mouse assembly GRCm38 cDNA sequence downloaded from Ensembl with Kallisto (ref. 138; version 0.43.1; fragment size set as 120 with SD as 20, default seed as 42, with 100 bootstrap per sample). For the transcript model, genome Ensembl GRCm38 release was used. Gene level expression were obtained by summing transcript abundances (TPM units).

Raw data and Kallisto output can be found at NCBI Gene Expression Omnibus (accession GSE178290; https://www.ncbi.nlm.nih.gov/geo/query/acc.cgi?acc=GSE178290).

Principal component analysis was used for outlier detection.

Hierarchical clustering was used to determine the how the regions cluster together (Figure 2A) and show gene order for most heatmaps. Genes were routinely visualized with heatmaps, which were produced by averaging the TPM data across the replicates for each brain region and for mESC (replicates’ details are given in Supplemental Table 2 and Supplemental Table 3). The TPM values for each gene were scaled for each gene to improve visualization.

To calculate the frequency distribution, the TPM of each gene was calculated as a percentage compared with the total TPM values for all samples (brain regions and mESC, averaged across each replicate). The frequency score and standard error was calculated by compiling the percentage for all the genes in a gene list.

GSEA (v 4.1.0; ref. 139) was used to complement the frequency distribution. All replicates were input, alongside either the obesity gene lists (monogenic, rare coding variants, low-frequency coding variants, GWAS, and syndromic) or the broader gene list (obesity, T1D, T2D, autism, and breast cancer). GSEA generated an enrichment score, which indicated whether the genes within a particular gene list were ranked highly in the sample compared with all other samples (Supplemental Figure 6 and Supplemental Figure 11), within all the genes from the gene lists input, and the enrichment score was plotted.

WGCNA (v1.70-3; ref. 140) was used to see how obesity genes cluster and how they cluster with other disease genes. This allows for determination of genes with similar expression patterns within the data set and consideration of how genes are expressed across the brain regions (e.g., homeostatic regions). Replicates of the same brain region were averaged and then a soft thresholding power was chosen to give the highest separation between modules. WGCNA outputs discrete modules and a dendrogram (Supplemental Figure 7 and Supplemental Figure 12). Heatmaps were used for visualization of the modules. The order of genes on the heatmap was determined by the module number, followed by the order the gene appears in hierarchical clustering. To determine if a gene list is enriched within a module, we developed an overrepresentation score, which indicates the deviation from an equal distribution across modules, normalizing for module size and gene list size. Overrepresentation score was calculated using the following equation: (w/[x/y] × z) – 1, where w = *n* genes per module per gene list, x = *n* genes in gene list, y = *n* all gene lists, and z = *n* genes in module. This overrepresentation score was plotted to show a positive deflection where a gene list is overrepresented.

K-means clustering was used to partition *n* observations into *k* clusters, in which each observation belongs to the cluster with the nearest mean (141). The number of clusters was decided based on the slope change on an elbow plot. Enrichment was determined using the same method used for WGCNA, where a k-means cluster is substituted for a WGCNA module.

Sequencing data are available on an interactive web portal, the Brain Energy Balance Atlas, which is accessible via http://doegelab.com This portal allows users to search for expression of any annotated gene and its differential expression profiles across the 22 brain regions and 2 mESC lines studied. Counts files were analyzed with DESeq2 (ref. 142; version 1.28.1) to compare the expression pattern in each brain region with all other brain regions or mESCs.

### Statistics.

Frequency distribution was expressed as mean ± SEM. For each gene list, pairwise comparisons of all brain regions were evaluated using 1-way ANOVA and reporting the Benjamini, Krieger, and Yekutieli 2-step false discovery rate–corrected *P* value. *P* values of less than 0.05 were considered significant. For GSEA enrichment score plots, regions with a positive enrichment score and an FDR adjusted *P* value of less than 0.05 were denoted with an asterisk (139). All analyses were performed in R (v 1.3.1093) or in GraphPad Prism (v 9).

### Study approval.

Animal care and experimental procedures were performed according to Columbia University animal welfare guidelines and approved by the Columbia University IACUC, protocol number AC-AAAS4459.

## Author contributions

MCDR, JYA, and CAD designed the studies and interpreted the data. GS provided advice on experimental design. MCDR, GS, and CAL performed the experiments. QS and JYA performed the RNA sequencing. HJG performed the data analyses. MCDR wrote the manuscript. HJG, GS, CAL, QS, YS, MWS, WKC, RLL, JYA, and CAD reviewed and edited the manuscript.

## Supplementary Material

Supplemental data

Supplemental Table 6

Supplemental Table 14

## Version 1 07/20/2021

In-Press Preview

## Version 2 08/23/2021

Electronic publication
