# Supplementary material for: Gene expression atlas of energy balance brain regions
Source: JCI Insight. 2021 Aug 23;6(16):e149137. doi: 10.1172/jci.insight.149137 (PMC8409984; doi:10.1172/jci.insight.149137)
Supplement: Supplemental data [file jciinsight-6-149137-s140.pdf]

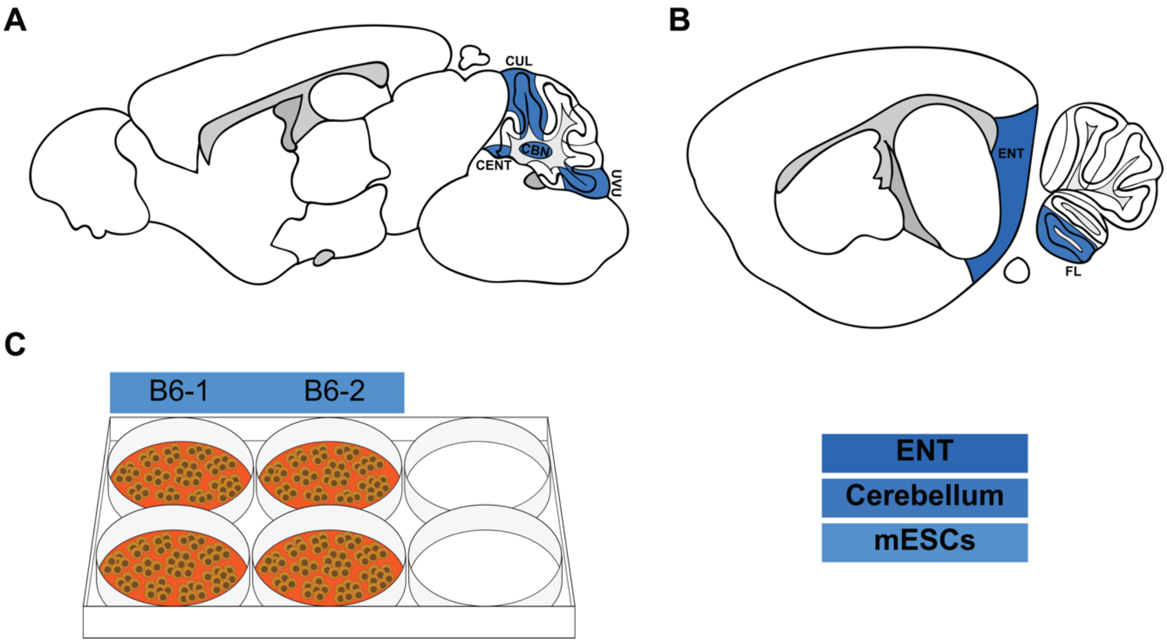

Supplemental Figure 1 | Additional brain regions and mESC analyzed by bulk RNA-seq. (A) Regions of the cerebellum: culmen lobules IV-V (CUL4,5), central lobule II (CENT2), uvula (IX) (UVU), and cerebellar nuclei (CBN). **(B)** Entorhinal area (ENT) and flocculus (FL). **(C)** Mouse embryonic stem cells, C57BL/6 clone 1 (B6-1) and clone 2 (B6-2).

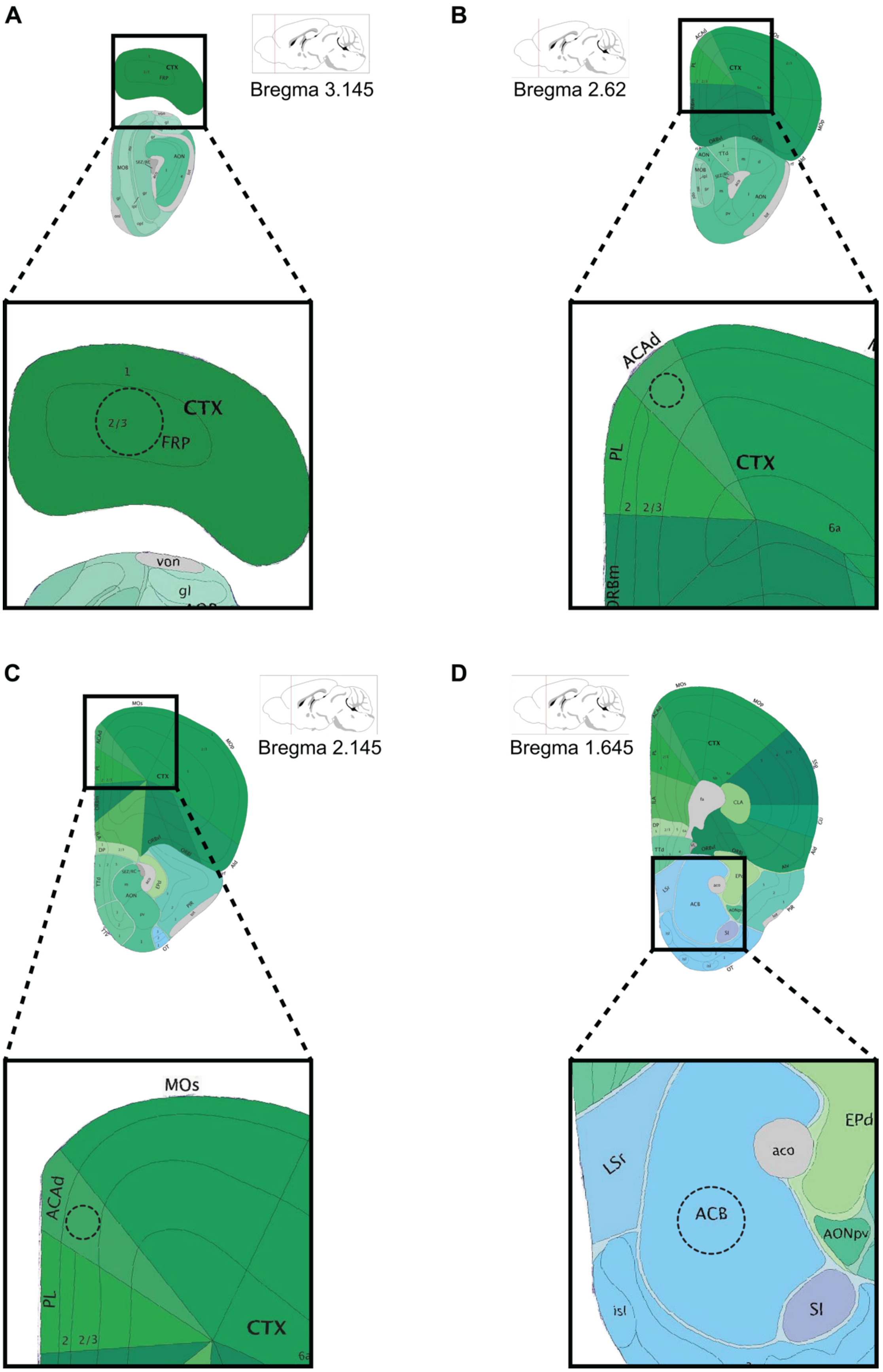
A
Bregma 3.145
CTX
2/3
FRP
1
von
gl
B
Bregma 2.62
ACAd
PL
CTX
2
2/3
6a
ORBm
C
Bregma 2.145
MOs
ACAd
PL
CTX
2
2/3
D
Bregma 1.645
LSr
ACB
aco
EPd
AONpv
SI
isl

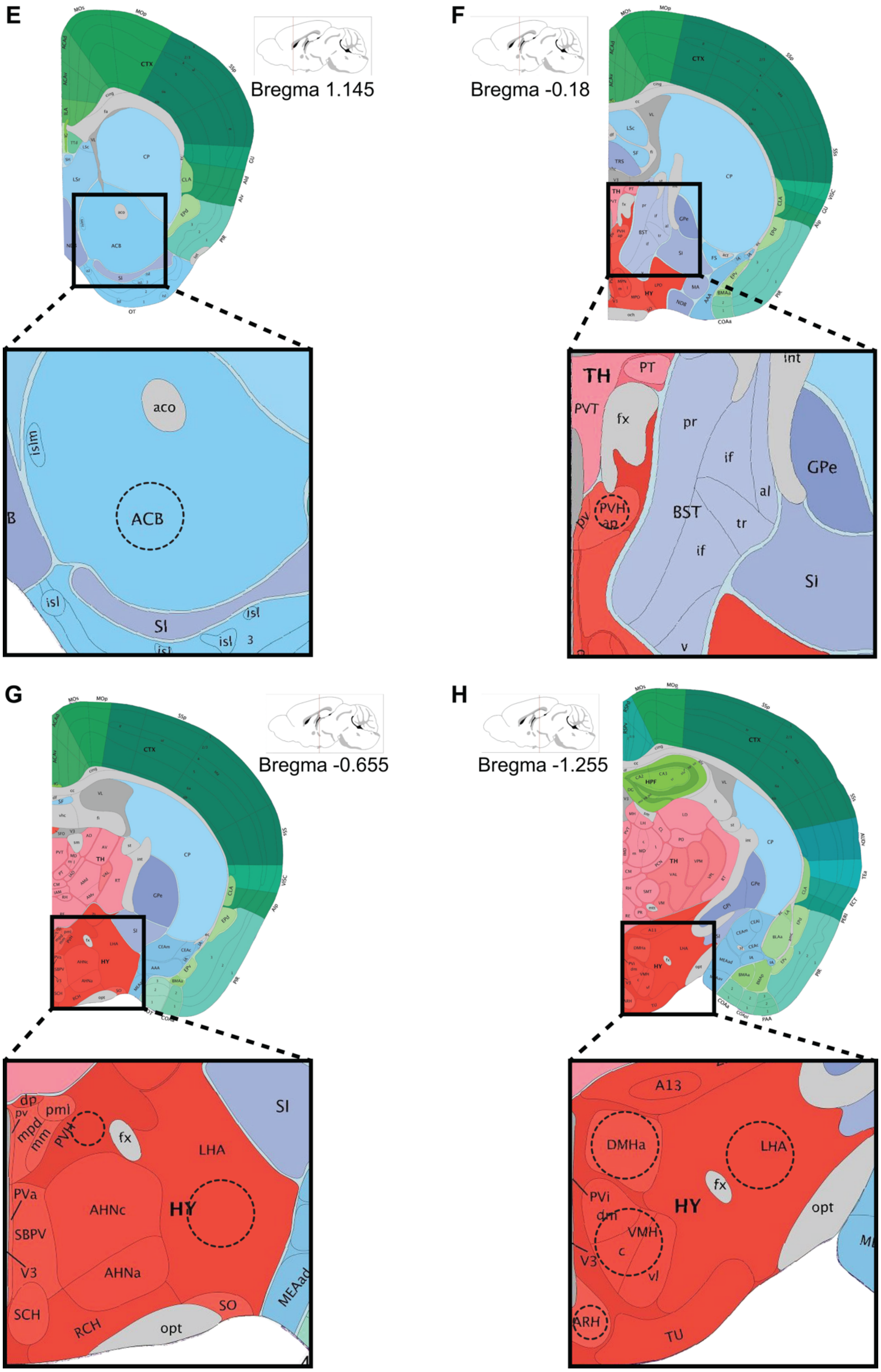
E
Bregma 1.145
aco
islm
ACB
B
isl
SI
isl
isl
3
F
Bregma -0.18
TH
PT
int
PVT
fx
pr
if
GPe
al
PVH
ap
pv
BST
tr
if
SI
v
G
Bregma -0.655
SI
dp
pv
pml
mpd
mm
PVH
fx
LHA
PVa
AHNc
HY
SBPV
V3
AHNa
SCH
RCH
opt
SO
MEAad
H
Bregma -1.255
A13
DMHa
LHA
fx
PVi
HY
opt
dm
VMH
c
V3
vl
ARH
TU

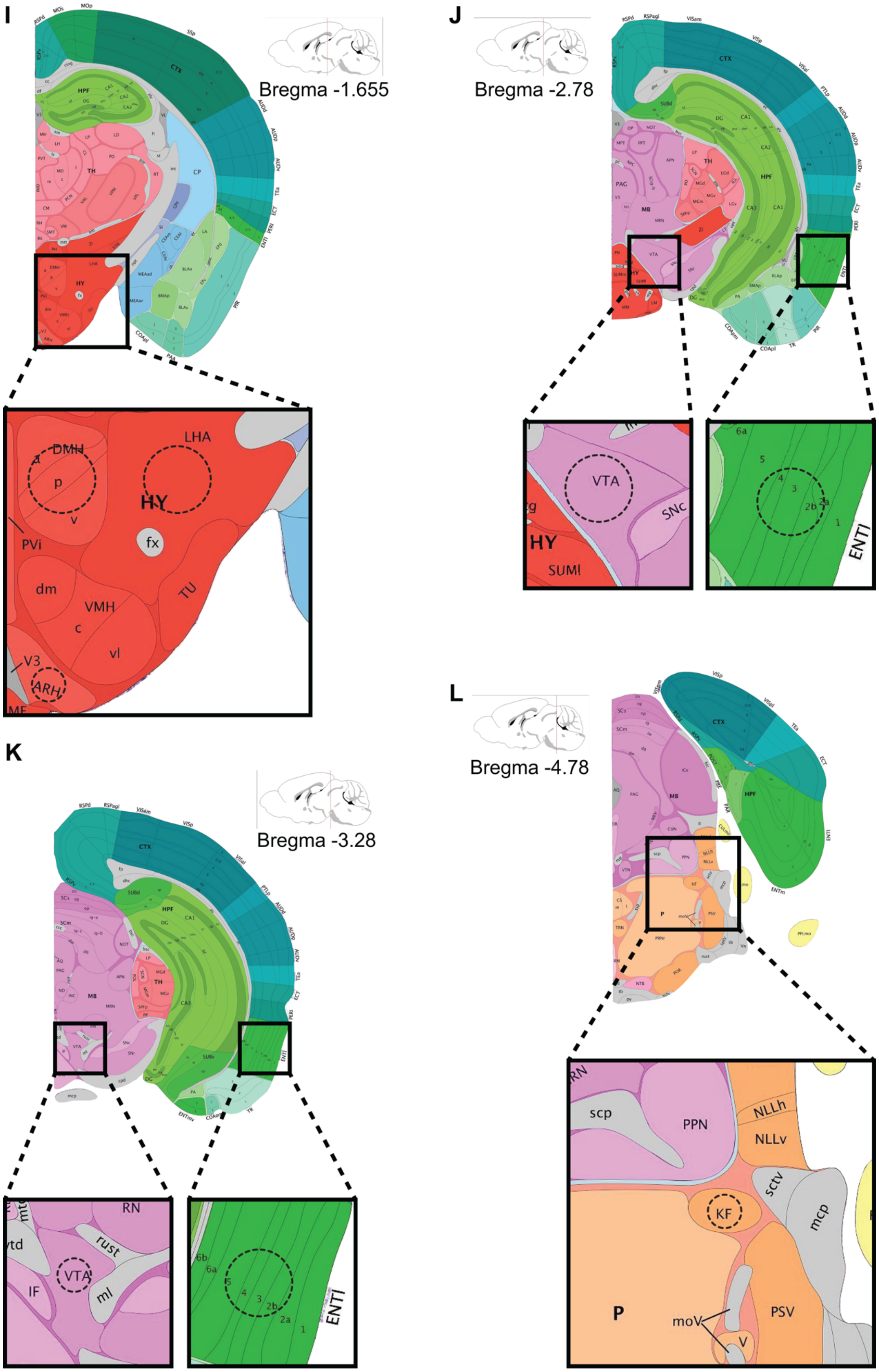
I
Bregma -1.655
DMH
a
p
v
LHA
HY
PVi
fx
dm
VMH
c
vl
TU
V3
ARH
ME
J
Bregma -2.78
VTA
SNc
HY
SUMl
6a
5
4
3
2b
2a
1
ENTl
K
Bregma -3.28
RN
VTA
rust
vtd
IF
ml
6b
6a
5
4
3
2b
2a
1
ENTl
L
Bregma -4.78
scp
PPN
NLLh
NLLv
sctv
mcp
KF
P
moV
V
PSV

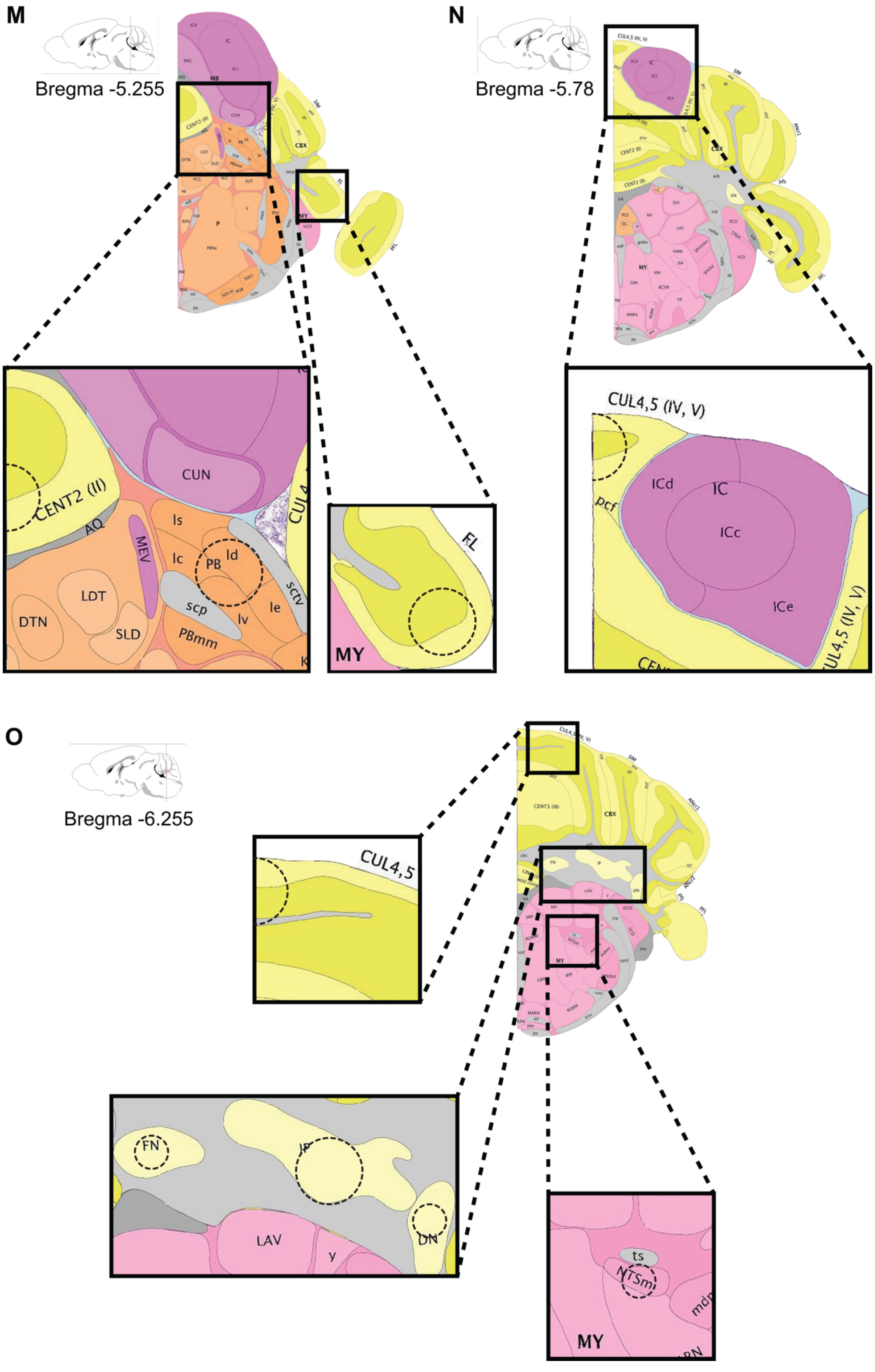
M
Bregma -5.255
CENT2 (II)
AQ
CUN
MEV
ls
lc
PB
ld
LDT
DTN
SLD
scp
lv
le
PBmm
sctv
FL
MY
N
Bregma -5.78
CUL4,5 (IV, V)
pcf
ICd
IC
ICc
ICe
O
Bregma -6.255
CUL4,5
FN
IP
DN
LAV
y
ts
NTSm
MY

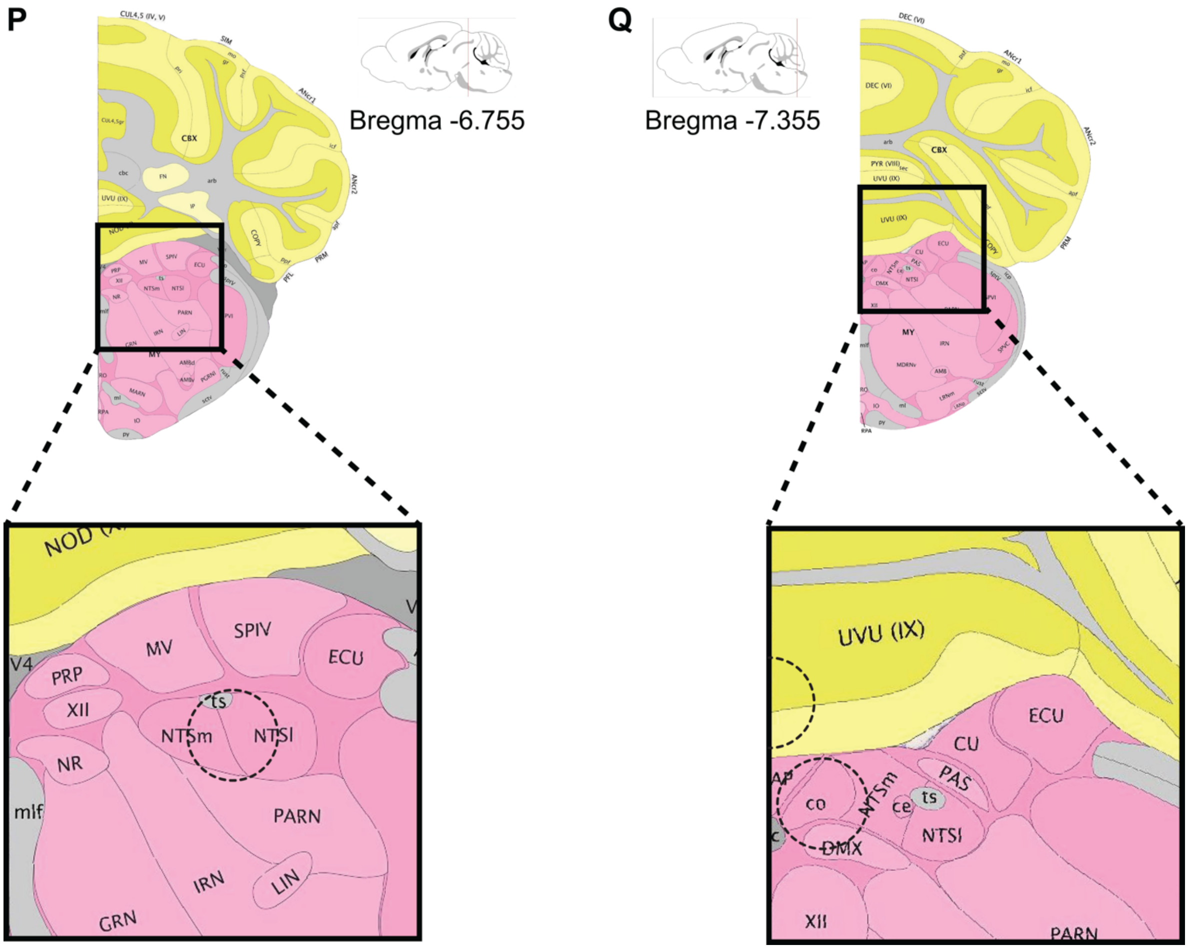

Supplemental Figure 2 | Coronal sections showing the regions in the adult mouse brain analyzed by bulk RNA-seq. The bregma distance for each slice is indicated. The boxed portion of the coronal hemispheric section in the top panels are enlarged and shown in the bottom panels (**A** to **Q**, adapted from Allen Brain Atlas, P56). **A**, FRP; **B** and **C**, ACA; **D** and **E**, ACB; **F**, PVH; **G**, PVH and LHA; **H**, DMH, VMH, ARH, LHA; **I**, DMH, ARH, LHA; **J** and **K**, VTA (left), ENT (right); **L**, PB; **M**, CENT2 and PB (left), FL (right); **N**, CUL4,5; **O**, from the top to the bottom, CUL4,5, CBN, NTS; **P**, NTS; **Q**, UVU, DVC.

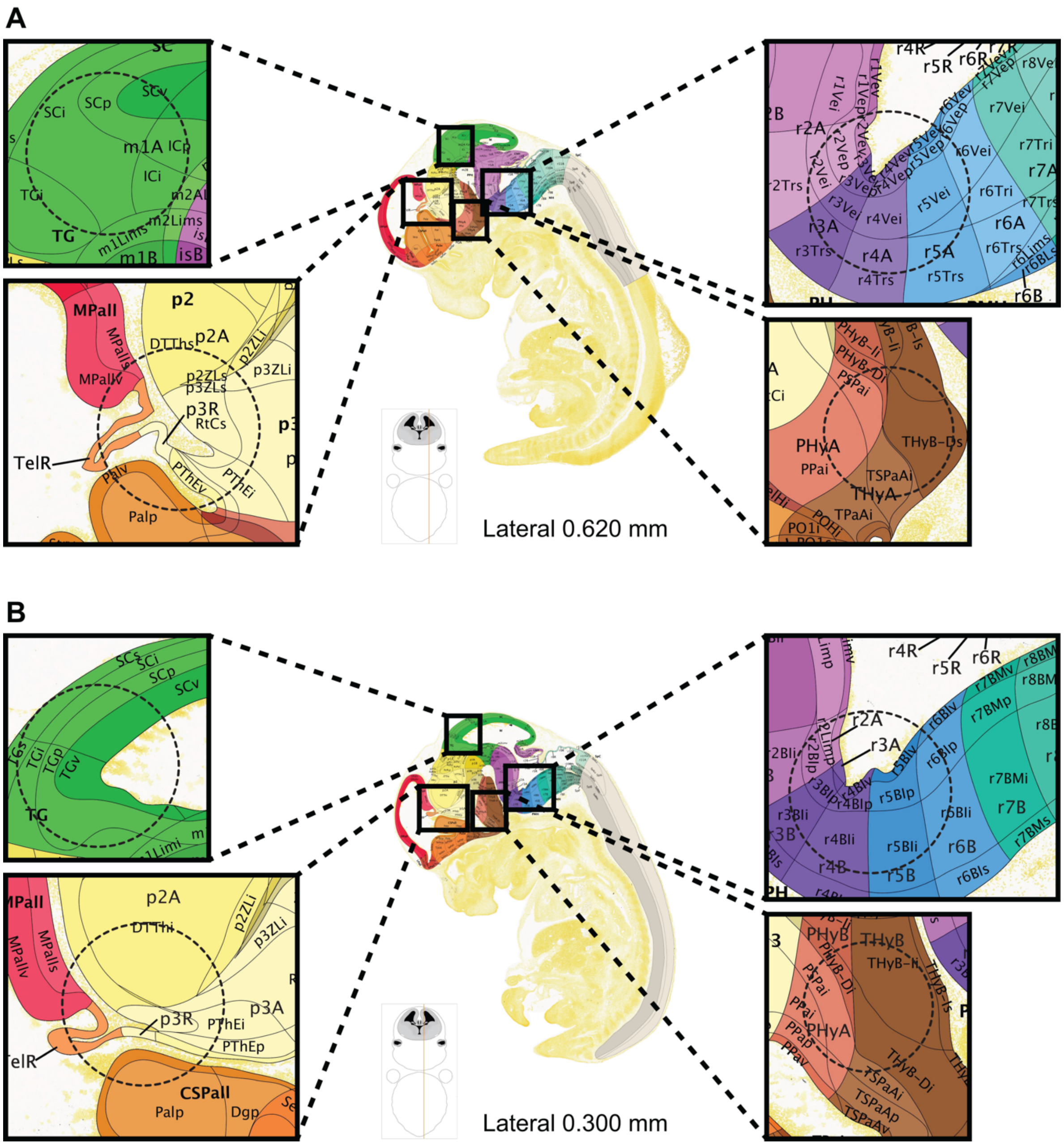

Supplemental Figure 3 | Sagittal sections showing the regions in the embryonic brain analyzed by bulk RNA-seq. The boxed regions in the central figures are enlarged and shown on both sides (**A** and **B**, adapted from Allen Brain Atlas, E13.5). **A** and **B**, top left M; bottom left F; top right H; bottom right THy, PHy.

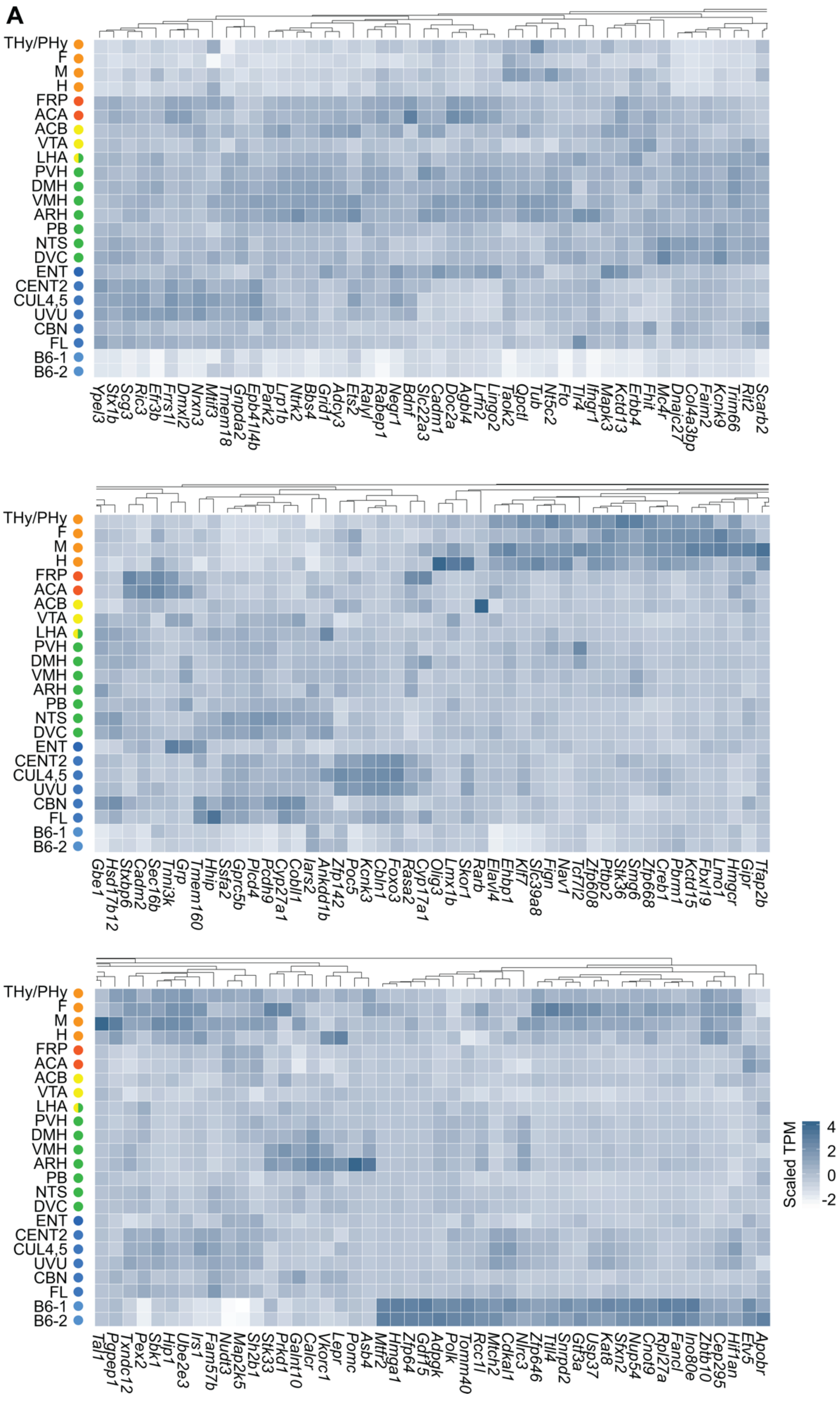
A
THy/PHy
F
M
H
FRP
ACA
ACB
VTA
LHA
PVH
DMH
VMH
ARH
PB
NTS
DVC
ENT
CENT2
CUL4,5
UVU
CBN
FL
B6-1
B6-2
Scaled TPM
4
2
0
-2

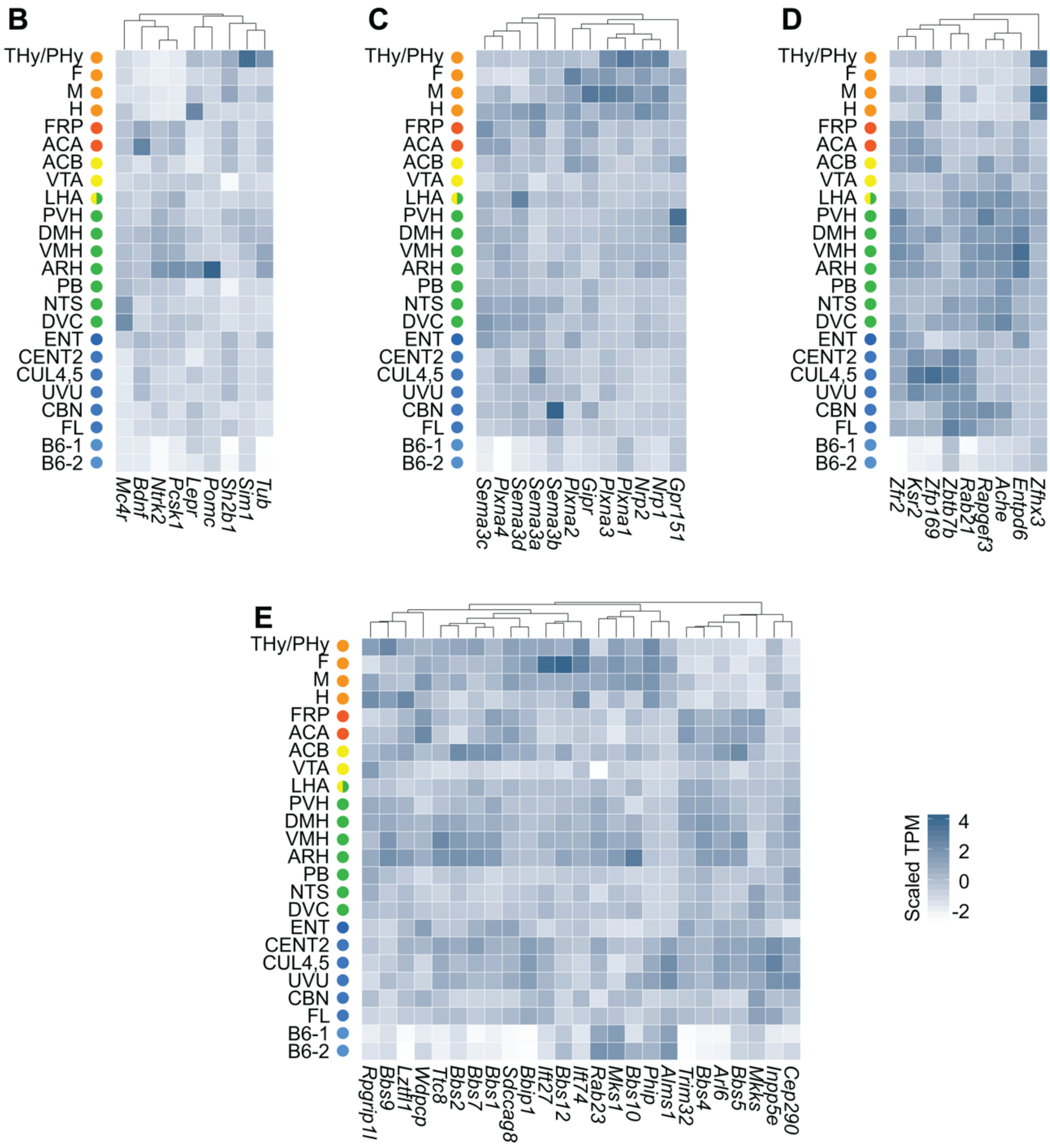

Supplemental Figure 4 | Expression profiling of obesity-associated genes throughout the brain regions of energy balance. Scaled TPM values are indicated in heat maps where the darker the blue, the higher the expression. **(A)** Expression heat maps of genes identified by GWAS of BMI. **(B)** Heat map of genes associated with monogenic obesity. **(C)** Heat map of genes associated with rare coding variants associated with BMI. **(D)** Heat map of genes associated with low-frequency coding variants associated with BMI. **(E)** Heat map of genes associated with syndromic obesity. Genes were sorted by hierarchical clustering of scaled genes.

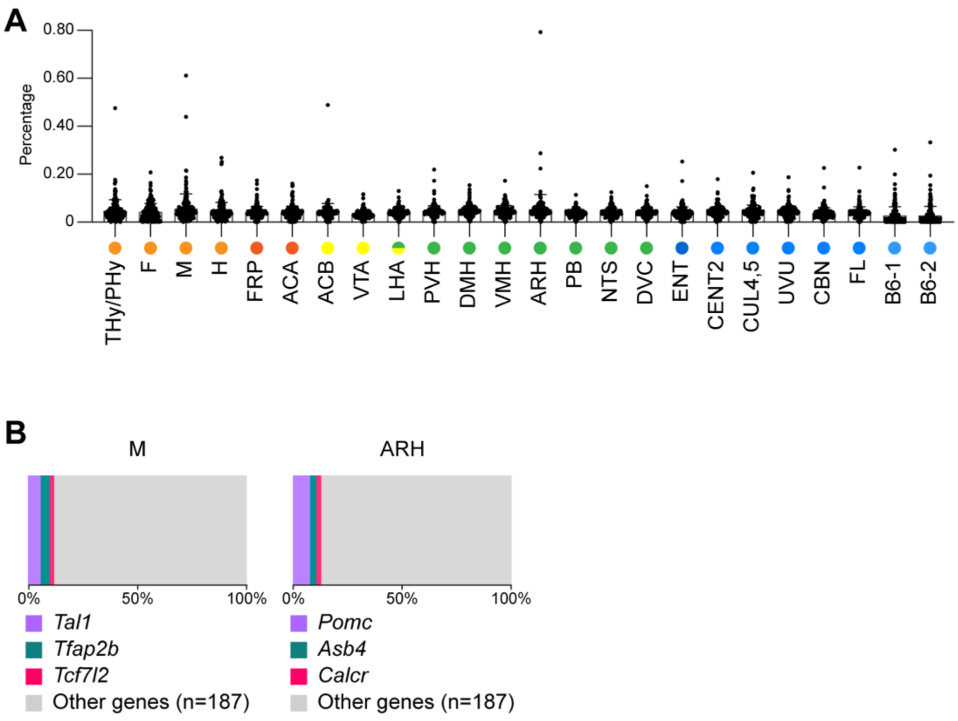

Supplemental Figure 5 | Frequency distribution of all obesity-associated genes across the brain regions of energy balance. (A) Frequency distributions for each obesity-associated gene was determined by calculating the TPM percentage for each region compared to the sum of TPM values for all samples (brain regions and mESC, averaged across each replicate). For each genetic category, the frequency score and standard error was calculated by compiling the percentage for all the genes in a gene list, and shown as mean ± SE. Individual percentage values for each gene was displayed as dot. N = 190. **(B)** Detailed analysis of the two most enriched brain regions (embryonic midbrain (M) and ARH). Visualization of the 3 genes with the highest percentage expression. Each slice represent the percentage of expression for a specific gene or group of genes (as indicated in the legend), and the sum of all the slices represent the total expression (100%) of genes for a specific genetic category in a selected brain region.

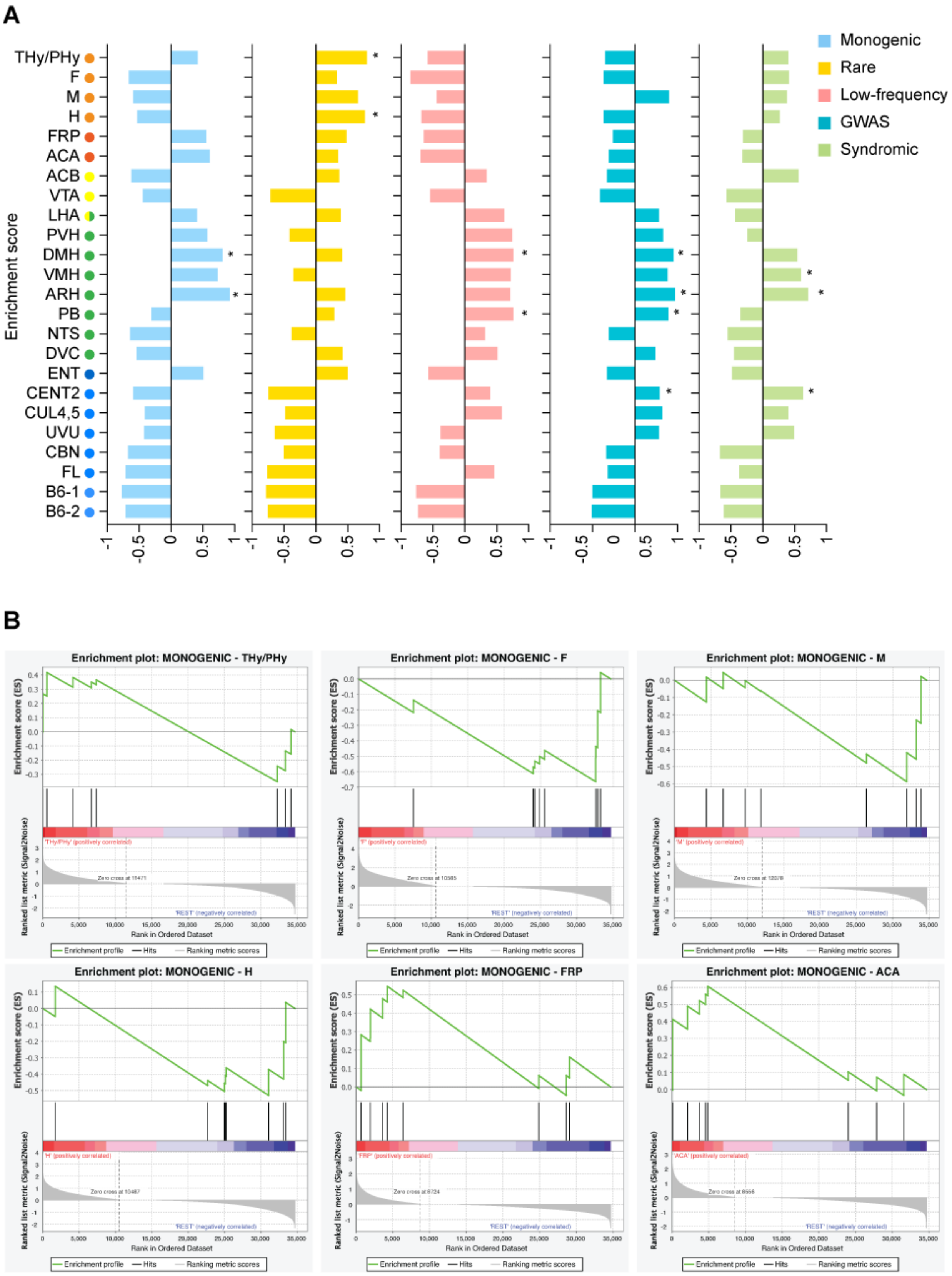
A
Monogenic
Rare
Low-frequency
GWAS
Syndromic
Enrichment score
THy/PHy
F
M
H
FRP
ACA
ACB
VTA
LHA
PVH
DMH
VMH
ARH
PB
NTS
DVC
ENT
CENT2
CUL4,5
UVU
CBN
FL
B6-1
B6-2
B
Enrichment plot: MONOGENIC - THy/PHy
Enrichment plot: MONOGENIC - F
Enrichment plot: MONOGENIC - M
Enrichment plot: MONOGENIC - H
Enrichment plot: MONOGENIC - FRP
Enrichment plot: MONOGENIC - ACA
Enrichment score (ES)
Ranked list metric (Signal2Noise)
Rank in Ordered Dataset
Enrichment profile
Hits
Ranking metric scores

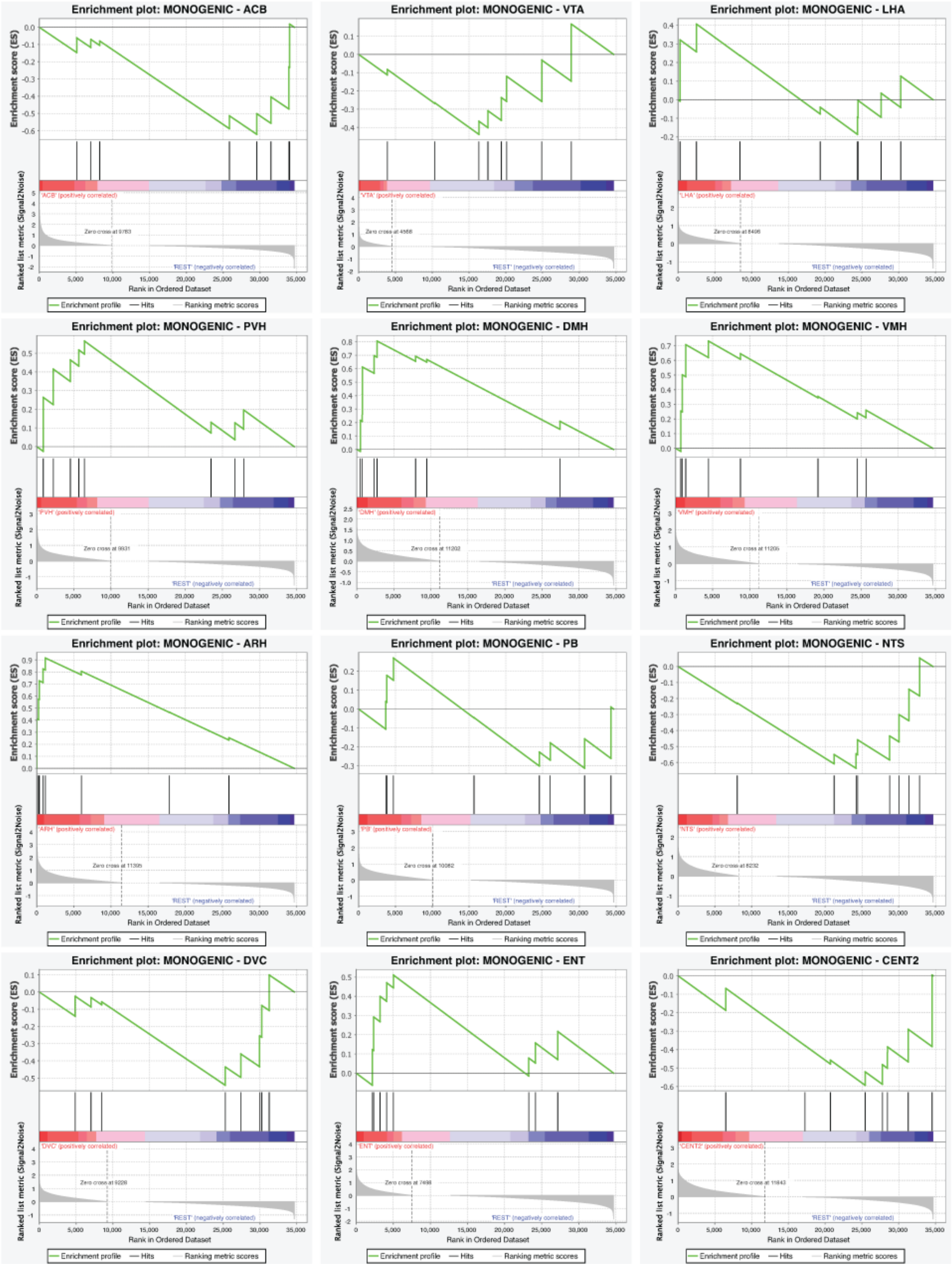
Enrichment plot: MONOGENIC - ACB
Enrichment plot: MONOGENIC - VTA
Enrichment plot: MONOGENIC - LHA
Enrichment plot: MONOGENIC - PVH
Enrichment plot: MONOGENIC - DMH
Enrichment plot: MONOGENIC - VMH
Enrichment plot: MONOGENIC - ARH
Enrichment plot: MONOGENIC - PB
Enrichment plot: MONOGENIC - NTS
Enrichment plot: MONOGENIC - DVC
Enrichment plot: MONOGENIC - ENT
Enrichment plot: MONOGENIC - CENT2
Enrichment score (ES)
Ranked list metric (Signal2Noise)
Rank in Ordered Dataset
Enrichment profile
Hits
Ranking metric scores

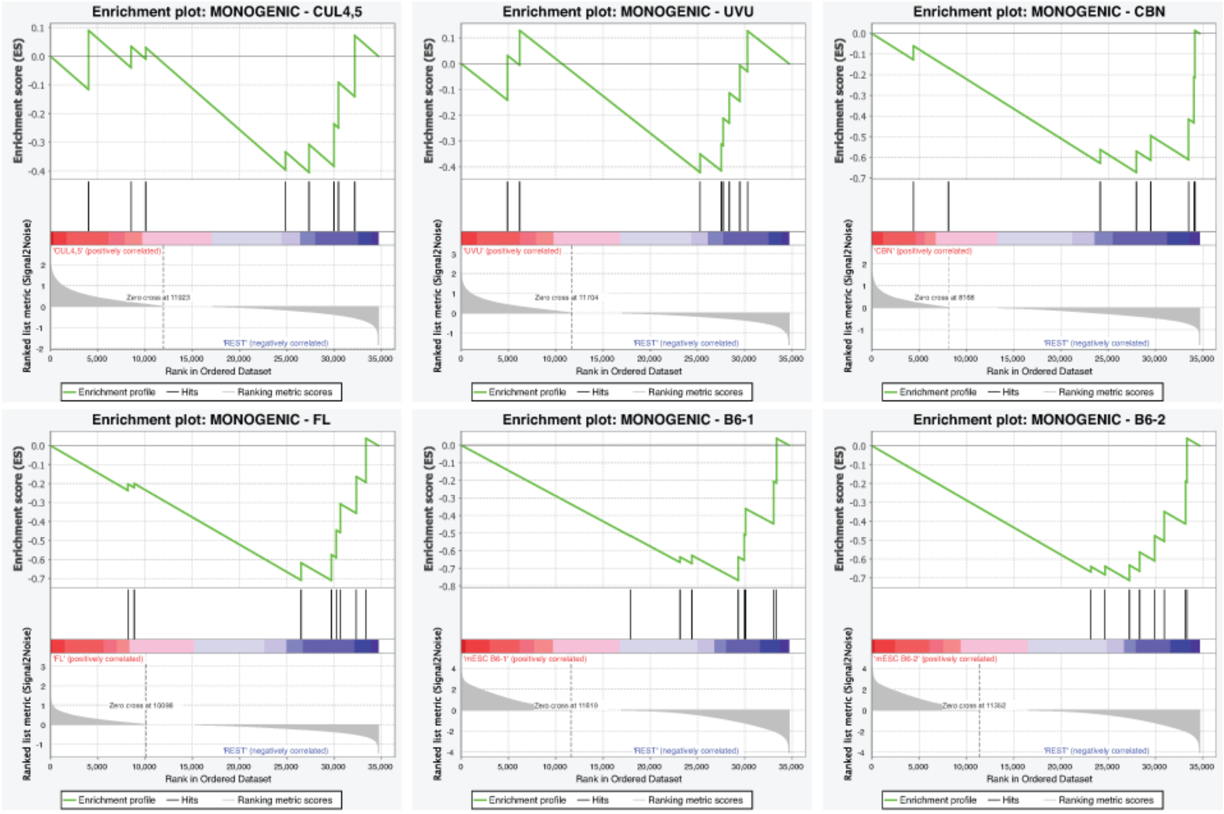
Enrichment plot: MONOGENIC - CUL4,5
Enrichment plot: MONOGENIC - UVU
Enrichment plot: MONOGENIC - CBN
Enrichment plot: MONOGENIC - FL
Enrichment plot: MONOGENIC - B6-1
Enrichment plot: MONOGENIC - B6-2
Enrichment score (ES)
Ranked list metric (Signal2Noise)
Rank in Ordered Dataset
Enrichment profile
Hits
Ranking metric scores

C

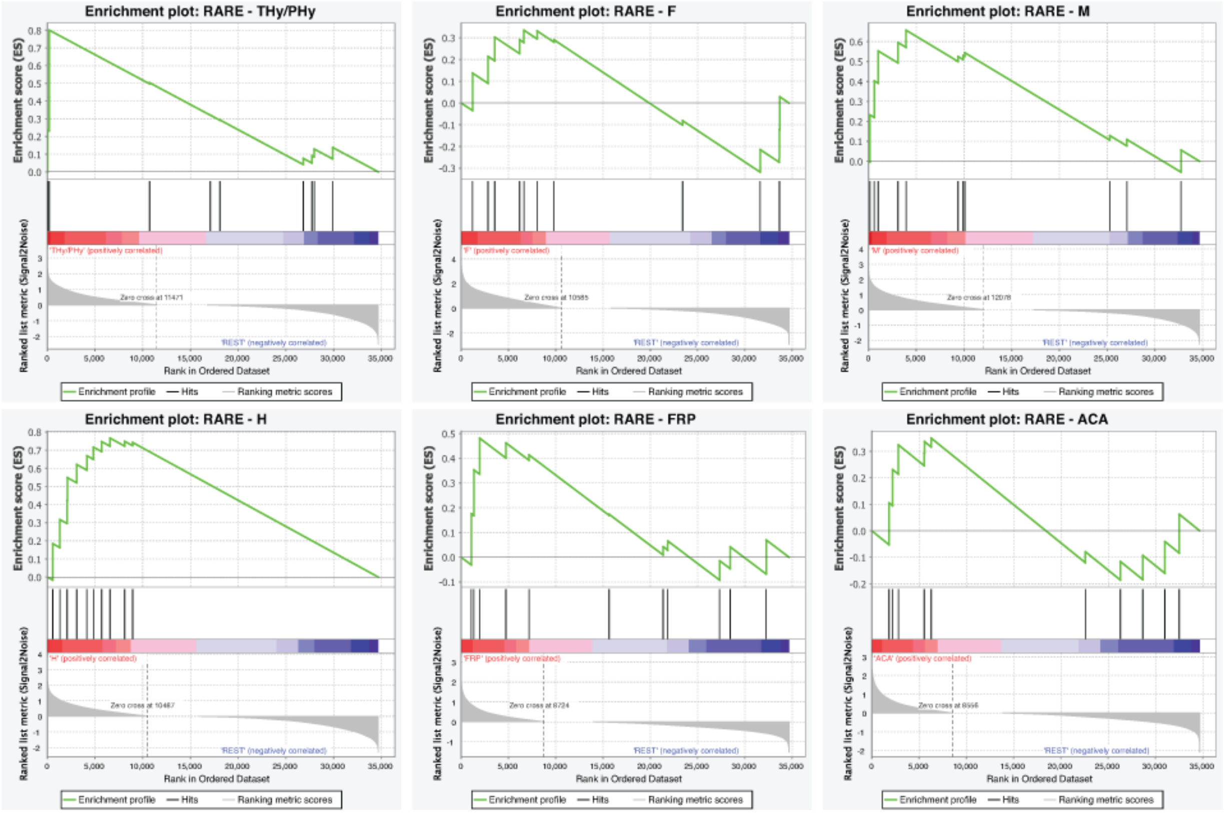
Enrichment plot: RARE - THy/PHy
Enrichment plot: RARE - F
Enrichment plot: RARE - M
Enrichment plot: RARE - H
Enrichment plot: RARE - FRP
Enrichment plot: RARE - ACA
Enrichment score (ES)
Ranked list metric (Signal2Noise)
Rank in Ordered Dataset
Enrichment profile
Hits
Ranking metric scores

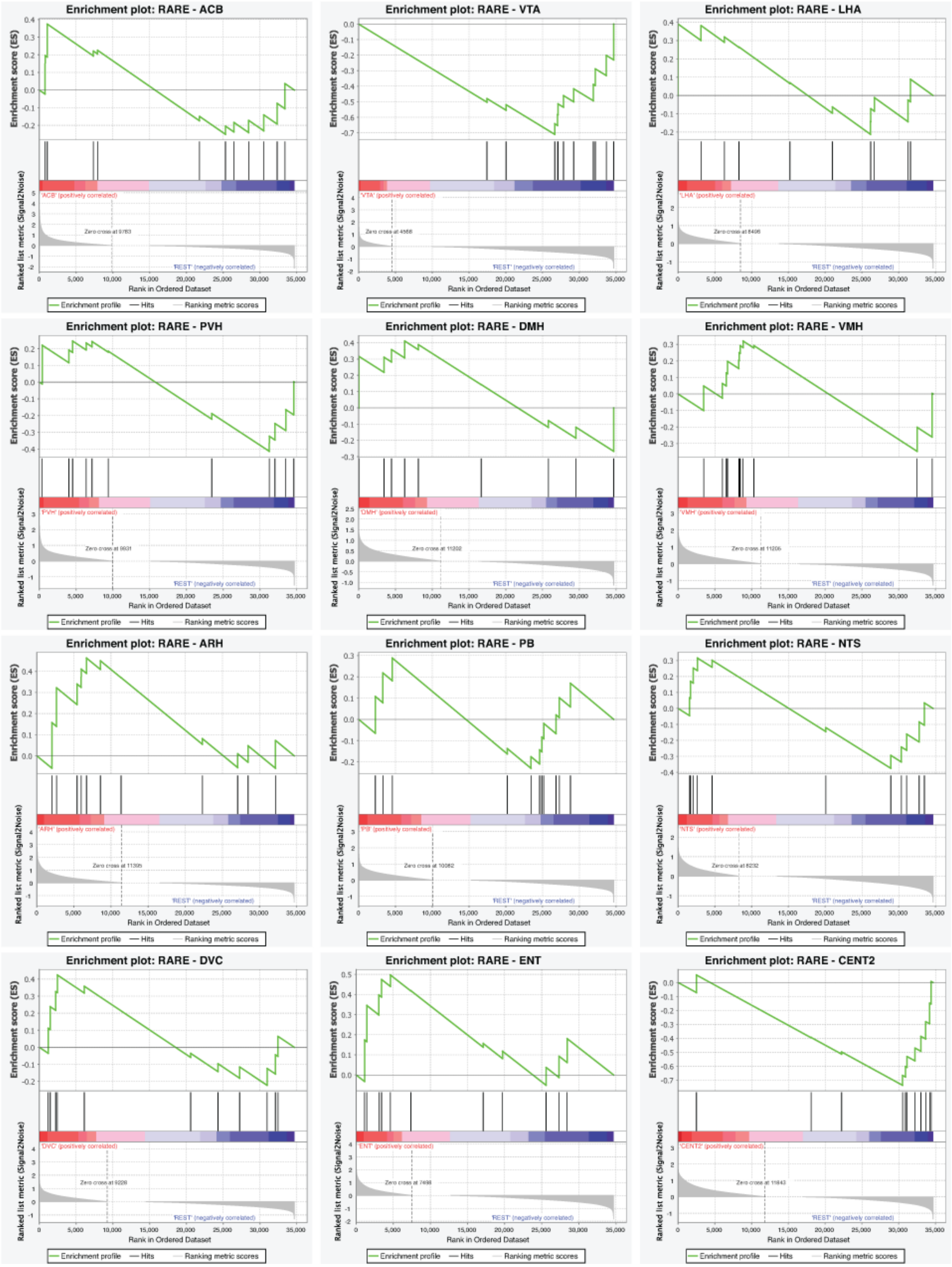
Enrichment plot: RARE - ACB
Enrichment plot: RARE - VTA
Enrichment plot: RARE - LHA
Enrichment plot: RARE - PVH
Enrichment plot: RARE - DMH
Enrichment plot: RARE - VMH
Enrichment plot: RARE - ARH
Enrichment plot: RARE - PB
Enrichment plot: RARE - NTS
Enrichment plot: RARE - DVC
Enrichment plot: RARE - ENT
Enrichment plot: RARE - CENT2
Enrichment score (ES)
Ranked list metric (Signal2Noise)
Rank in Ordered Dataset
Enrichment profile
Hits
Ranking metric scores

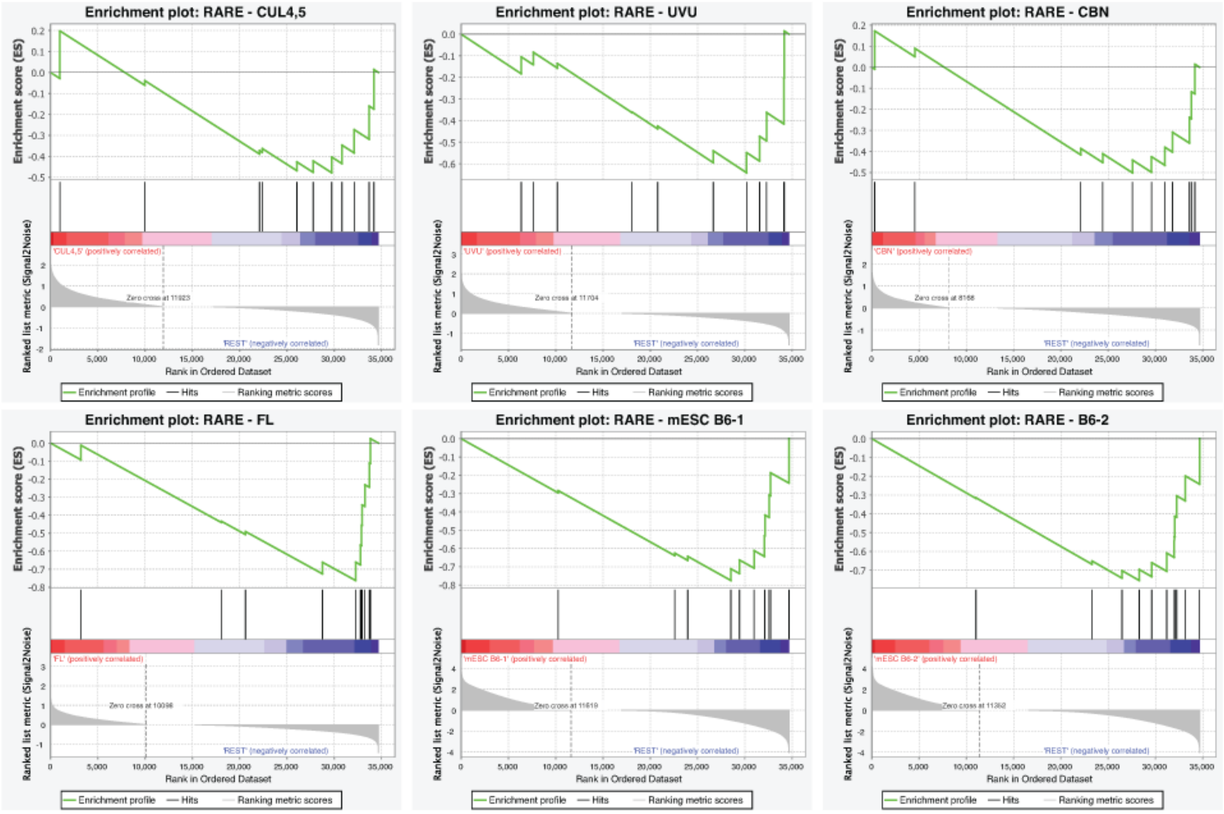
Enrichment plot: RARE - CUL4,5
Enrichment plot: RARE - UVU
Enrichment plot: RARE - CBN
Enrichment plot: RARE - FL
Enrichment plot: RARE - mESC B6-1
Enrichment plot: RARE - B6-2
Enrichment score (ES)
Ranked list metric (Signal2Noise)
Rank in Ordered Dataset
Enrichment profile
Hits
Ranking metric scores

D

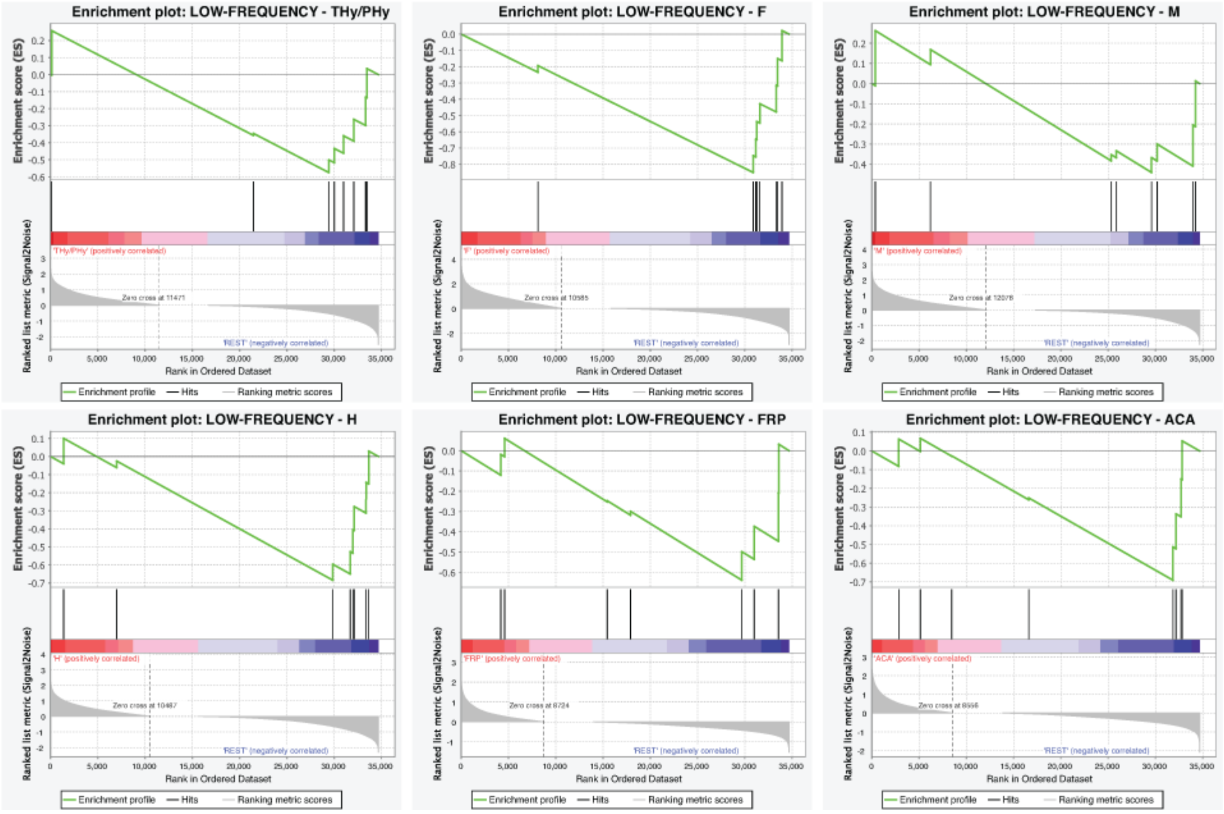
Enrichment plot: LOW-FREQUENCY - THy/PHy
Enrichment plot: LOW-FREQUENCY - F
Enrichment plot: LOW-FREQUENCY - M
Enrichment plot: LOW-FREQUENCY - H
Enrichment plot: LOW-FREQUENCY - FRP
Enrichment plot: LOW-FREQUENCY - ACA
Enrichment score (ES)
Ranked list metric (Signal2Noise)
Rank in Ordered Dataset
Enrichment profile
Hits
Ranking metric scores

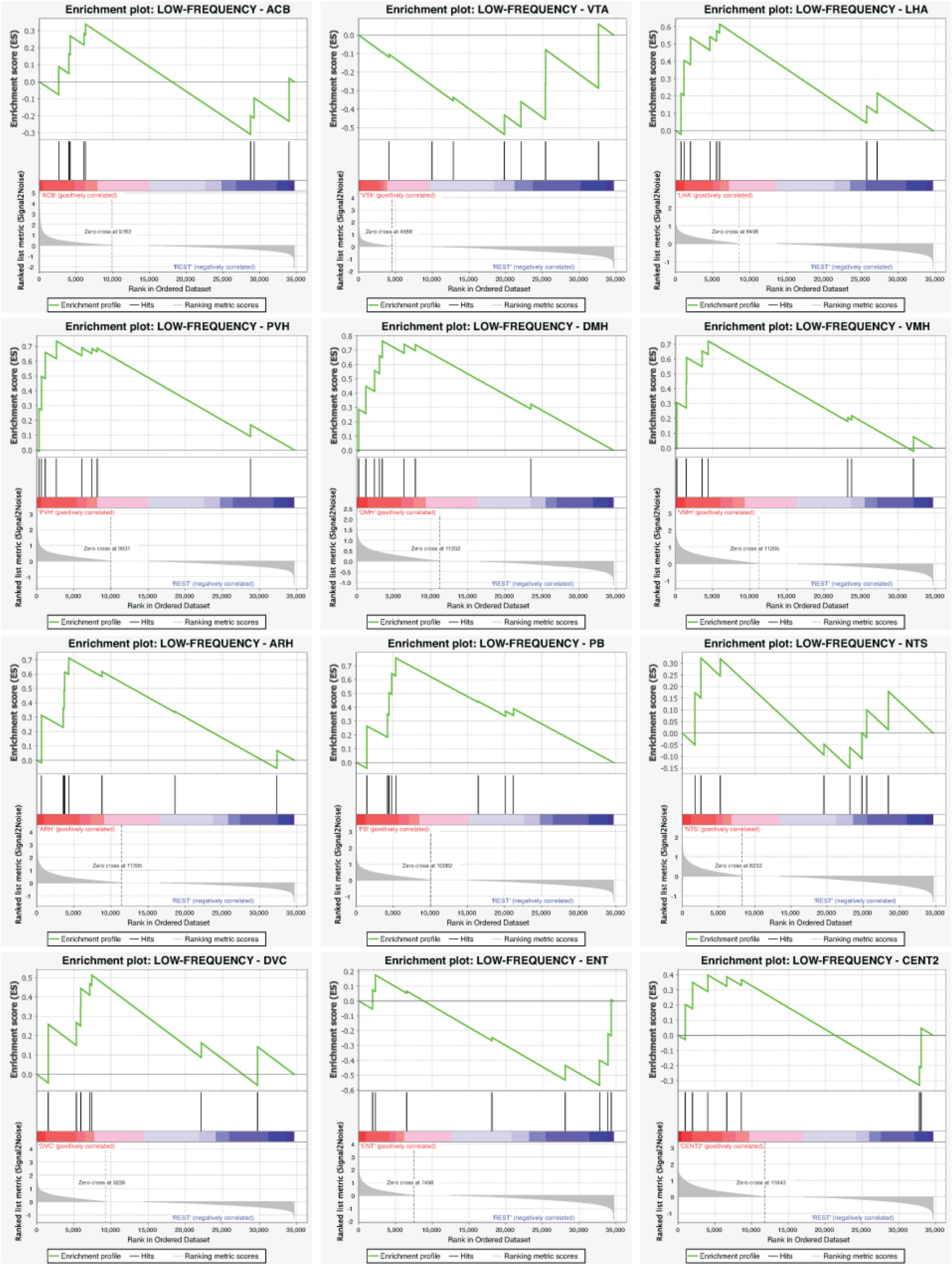

Enrichment plot: LOW-FREQUENCY - ACB
Enrichment plot: LOW-FREQUENCY - VTA
Enrichment plot: LOW-FREQUENCY - LHA
Enrichment plot: LOW-FREQUENCY - PVH
Enrichment plot: LOW-FREQUENCY - DMH
Enrichment plot: LOW-FREQUENCY - VMH
Enrichment plot: LOW-FREQUENCY - ARH
Enrichment plot: LOW-FREQUENCY - PB
Enrichment plot: LOW-FREQUENCY - NTS
Enrichment plot: LOW-FREQUENCY - DVC
Enrichment plot: LOW-FREQUENCY - ENT
Enrichment plot: LOW-FREQUENCY - CENT2
Enrichment score (ES)
Ranked list metric (Signal2Noise)
Rank in Ordered Dataset
Enrichment profile
Hits
Ranking metric scores

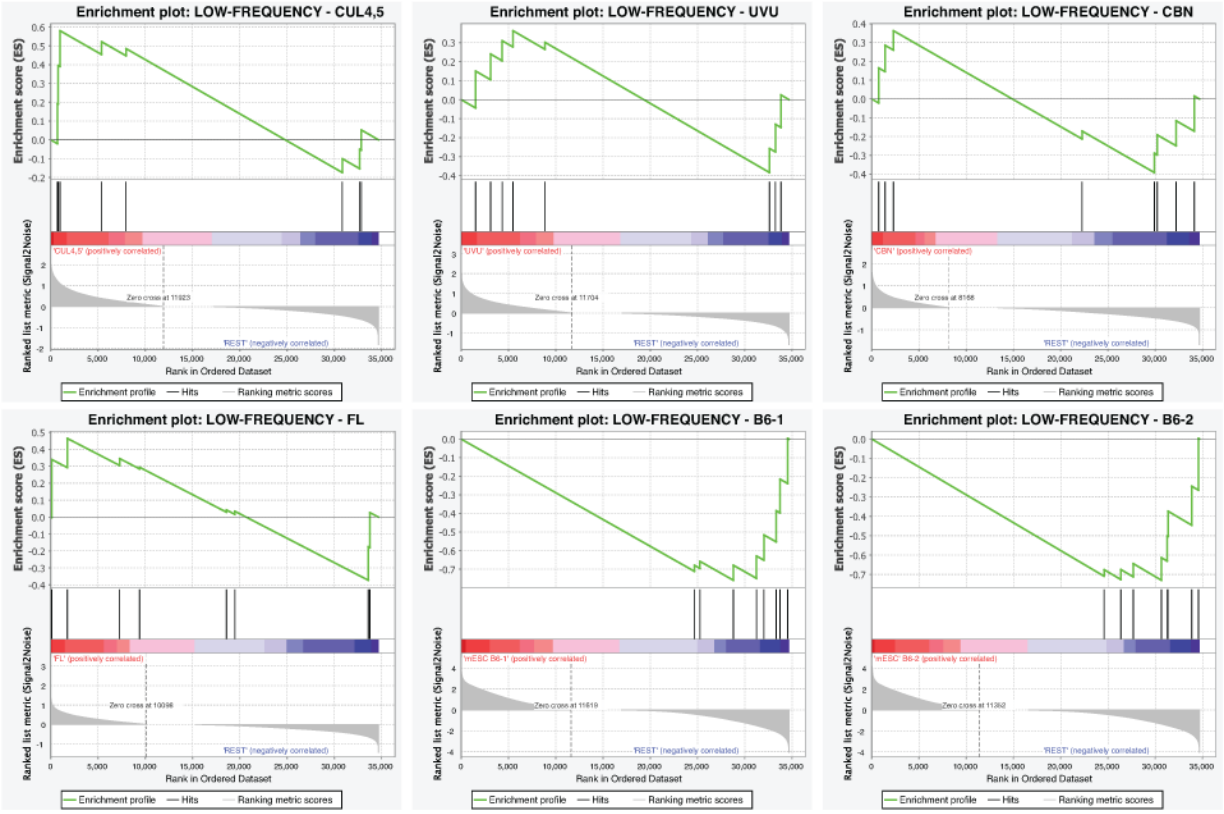
Enrichment plot: LOW-FREQUENCY - CUL4,5
Enrichment plot: LOW-FREQUENCY - UVU
Enrichment plot: LOW-FREQUENCY - CBN
Enrichment plot: LOW-FREQUENCY - FL
Enrichment plot: LOW-FREQUENCY - B6-1
Enrichment plot: LOW-FREQUENCY - B6-2
Enrichment score (ES)
Ranked list metric (Signal2Noise)
Rank in Ordered Dataset
Enrichment profile
Hits
Ranking metric scores

E

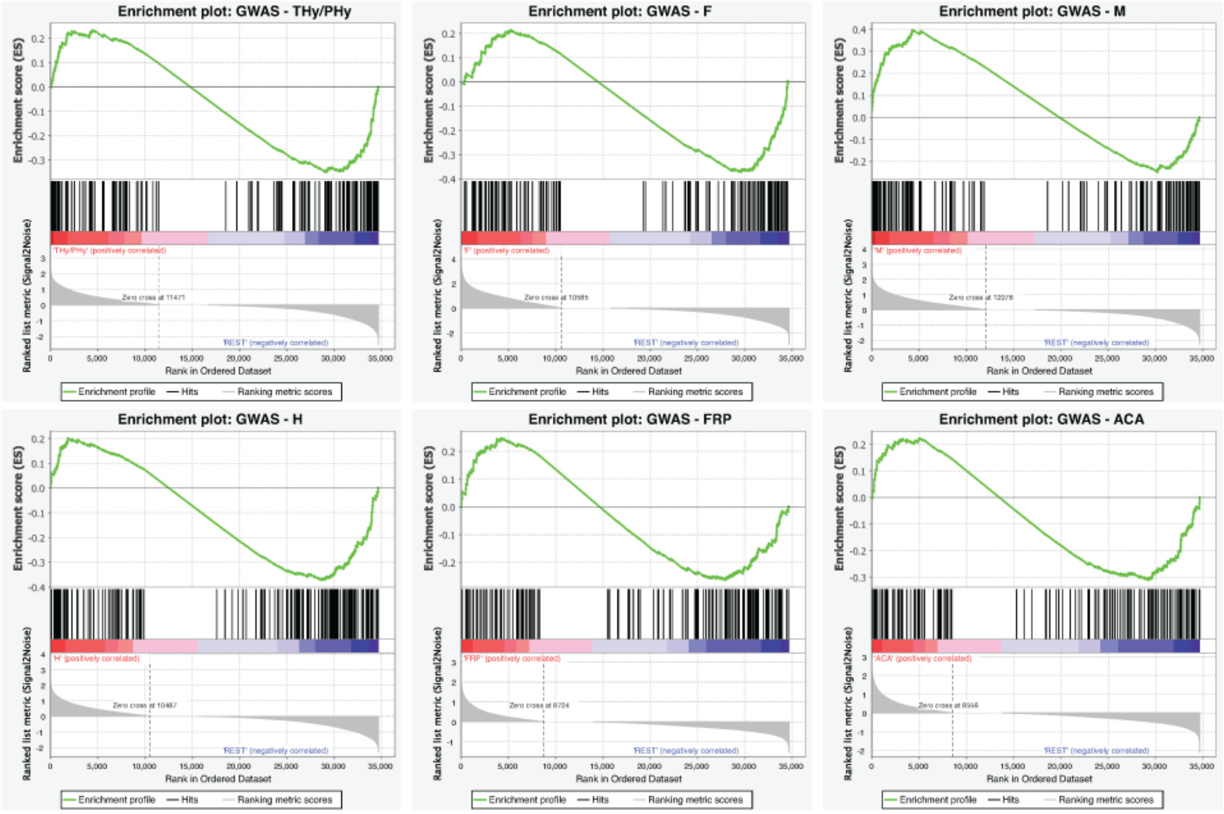
Enrichment plot: GWAS - THy/PHy
Enrichment plot: GWAS - F
Enrichment plot: GWAS - M
Enrichment plot: GWAS - H
Enrichment plot: GWAS - FRP
Enrichment plot: GWAS - ACA
Enrichment score (ES)
Ranked list metric (Signal2Noise)
Rank in Ordered Dataset
Enrichment profile
Hits
Ranking metric scores

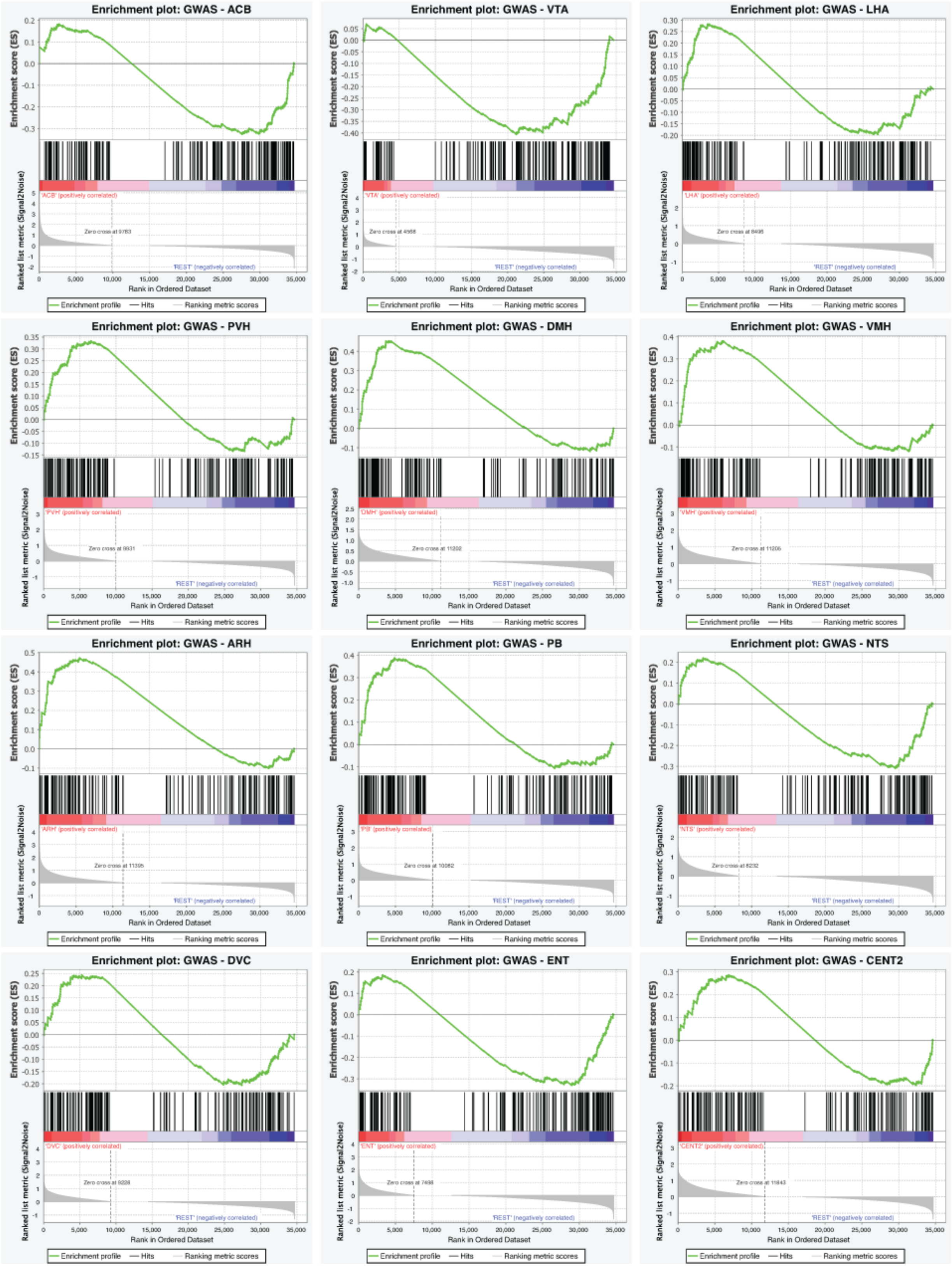
Enrichment plot: GWAS - ACB
Enrichment plot: GWAS - VTA
Enrichment plot: GWAS - LHA
Enrichment plot: GWAS - PVH
Enrichment plot: GWAS - DMH
Enrichment plot: GWAS - VMH
Enrichment plot: GWAS - ARH
Enrichment plot: GWAS - PB
Enrichment plot: GWAS - NTS
Enrichment plot: GWAS - DVC
Enrichment plot: GWAS - ENT
Enrichment plot: GWAS - CENT2
Enrichment score (ES)
Ranked list metric (Signal2Noise)
Rank in Ordered Dataset
'REST' (negatively correlated)
Enrichment profile
Hits
Ranking metric scores

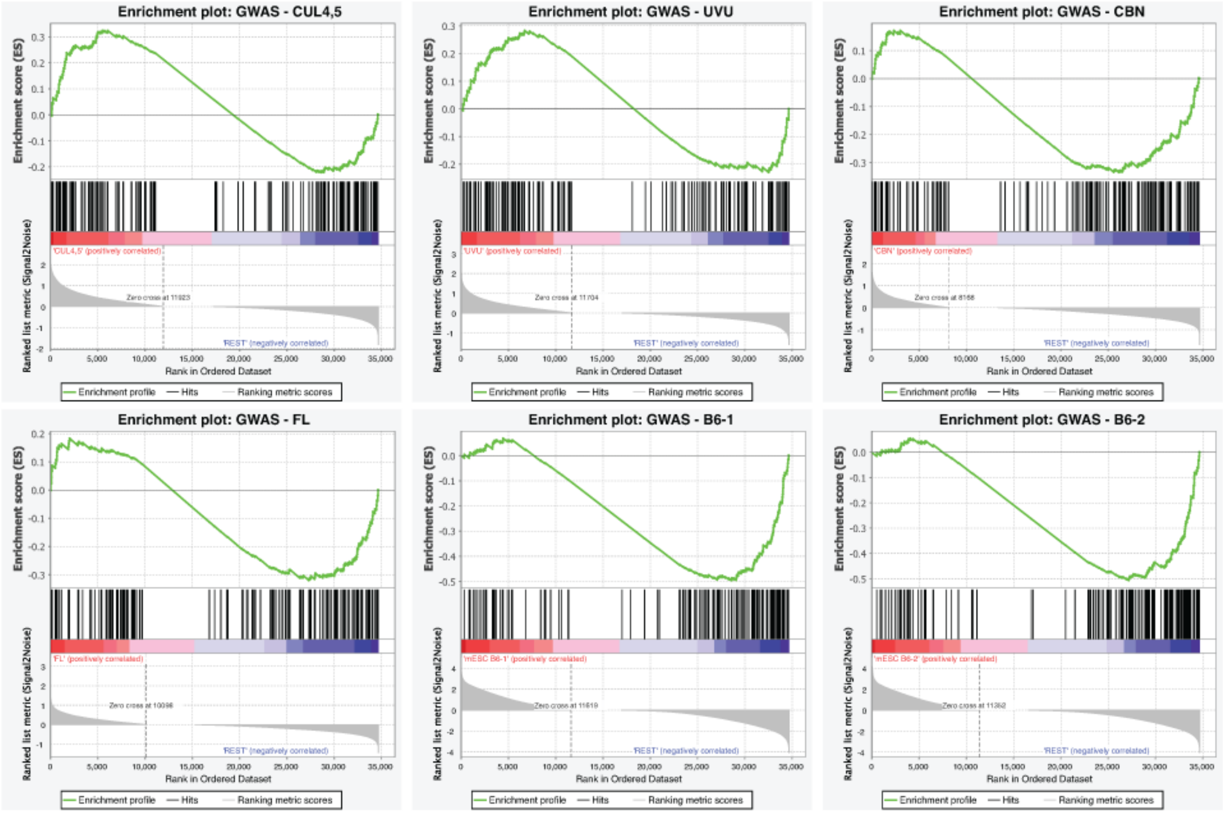
Enrichment plot: GWAS - CUL4,5
Enrichment plot: GWAS - UVU
Enrichment plot: GWAS - CBN
Enrichment plot: GWAS - FL
Enrichment plot: GWAS - B6-1
Enrichment plot: GWAS - B6-2
Enrichment score (ES)
Ranked list metric (Signal2Noise)
Rank in Ordered Dataset
Enrichment profile
Hits
Ranking metric scores

F

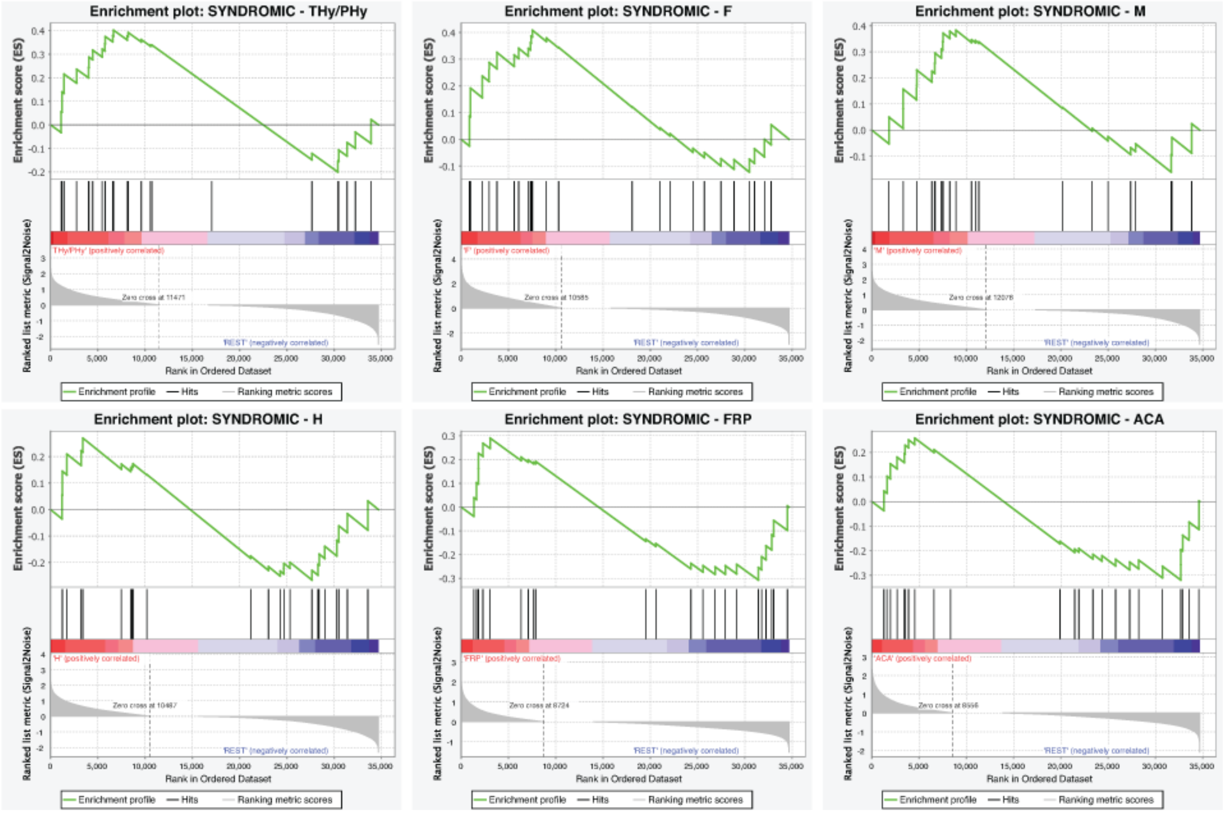
Enrichment plot: SYNDROMIC - THy/PHy
Enrichment plot: SYNDROMIC - F
Enrichment plot: SYNDROMIC - M
Enrichment plot: SYNDROMIC - H
Enrichment plot: SYNDROMIC - FRP
Enrichment plot: SYNDROMIC - ACA
Enrichment score (ES)
Ranked list metric (Signal2Noise)
Rank in Ordered Dataset
Enrichment profile
Hits
Ranking metric scores

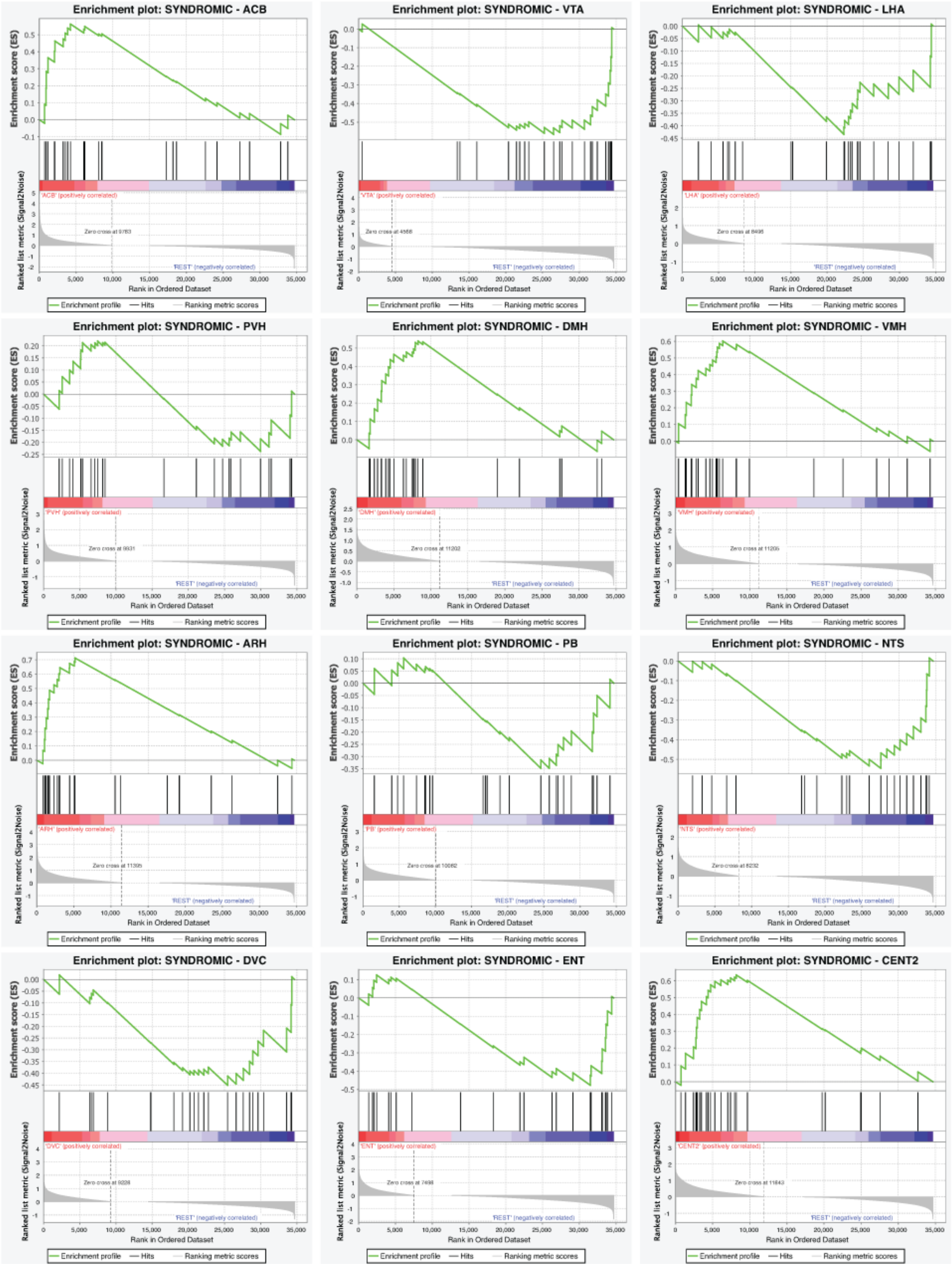

Enrichment plot: SYNDROMIC - ACB
Enrichment plot: SYNDROMIC - VTA
Enrichment plot: SYNDROMIC - LHA
Enrichment plot: SYNDROMIC - PVH
Enrichment plot: SYNDROMIC - DMH
Enrichment plot: SYNDROMIC - VMH
Enrichment plot: SYNDROMIC - ARH
Enrichment plot: SYNDROMIC - PB
Enrichment plot: SYNDROMIC - NTS
Enrichment plot: SYNDROMIC - DVC
Enrichment plot: SYNDROMIC - ENT
Enrichment plot: SYNDROMIC - CENT2
Enrichment score (ES)
Ranked list metric (Signal2Noise)
Rank in Ordered Dataset
Enrichment profile
Hits
Ranking metric scores

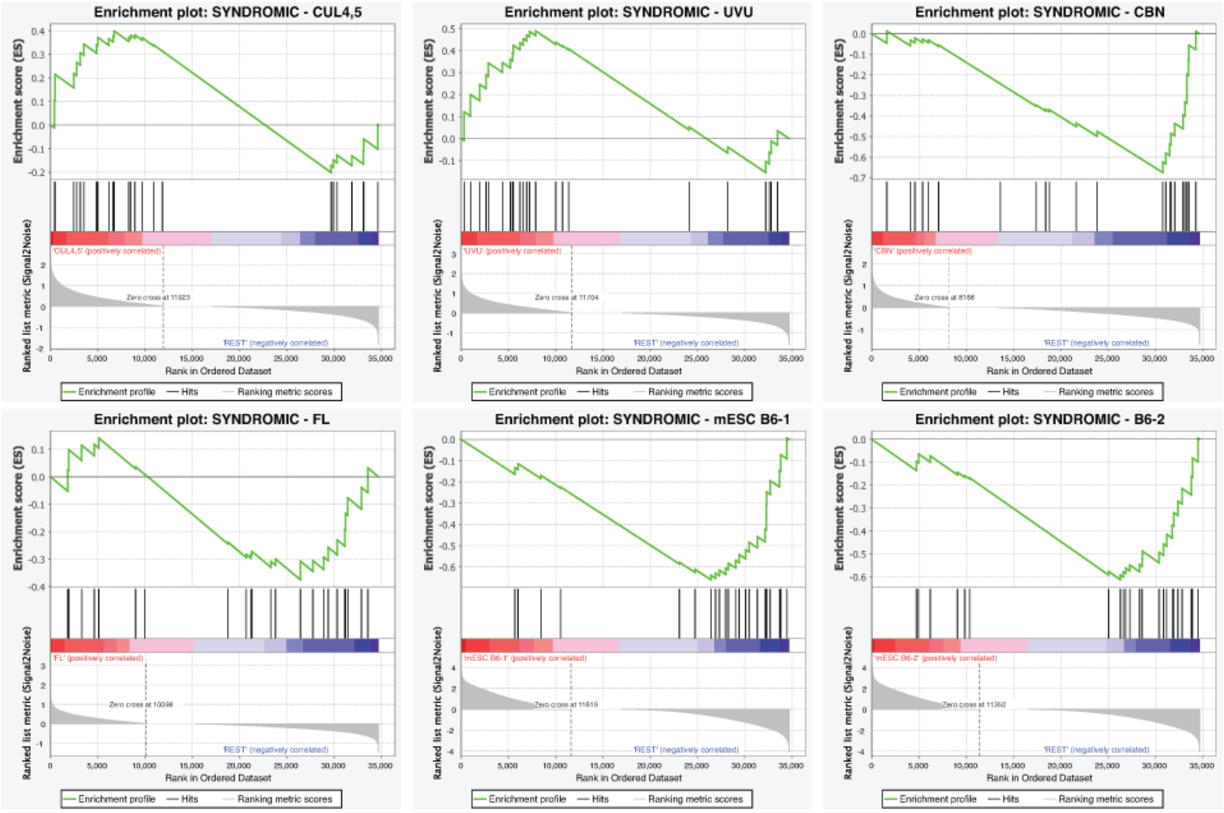

Supplemental Figure 6 | GSEA of obesity-associated genes across the brain regions of energy balance. (A) The enrichment score generated by GSEA was plotted, and regions with a positive enrichment score and a FDR adjusted p-value < 0.05 were denoted with a *. For each region, individual enrichment plots are displayed for genes associated to each genetic category: monogenic obesity **(B)**; rare coding variants **(C)**; low-frequency coding variants **(D)**; GWAS **(E)**; syndromic **(F)**.

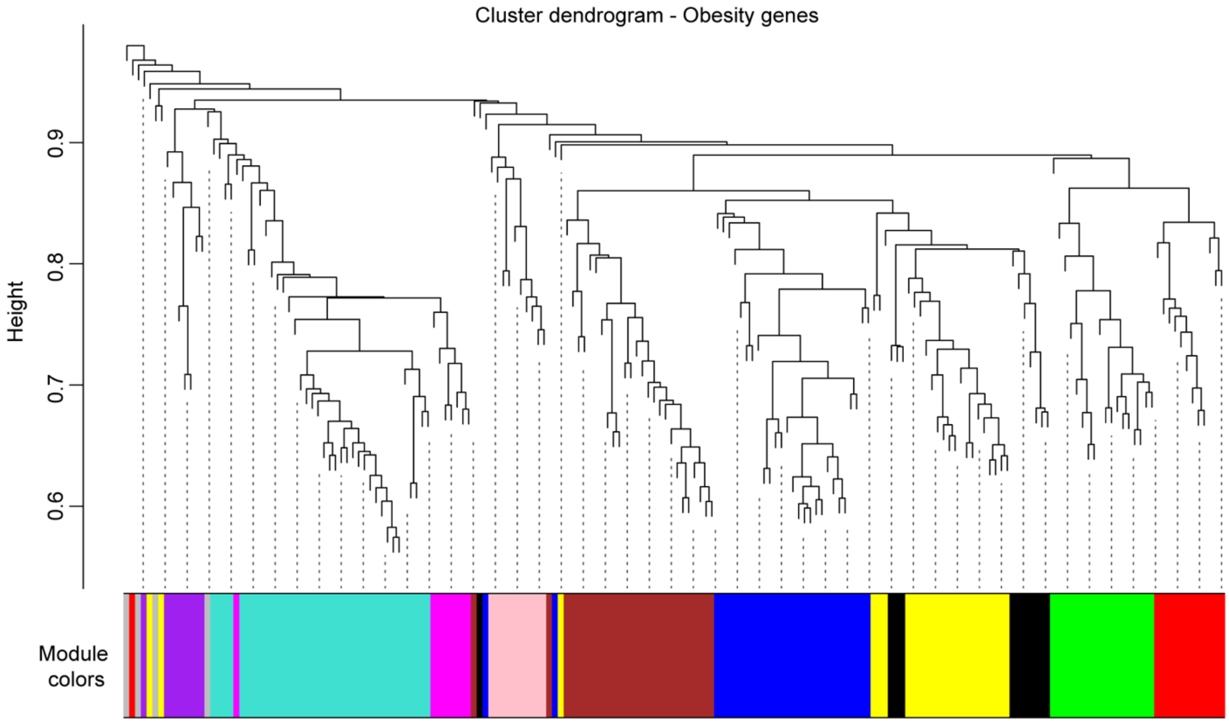

Supplemental Figure 7 | Obesity-associated genes cluster in 11 modules. WGCNA cluster dendrogram. Replicates of the same brain region were averaged, and then a soft thresholding power was chosen to give the highest separation between modules.

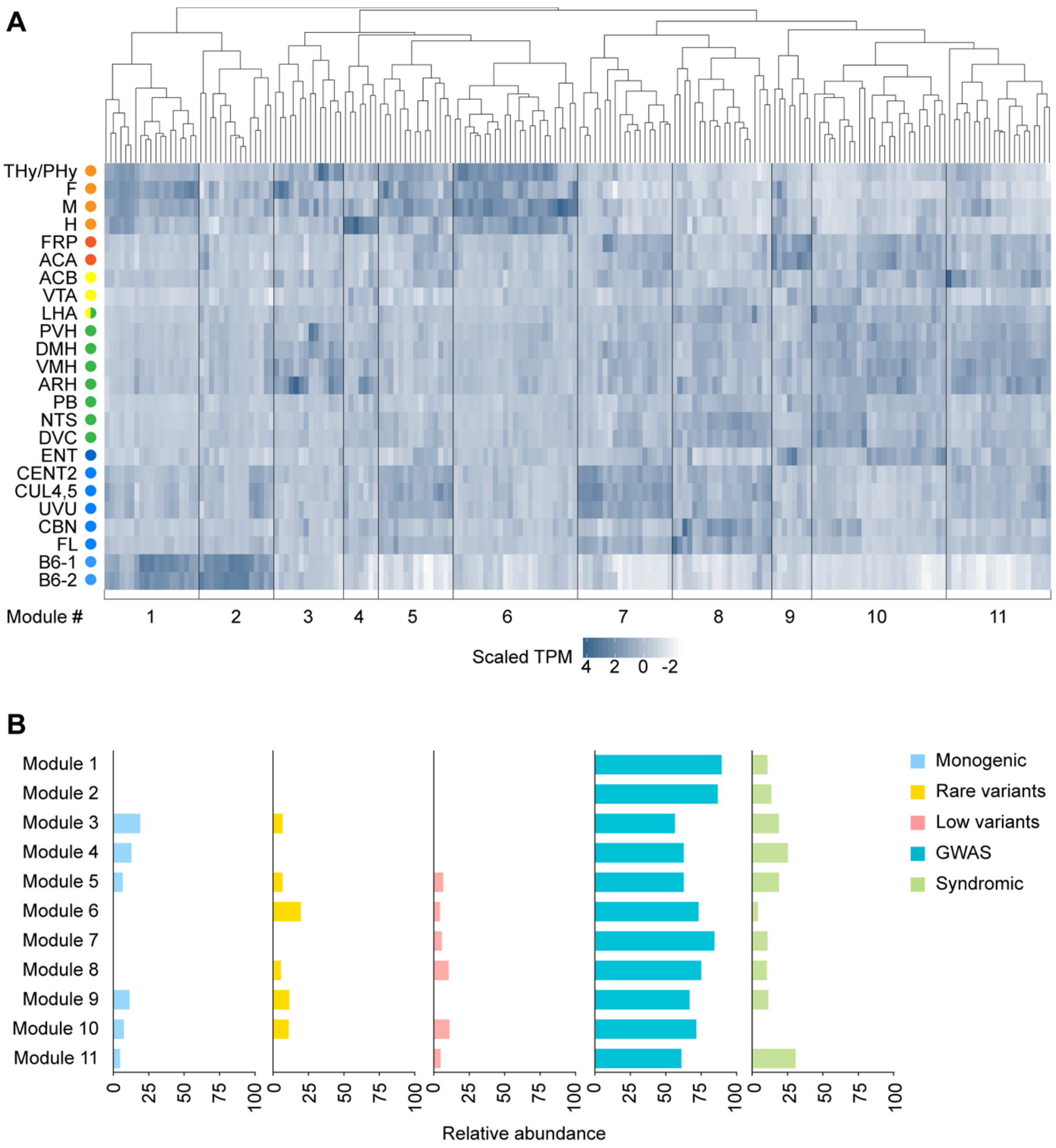

Supplemental Figure 8 | Organization of obesity-associated genes into modules. (A) Unsupervised hierarchical clustering and heat map of obesity-associated genes. TPM values were scaled for each gene to improve visualization. Higher expression is denoted by darker shades of blue. Module 1 shows enrichment of genes in the embryonic regions and mESC lines. Genes in module 2 had higher expression in stem cells. Module 3 featured genes with high expression in embryonic and hypothalamic regions. Module 4 was characterized by genes with high expression in the embryonic hindbrain. In module

5 there was enrichment of genes in regions of the embryonic brain, executive system, reward system and cerebellum. In module 6, expression was highest in the embryonic regions. Genes defining module 7 had high expression in regions of the executive system, reward system and cerebellum. Genes in module 8 showed enrichment in regions of the homeostatic system and cerebellum. Module 9 showed the presence of genes specifically enriched in regions of the executive system. Modules 10 and 11 showed increased expression of genes in regions of the executive and reward system. Distinguishing module 10 and 11, module 10 had increased expression in homeostatic regions and module 11 had increased expression in hypothalamic regions **(B)** Relative abundance (given as percentage) of the genes within the modules, based on the genetic categories. All modules were characterized by the presence of genes belonging to more than one genetic category, with module 5 being defined by genes associated with all five the genetic evidence categories. Except for GWAS genes which were distributed across all modules, genes from the remaining genetic categories were present only in some modules. Genes associated with monogenic obesity were expressed in modules 3, 4, 5, 9, 10, and 11; but primarily in module 3, the module that is enriched for genes in embryonic and hypothalamic regions in panel **A**. Rare coding variant genes were distributed across modules 3, 5, 6, 8, 9 and 10 with predominance in module 6, the module that is enriched for genes in embryonic regions in panel **A**. Low-frequency coding variant genes were identified in modules 5, 6, 7, 8, 10 and 11, with prevalence in module 11, in which there was enrichment in regions of the executive system, reward system and hypothalamus in panel **A**). Syndromic genes were distributed across all modules, except module 10, with predominance in module 11.

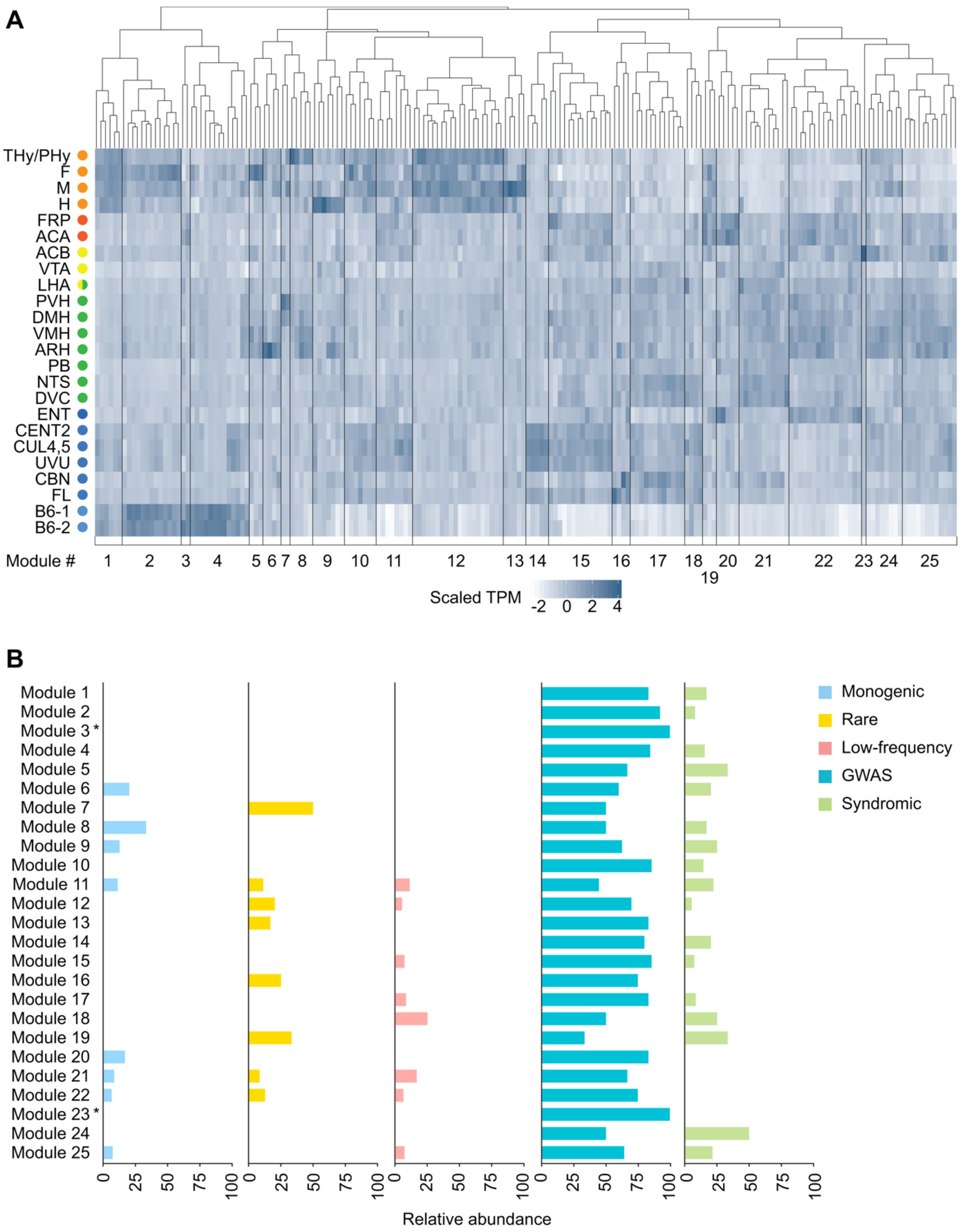

* Module 3, n = 2; Module 23, n = 1

Supplemental Figure 9 | Organization of obesity-associated genes into smaller modules. (A) Unsupervised hierarchical clustering and heat map of obesity-associated genes, and separation into modules based on the hierarchical clustering. For heat map, TPM values were scaled for each gene to improve visualization. Genes were sorted by hierarchical clustering of scaled genes. Scaled TPM value are shown in shades of blue (darker is higher expression). **(B)** Percentage distribution of the genes within the modules, based on the genetic categories.

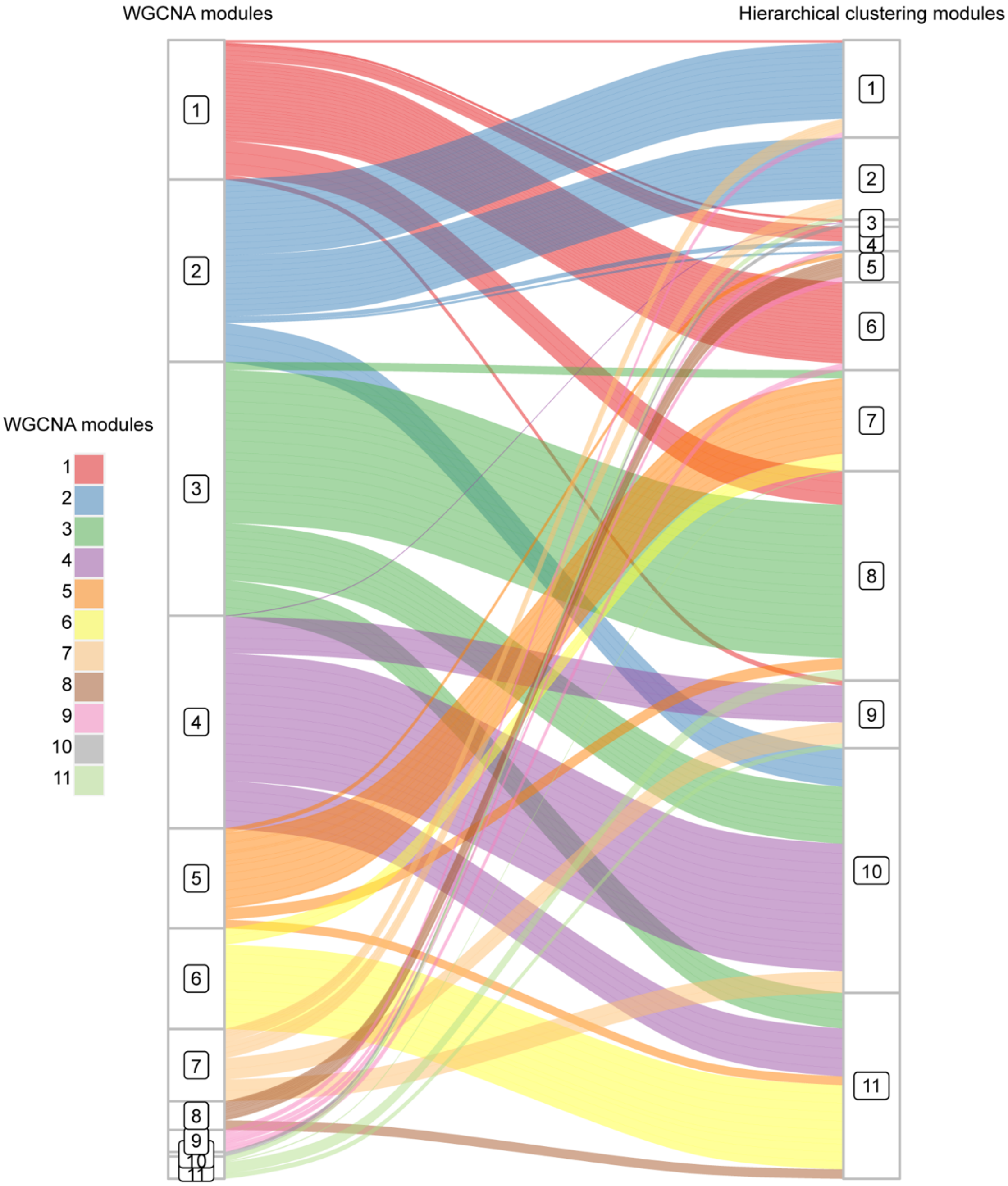

Supplemental Figure 10 | Two independent clustering approaches for obesity-associated genes show a consistent signature. Alluvial diagram was used to visualize the composition of the 11 modules obtained using WGCNA (on the left) as well as unsupervised hierarchical clustering (on the right).

A

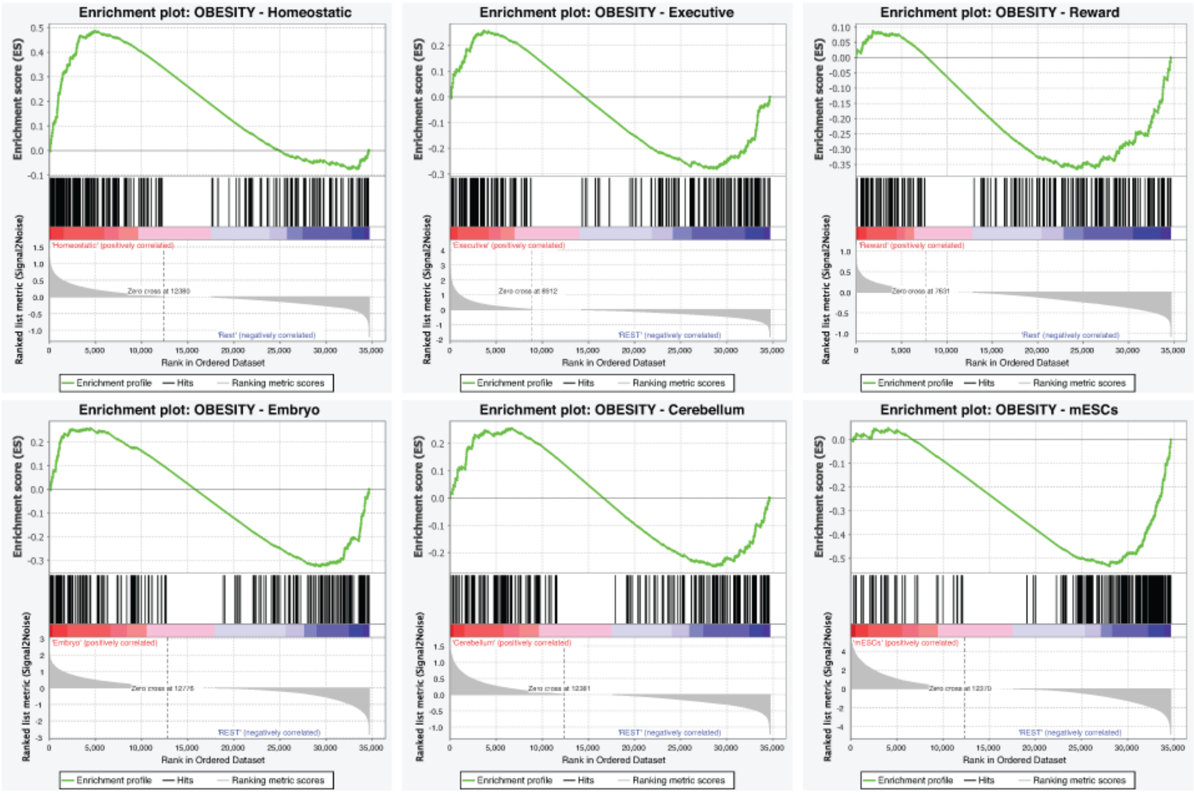
Enrichment plot: OBESITY - Homeostatic
Enrichment plot: OBESITY - Executive
Enrichment plot: OBESITY - Reward
Enrichment plot: OBESITY - Embryo
Enrichment plot: OBESITY - Cerebellum
Enrichment plot: OBESITY - mESCs
Enrichment score (ES)
Ranked list metric (Signal2Noise)
Rank in Ordered Dataset
Enrichment profile
Hits
Ranking metric scores

B

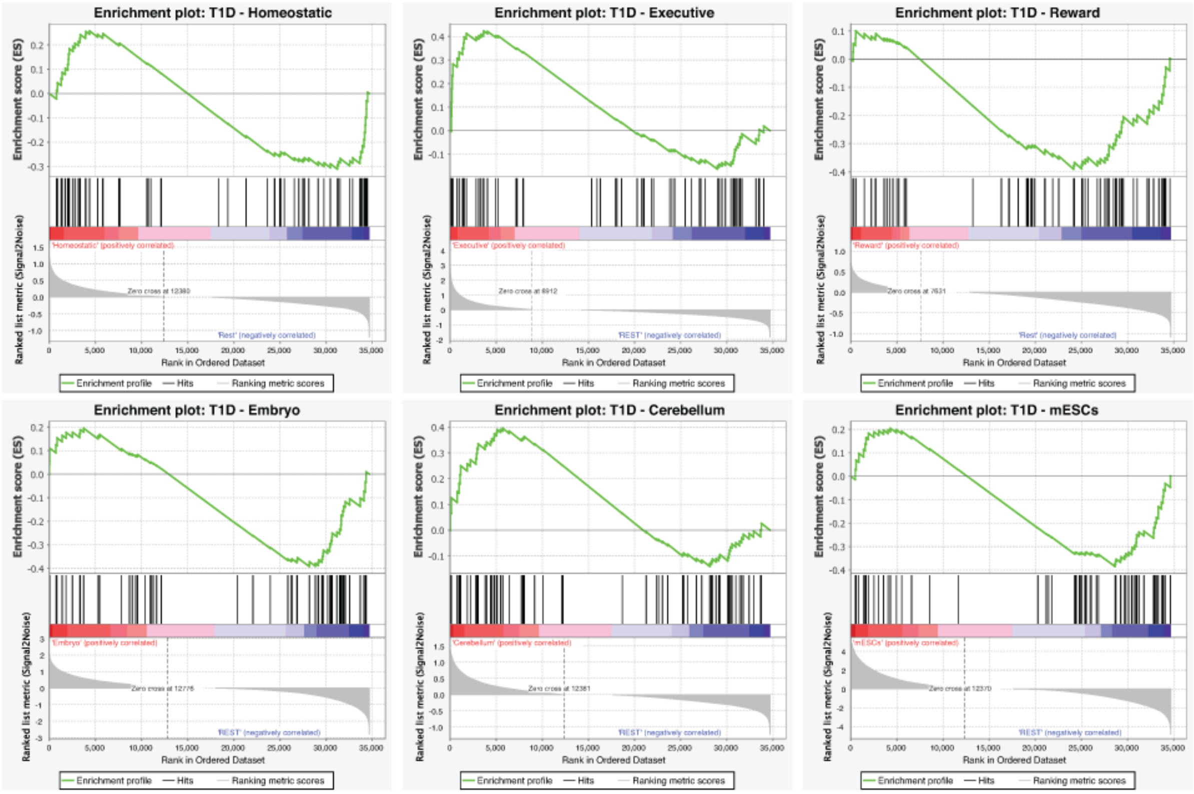
Enrichment plot: T1D - Homeostatic
Enrichment plot: T1D - Executive
Enrichment plot: T1D - Reward
Enrichment plot: T1D - Embryo
Enrichment plot: T1D - Cerebellum
Enrichment plot: T1D - mESCs
Enrichment score (ES)
Ranked list metric (Signal2Noise)
Rank in Ordered Dataset
Enrichment profile
Hits
Ranking metric scores

C

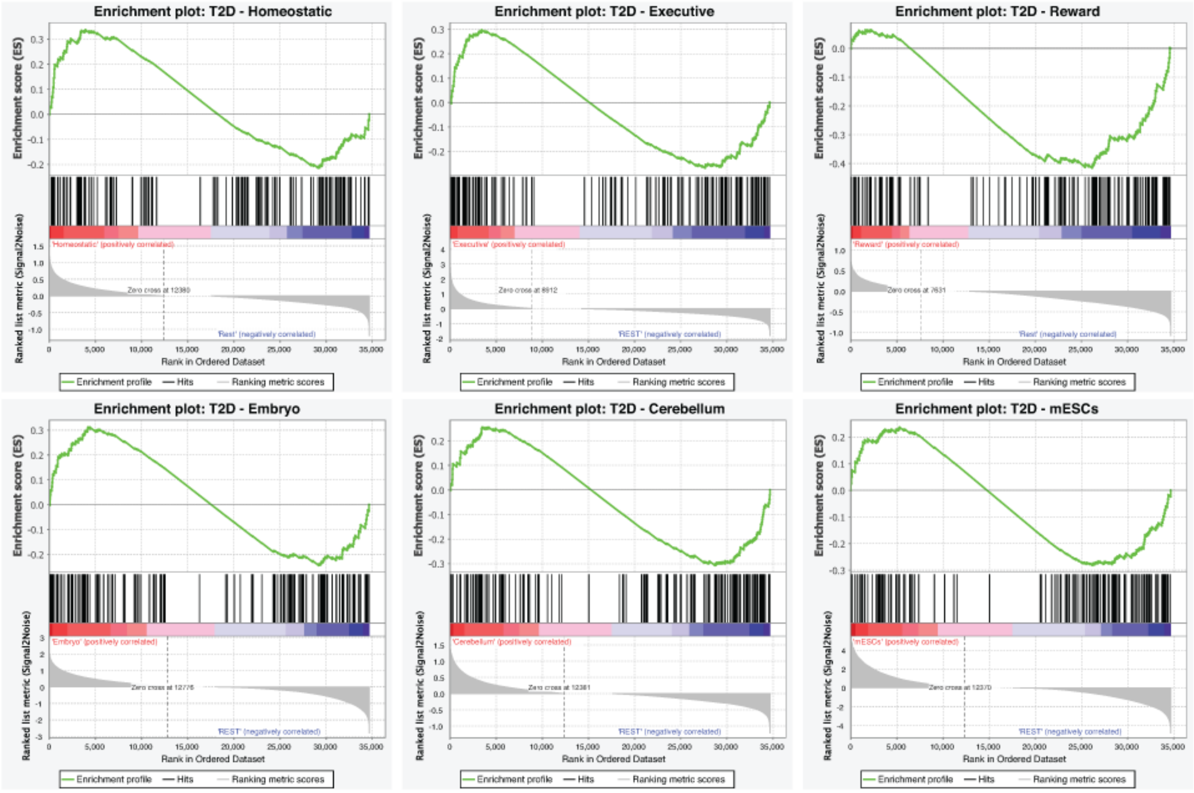

Enrichment plot: T2D - Homeostatic
Enrichment plot: T2D - Executive
Enrichment plot: T2D - Reward
Enrichment plot: T2D - Embryo
Enrichment plot: T2D - Cerebellum
Enrichment plot: T2D - mESCs
Enrichment score (ES)
Ranked list metric (Signal2Noise)
Rank in Ordered Dataset
Enrichment profile
Hits
Ranking metric scores

D

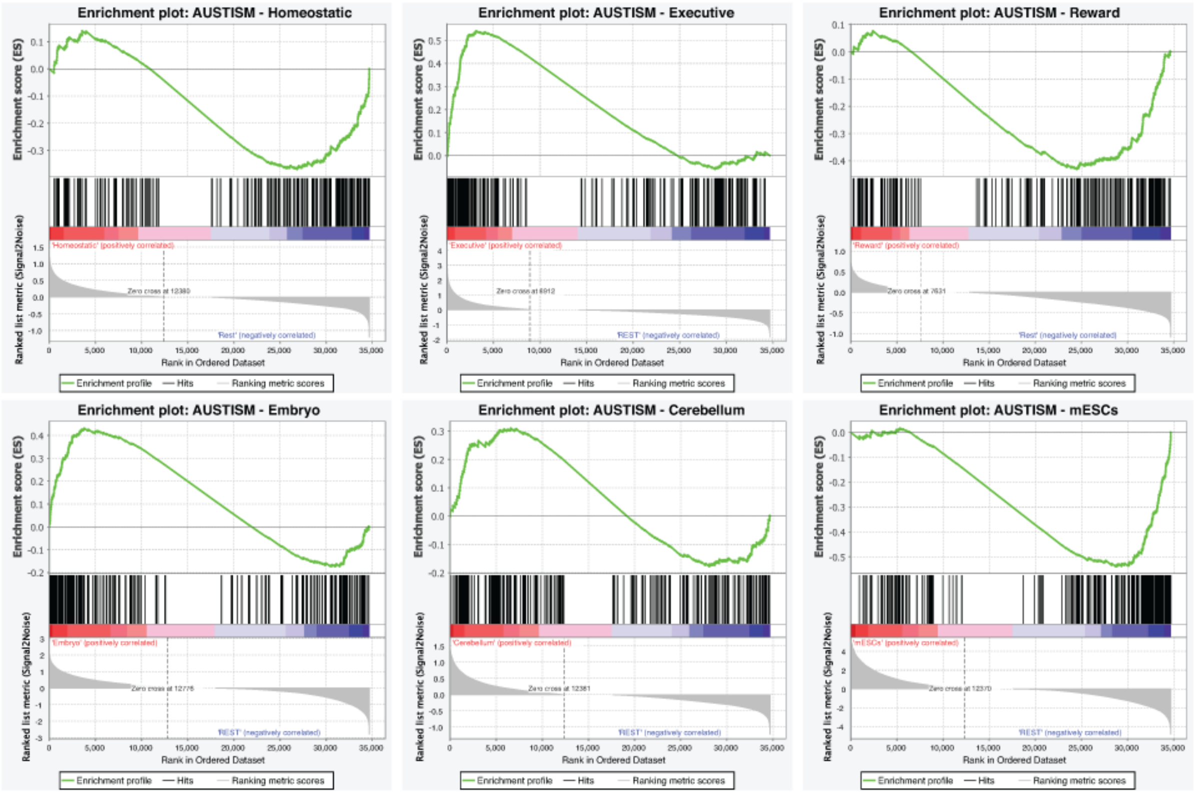

Enrichment plot: AUSTISM - Homeostatic
Enrichment plot: AUSTISM - Executive
Enrichment plot: AUSTISM - Reward
Enrichment plot: AUSTISM - Embryo
Enrichment plot: AUSTISM - Cerebellum
Enrichment plot: AUSTISM - mESCs
Enrichment score (ES)
Ranked list metric (Signal2Noise)
Rank in Ordered Dataset
Enrichment profile
Hits
Ranking metric scores

E

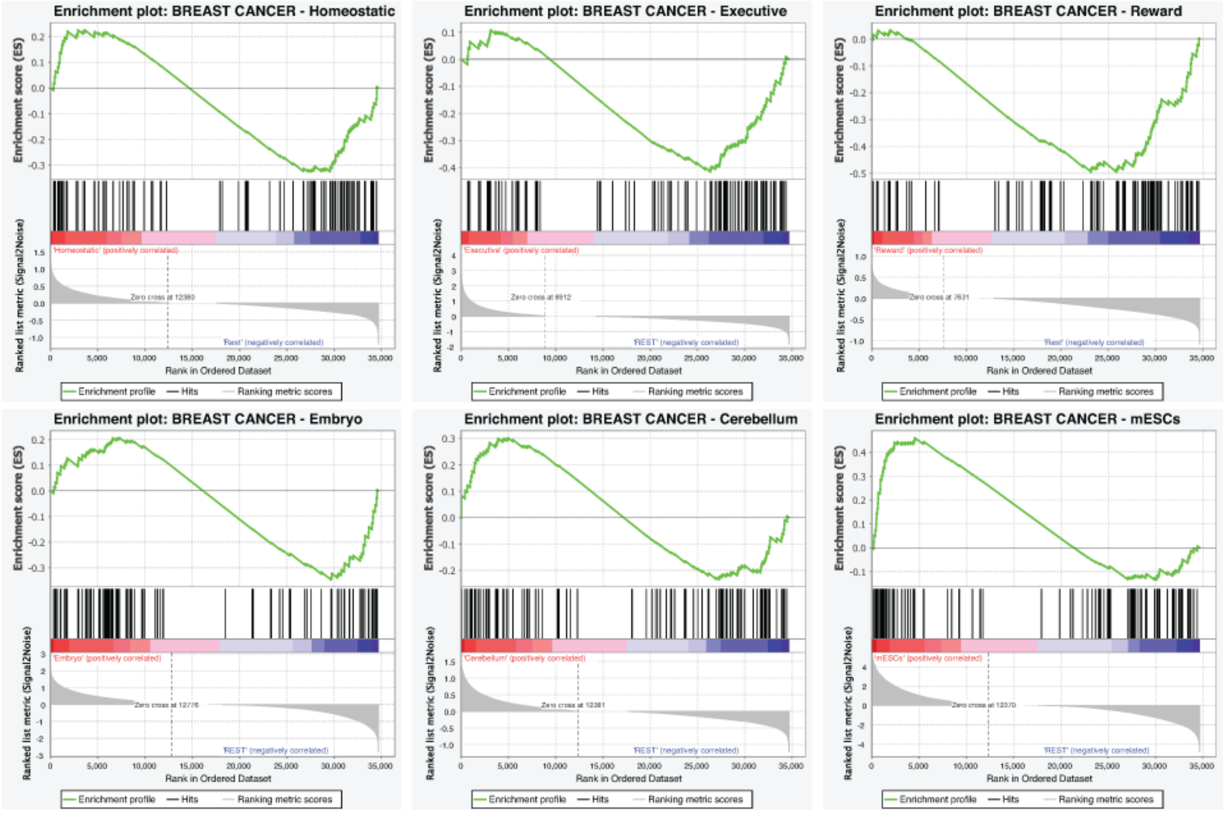

Supplemental Figure 11 | GSEA of obesity-, T1D-, T2D-, autism- and breast cancer-associated genes across the brain regions of energy balance. For each system (homeostatic, executive, reward, embryonic brain, cerebellum, mESC) samples from all the regions were aggregated, and individual enrichment plots are displayed for genes associated to each disease: obesity **(A)**; T1D **(B)**; T2D **(C)**; autism **(D)**; breast cancer **(E)**.

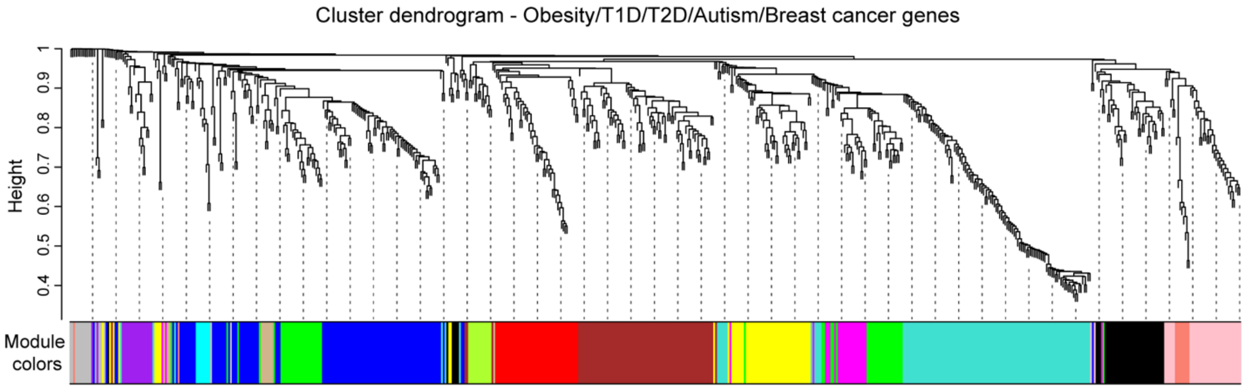

Supplemental Figure 12 | Obesity-, T2D-, T1D-, autism-, breast cancer-associated genes cluster in 15 modules. WGCNA cluster dendrogram. Replicates of the same brain region were averaged, and then a soft thresholding power was chosen to give the highest separation between modules.

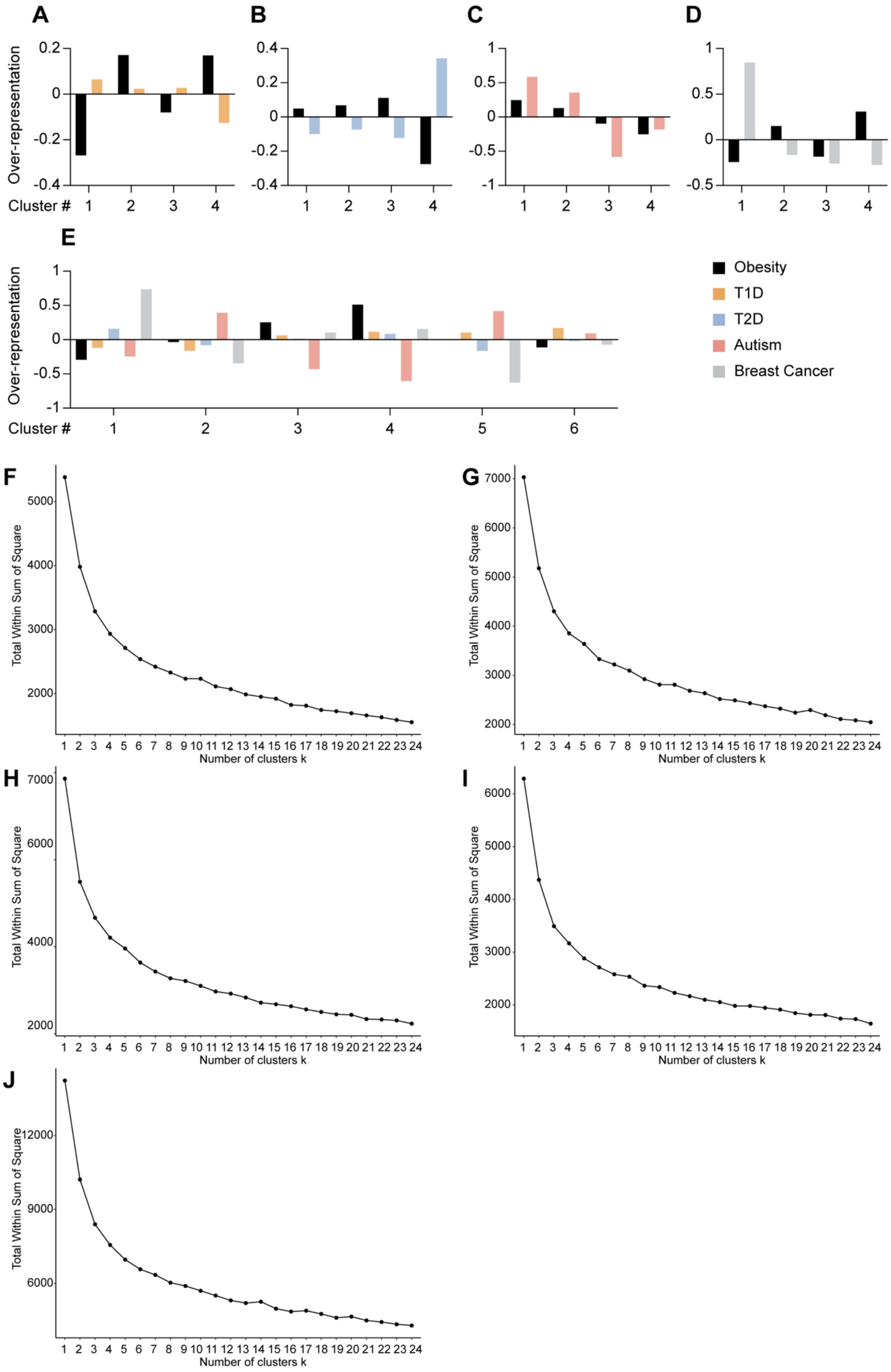
A
B
C
D
E
Over-representation
Cluster #
Obesity
T1D
T2D
Autism
Breast Cancer
F
G
H
I
J
Total Within Sum of Square
Number of clusters k

Supplemental Figure 13 | Figure 4 | K-means clustering of obesity-associated genes with genes associated with other diseases. The localization of expression of the obesity genes and the genes associated with one or more other diseases were clustered into k clusters in which each observation belongs to the cluster with the nearest mean. Enrichment was calculated as over-representation score which indicates the deviation from an equal distribution across modules, normalizing for module size and gene list size, and was plotted to show a positive deflection where a gene list is over-represented. **(A)** Four clusters of T1D- and obesity-associated genes were chosen based on the elbow plot in **F**. Cluster 1 contained predominantly T1D-associated genes, on the other hand, clusters 2 and 4 were mainly contributed by obesity-associated genes. **(B)** Four clusters of T2D- and obesity-associated genes were chosen based on the elbow plot in **G**. Clusters 1, 2 and 3 are mostly defined by obesity-associated genes, the main contribution to cluster 4 are T2D-associated genes. **(C)** Four clusters of autism- and obesity-associated genes were chosen based on the elbow plot in **H**. Clusters 1 and 2 are predominantly defined by autism associated genes. In clusters 3 and 4 the contribution is mixed between autism and obesity. **(D)** Four clusters of breast cancer- and obesity-associated genes were chosen based on the elbow plot in **I**. Cluster 1 consist mainly of breast cancer-associated genes, both clusters 2 and 4 showed primary contribution by obesity-associated genes, and only cluster 3 has a more equal contribution between the diseases**. (E)** Six clusters formed from all genes across all five diseases together were chosen based on the elbow plot in **J**. Cluster 1 is mainly contributed by breast cancer-associated genes; clusters 2 and 5 are mainly defined by autism-associated genes; obesity-associated genes are enriched in clusters 3 and 4; cluster 6 is predominantly contributed by T1D-associated genes. was plotted to show a positive deflection where a gene list is over-represented.

Supplemental Table 1 | Abbreviations for brain regions and mouse stem cells.

Acronyms for brain regions were taken from the Allen Brain Atlas.

Color Key	Abbreviation	Region/Cell Line Name
	THy, PHy	Terminal (rostral) and peduncular (caudal) hypothalamus
	F	Forebrain
	M	Midbrain
	H	Hindbrain
	FRP	Frontal pole cortex
	ACA	Anterior cingulate area
	ACB	Nucleus accumbens
	VTA	Ventral tegmental area
	LHA	Lateral hypothalamic area
	PVH	Paraventricular hypothalamic nucleus
	DMH	Dorsomedial nucleus of the hypothalamus
	VMH	Ventromedial hypothalamic nucleus
	ARH	Arcuate hypothalamic nucleus
	PB	Parabrachial nucleus
	NTS	Nucleus of the solitary tract
	DVC	Dorsal-vagal complex
	ENT	Entorhinal area
	CENT2	Central lobule II
	CUL4,5	Culmen lobules IV-V

Supplemental Table 1 | Cont.

Color Key	Abbreviation	Region/Cell Line Name
●	UVU	Uvula (IX)
●	CBN	Cerebellar nuclei
●	FL	Flocculus
●	B6-1	C57BL/6 mouse embryonic stem cells, clone 1
●	B6-2	C57BL/6 mouse embryonic stem cells, clone 2

Supplemental Table 2 | Samples overview. Color-keyed description of each brain sample including: region, sample batch, number of mice, number of sections per sample, number of mice and type of punch.

Color Key	Region	Batch #	N Mice	N Sections	Age	Punch
● (orange)	THy, PHy	6	4	4	E14.5	Single
		7	4	4		
		8	4	4		
● (orange)	F	8	4	4	E14.5	Single
● (orange)	M	6	4	4	E14.5	Single
		8	4	4		
● (orange)	H	6	4	4	E14.5	Single
		7	4	4		
		8	4	4		
● (red-orange)	FRP	2	3	1	P56	Bilateral
		3	3	1		
		4	3	1		
● (red-orange)	ACA	1	3	2	P56	Bilateral
		3	3	2		
		4	3	2		
● (yellow)	ACB	2	1	2	P56	Bilateral
		3	3	2		
		4	3	2		

Supplemental Table 2 | Cont.

Color Key	Region	Batch #	N Mice	N Sections	Age	Punch
	VTA	1	3	2	P56	Bilateral
		2	3	2		
		3	3	2		
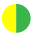	LHA	1	3	3	P56	Bilateral
		2	3	3		
		4	4	3		
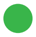	PVH	1	3	2	P56	Bilateral
		4	3	2		
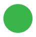	DMH	1	3	2	P56	Bilateral
		2	3	2		
		3	3	2		
		4	3	2		
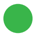	VMH	2	3	1	P56	Bilateral
		3	3	1		
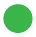	ARH	2	3	2	P56	Bilateral
		3	3	2		
		4	3	2		
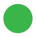	PB	1	3	2	P56	Bilateral
		2	3	2		
		4	3	2		

Supplemental Table 2 | Cont.

Color Key	Region	Batch #	N Mice	N Sections	Age	Punch
	NTS	1	3	2	P56	Bilateral
		2	3	2		
		3	3	2		
		4	3	3		
	DVC	2	3	1	P56	Bilateral
		3	3	1		
		4	3	1		
	ENT	2	3	2	P56	Bilateral
		3	3	2		
		4	3	2		
	CENT2	1	3	1	P56	Single
		2	3	1		
		3	3	1		
		4	3	1		
	CUL4,5	2	3	2	P56	Single
		3	3	2		
		4	3	2		
		5	4	2		
	UVU	1	3	1	P56	Single
		2	3	1		

Supplemental Table 2 | Cont.

Color Key	Region	Batch #	N Mice	N Sections	Age	Punch
●	UVU	5	4	1	P56	Single
●	CBN	1	3	1	P56	Bilateral
		3	3	1		
		4	3	1		
●	FL	1	3	1	P56	Single
		2	3	1		
		3	3	1		
		4	3	1		

Supplemental Table 3 | Samples overview cont. Color-keyed description of mouse embryonic stem cells sample including: cell line name, mouse strain, sample batch, number of cells plated for each sample.

Color Key	mESC Line	Batch #	Mouse Strain	N Cells/35 mm Well
●	B6-1	9	C57BL/6	3×10^5
		9	C57BL/6	3×10^5
●	B6-2	9	C57BL/6	3×10^5
		9	C57BL/6	3×10^5

Supplemental Table 4 | Categories of obesity-associated genes.

Genetic Category	N Genes
Monogenic	9
Rare coding variants	12
Low-frequency coding variants	9
GWAS	144
Syndromic	25

Supplemental Table 5 | Obesity-associated genes. Description of each gene including: module number based on hierarchical clustering, gene name and genetic category. Relates to Supplemental Figure 8. (B) biological candidate; (C) copy number variation; (D) depict analyses; (G) GRAIL results; (M) BMI-associated variant is in strong LD (r 2 ≥ 0.75) with a missense variant in the indicated gene; (N) gene nearest to index SNP; (Q) association and eQTL data converge to affect gene expression.

Module #	Gene Name	Genetic Category
1	Hif1an	GWAS (N)
1	Cep295	GWAS
1	Zbtb10	GWAS (N)
1	Pbrm1	GWAS (N)
1	Creb1	GWAS (B, N)
1	Phip	Syndromic
1	Mks1	Syndromic
1	Ino80e	GWAS (N)
1	Fancl	GWAS (N)
1	Rpl27a	GWAS (N)
1	Cnot9	GWAS (Q)
1	Nup54	GWAS (M)
1	Sfxn2	GWAS (Q)
1	Kat8	GWAS (N)
1	Usp37	GWAS (N)
1	Gtf3a	GWAS (Q)
1	Snrpd2	GWAS (M)
1	Ttll4	GWAS (M, Q)
1	Zfp646	GWAS (M, Q)
2	Apobr	GWAS (B)
2	Etv5	GWAS (N)
2	Rcc1l	GWAS
2	Tomm40	GWAS (N)
2	Polk	GWAS
2	Adpgk	GWAS (N)
2	Gdf15	GWAS (B)
2	Zfp64	GWAS (N)
2	Hmga1	GWAS (B)
2	Mtfr2	GWAS
2	Cdkal1	GWAS (N)
2	Alms1	Syndromic
2	Mtch2	GWAS (N)
2	Nlrc3	GWAS (N)
2	Rab23	Syndromic
3	Prkd1	GWAS (N)
3	Bbs12	Syndromic

Supplemental Table 5 | Cont.

Module #	Gene Name	Genetic Category
3	Stk33	GWAS
3	Asb4	GWAS (B, N)
3	Pomc	Monogenic/GWAS (Q, B)
3	Bbs10	Syndromic
3	Galnt10	GWAS (N)
3	Gpr151	Rare
3	Tcf7l2	GWAS (B, N)
3	Sim1	Monogenic
3	Smg6	GWAS (D, N)
3	Calcr	GWAS (B, N)
3	Bbs9	Syndromic
3	Tub	Monogenic/GWAS (B)
4	Skor1	GWAS (M)
4	Lmx1b	GWAS (B, N)
4	Olig3	GWAS (G)
4	Lepr	Monogenic/GWAS (N)
4	Vkorc1	GWAS (Q)
4	Lztfl1	Syndromic
4	Rpgrip1l	Syndromic
5	Pex2	GWAS
5	Ift27	Syndromic
5	Txndc12	GWAS
5	Fam57b	GWAS (D)
5	Irs1	GWAS (B, Q)
5	Hip1	GWAS (B, N)
5	Sbk1	GWAS (D, N)
5	Sdccag8	Syndromic
5	Sh2b1	Monogenic/GWAS (N)
5	Map2k5	GWAS (N)
5	Nudt3	GWAS (N)
5	Sema3a	Rare
5	Zfp169	Low-frequency
5	Bbip1	Syndromic
5	Poc5	GWAS (M)
6	Hmgcr	GWAS (N, B)
6	Plxna1	Rare
6	Stk36	GWAS (B, M)
6	Plxna3	Rare
6	Ptbp2	GWAS (N)
6	Zfp608	GWAS (N)
6	Fbxl19	GWAS (D)

Supplemental Table 5 | Cont.

Module #	Gene Name	Genetic Category
6	Kctd15	GWAS (N)
6	Ube2e3	GWAS (N)
6	Zfp668	GWAS (Q)
6	Elavl4	GWAS (B, D, N, Q)
6	Klf7	GWAS (B)
6	Slc39a8	GWAS (Q, M)
6	Ift74	Syndromic
6	Nrp2	Rare
6	Ehbp1	GWAS (B, N)
6	Nrp1	Rare
6	Nav1	GWAS (N)
6	Fign	GWAS (N)
6	Zfhx3	Low-frequency
6	Pgpep1	GWAS (Q, N)
6	Tal1	GWAS (N)
6	Tfap2b	GWAS (N)
6	Gipr	Rare/GWAS (B, M)
6	Lmo1	GWAS
7	Inpp5e	Syndromic
7	Cbln1	GWAS (N)
7	Kcnk3	GWAS (D, N)
7	Foxo3	GWAS (B, N)
7	Zfp142	GWAS (M)
7	Cyp17a1	GWAS (B)
7	Rasa2	GWAS (N)
7	Mtif3	GWAS (N)
7	Efr3b	GWAS (M)
7	Ric3	GWAS (N)
7	Scg3	GWAS (B, D)
7	Stx1b	GWAS (D)
7	Ypel3	GWAS (D)
7	Negr1	GWAS (N)
7	Arl6	Syndromic
7	Ksr2	Low-frequency
7	Nrxn3	GWAS (N)
7	Dmxl2	GWAS (M, N)
7	Frrs1l	GWAS (D)
8	Hhip	GWAS (B, N)
8	Tlr4	GWAS (B, N)
8	Sema3b	Rare
8	Tmem160	GWAS (N, Q)

Supplemental Table 5 | Cont.

Module #	Gene Name	Genetic Category
8	Cep290	Syndromic
8	Gnpda2	GWAS (N)
8	Rab21	Low-frequency
8	Hsd17b12	GWAS (B, M, N)
8	Gbe1	GWAS (B, M, N)
8	Scarb2	GWAS (Q, N)
8	Cobll1	GWAS (N)
8	Cyp27a1	GWAS (B)
8	Pcdh9	GWAS (D)
8	Plcd4	GWAS (B, Q)
8	Gprc5b	GWAS (N)
8	Ssfa2	GWAS
8	Ankdd1b	GWAS
8	Iars2	GWAS
8	Mkks	Syndromic
8	Zbtb7b	Low-frequency
9	Sec16b	GWAS (N)
9	Plxna2	Rare
9	Wdpcp	Syndromic
9	Grp	GWAS (B, G, N)
9	Tnni3k	GWAS (N)
9	Bdnf	Monogenic/GWAS (N)
9	Cadm2	GWAS (N)
9	Stxbp6	GWAS (N)
10	Sema3d	Rare
10	Kcnk9	GWAS (N)
10	Faim2	GWAS (N)
10	Col4a3bp	GWAS (N)
10	Dnajc27	GWAS (M)
10	Rapgef3	Low-frequency
10	Trim66	GWAS (D, M, N)
10	Ache	Low-frequency
10	Rit2	GWAS (B, D)
10	Fhit	GWAS (N)
10	Mc4r	Monogenic/GWAS (N)
10	Cadm1	GWAS (N)
10	Slc22a3	GWAS (N)
10	Lingo2	GWAS (D, N)
10	Lrfn2	GWAS (D)
10	Agbl4	GWAS (N)
10	Doc2a	GWAS (D)

Supplemental Table 5 | Cont.

Module #	Gene Name	Genetic Category
10	Pcsk1	Monogenic
10	Entpd6	Low-frequency
10	Adcy3	GWAS (Q, M)
10	Grid1	GWAS (B, N)
10	Sema3c	Rare
10	Plxna4	Rare
10	Rabep1	GWAS (N)
10	Erbb4	GWAS (D, N)
10	Kctd13	GWAS (D)
10	Mapk3	GWAS (D)
11	Rarb	GWAS (B)
11	Bbs1	Syndromic
11	Bbs7	Syndromic
11	Bbs2	Syndromic
11	Ttc8	Syndromic
11	Qpctl	GWAS (N)
11	Taok2	GWAS (D)
11	Fto	GWAS (N)
11	Nt5c2	GWAS (N)
11	Ralyl	GWAS (D, N)
11	Trim32	Syndromic
11	Zfr2	Low-frequency
11	Ifngr1	GWAS (N)
11	Bbs4	GWAS (B, M, Q)/Syndromic
11	Ntrk2	Monogenic/GWAS (N)
11	Lrp1b	GWAS (N)
11	Park2	GWAS (B, D, N)
11	Bbs5	Syndromic
11	Ets2	GWAS (N)
11	Epb41l4b	GWAS (N)
11	Tmem18	GWAS (N)

Supplemental Table 6 | Expression levels of obesity-associated genes across the brain regions of energy balance. TPM values for each obesity-associate gene were reported for all brain regions and mouse embryonic stem cell samples. For each region TPM value from each replicate were aggregated to calculate the mean and SD.

Supplemental Table 7 | Frequency distribution of obesity-associated genes within each genetic category. Table shows one-way ANOVA reporting the Benjamini, Krieger and Yekutieli two step false discovery rate corrected p-value . Relates to Figure 3A.

Region 1	Region 2	Monogenic	Rare	Low-frequency	GWAS	Syndromic
THy/PHy	F	0.0147	0.4035	0.1	0.7353	0.7561
THy/PHy	M	0.0323	0.4339	0.6154	0.0033	0.7437
THy/PHy	H	0.0461	0.6168	0.547	0.4959	0.0772
THy/PHy	FRP	0.1137	0.0598	0.6084	0.5795	0.0902
THy/PHy	ACA	0.1693	0.0163	0.6217	0.4657	0.0863
THy/PHy	ACB	0.025	0.0245	0.8023	0.3327	0.763
THy/PHy	VTA	0.0365	<0.0001	0.942	0.0165	<0.0001
THy/PHy	LHA	0.0814	0.0258	0.236	0.4502	0.0211
THy/PHy	PVH	0.1694	0.0855	0.095	0.8314	0.1408
THy/PHy	DMH	0.3307	0.0603	0.1194	0.5624	0.6845
THy/PHy	VMH	0.2776	0.0218	0.0941	0.7427	0.692
THy/PHy	ARH	0.033	0.0596	0.1546	0.0365	0.372
THy/PHy	PB	0.0474	0.0022	0.6481	0.1239	0.0008
THy/PHy	NTS	0.0597	0.0269	0.5269	0.3187	0.0031
THy/PHy	DVC	0.1086	0.0238	0.2927	0.6727	0.0119
THy/PHy	ENT	0.0735	0.0238	0.6398	0.1994	0.0102
THy/PHy	CENT2	0.0234	0.0005	0.6379	0.7816	0.5535
THy/PHy	CUL4,5	0.0343	0.0027	0.372	0.979	0.8912
THy/PHy	UVU	0.0317	0.0014	0.7538	0.7487	0.8144
THy/PHy	CBN	0.019	0.0619	0.6193	0.1368	0.0026
THy/PHy	FL	0.0214	0.0016	0.6551	0.4507	0.0457
THy/PHy	mESC B61	0.0051	<0.0001	0.0171	0.0084	<0.0001
THy/PHy	mESC B62	0.0039	<0.0001	0.0227	0.005	<0.0001
F	M	0.7596	0.1063	0.0323	0.001	0.5238
F	H	0.6494	0.1822	0.2953	0.3082	0.0379
F	FRP	0.3835	0.2931	0.2559	0.8289	0.0451
F	ACA	0.2802	0.1151	0.248	0.6955	0.0429
F	ACB	0.8387	0.1552	0.0585	0.5283	0.5405
F	VTA	0.7218	0.0015	0.1158	0.0393	<0.0001
F	LHA	0.4781	0.1609	0.005	0.6767	0.0089
F	PVH	0.28	0.3745	0.001	0.9004	0.0747
F	DMH	0.1386	0.2947	0.0015	0.359	0.4735
F	VMH	0.1711	0.1425	0.001	0.5052	0.9318
F	ARH	<0.0001	0.2923	0.0024	0.0151	0.5604
F	PB	0.6409	0.0252	0.0362	0.23	0.0003
F	NTS	0.5706	0.1661	0.0233	0.5099	0.0011
F	DVC	0.396	0.1521	0.0074	0.9328	0.0048
F	ENT	0.508	0.1518	0.2378	0.3446	0.004
F	CENT2	0.8587	0.0082	0.0349	0.9515	0.7779
F	CUL4,5	0.7409	0.0295	0.0116	0.7156	0.862
F	UVU	0.7653	0.0177	0.0506	0.9858	0.9395
F	CBN	0.9235	0.3001	0.0328	0.2502	0.0009
F	FL	0.8872	0.0191	0.037	0.6773	0.0211
F	mESC B61	0.7103	<0.0001	0.453	0.0217	<0.0001

Supplemental Table 7 | Cont.

Region 1	Region 2	Monogenic	Rare	Low-frequency	GWAS	Syndromic
F	mESC B62	0.6439	<0.0001	0.5201	0.0135	<0.0001
M	H	0.8818	0.7776	0.2699	0.0238	0.1495
M	FRP	0.5713	0.008	0.3107	0.0005	0.1712
M	ACA	0.4384	0.0015	0.3198	0.0002	0.1647
M	ACB	0.9184	0.0026	0.801	<0.0001	0.9797
M	VTA	0.9601	<0.0001	0.5653	<0.0001	<0.0001
M	LHA	0.6864	0.0027	0.4937	0.0002	0.0476
M	PVH	0.4382	0.0127	0.2415	0.0016	0.2515
M	DMH	0.2391	0.008	0.29	0.0182	0.9367
M	VMH	0.2871	0.0022	0.2397	0.009	0.4697
M	ARH	<0.0001	0.0079	0.3556	0.3954	0.2228
M	PB	0.8724	0.0001	0.9633	<0.0001	0.0024
M	NTS	0.7939	0.0029	0.8961	<0.0001	0.0083
M	DVC	0.587	0.0025	0.5817	0.0008	0.0285
M	ENT	0.7217	0.0025	0.3323	<0.0001	0.0247
M	CENT2	0.8981	<0.0001	0.9747	0.0013	0.358
M	CUL4,5	0.9804	0.0002	0.6958	0.0036	0.6429
M	UVU	0.9941	<0.0001	0.8502	0.0011	0.5744
M	CBN	0.8337	0.0083	0.9957	<0.0001	0.0072
M	FL	0.8696	<0.0001	0.9556	0.0002	0.0944
M	mESC B61	0.4983	<0.0001	0.0041	<0.0001	<0.0001
M	mESC B62	0.4426	<0.0001	0.0056	<0.0001	<0.0001
H	FRP	0.6763	0.0175	0.9283	0.2168	0.9418
H	ACA	0.531	0.0038	0.9132	0.1584	0.9586
H	ACB	0.8017	0.0061	0.3941	0.0991	0.1425
H	VTA	0.9214	<0.0001	0.5964	0.0021	0.0063
H	LHA	0.7986	0.0065	0.0747	0.151	0.5879
H	PVH	0.5307	0.0268	0.0236	0.3714	0.7679
H	DMH	0.3034	0.0177	0.0314	0.9189	0.1732
H	VMH	0.3595	0.0053	0.0233	0.7242	0.0307
H	ARH	<0.0001	0.0174	0.0435	0.1583	0.0079
H	PB	0.9905	0.0004	0.2903	0.0265	0.1096
H	NTS	0.9104	0.0068	0.2175	0.0934	0.2288
H	DVC	0.6931	0.0059	0.0989	0.2699	0.4517
H	ENT	0.8356	0.0059	0.893	0.0495	0.4192
H	CENT2	0.782	<0.0001	0.2838	0.3379	0.0185
H	CUL4,5	0.9012	0.0005	0.1357	0.5126	0.057
H	UVU	0.8759	0.0002	0.3601	0.3167	0.0454
H	CBN	0.7198	0.0182	0.2722	0.0301	0.2106
H	FL	0.7544	0.0003	0.2947	0.1513	0.8166
H	mESC B61	0.4091	<0.0001	0.0732	0.0009	<0.0001
H	mESC B62	0.3596	<0.0001	0.0919	0.0005	0.0029
FRP	ACA	0.8343	0.5984	0.9848	0.8608	0.9832
FRP	ACB	0.5039	0.7101	0.4458	0.6784	0.1634
FRP	VTA	0.6058	0.0314	0.6602	0.0651	0.005
FRP	LHA	0.8709	0.7247	0.0903	0.8407	0.5387

Supplemental Table 7 | Cont.

Region 1	Region 2	Monogenic	Rare	Low-frequency	GWAS	Syndromic
FRP	PVH	0.834	0.87	0.0296	0.7329	0.8242
FRP	DMH	0.5401	0.9973	0.0391	0.2571	0.1973
FRP	VMH	0.6174	0.6767	0.0293	0.3776	0.0367
FRP	ARH	0.0002	0.9986	0.0536	0.0082	0.0098
FRP	PB	0.685	0.2324	0.3331	0.3249	0.0943
FRP	NTS	0.7603	0.7377	0.2527	0.6578	0.2018
FRP	DVC	0.9817	0.7022	0.1185	0.8952	0.4091
FRP	ENT	0.8336	0.7013	0.9645	0.4659	0.3784
FRP	CENT2	0.4877	0.1083	0.326	0.7819	0.0224
FRP	CUL4,5	0.5881	0.2575	0.1607	0.5616	0.0672
FRP	UVU	0.5663	0.1836	0.4091	0.8151	0.0538
FRP	CBN	0.4379	0.9879	0.3133	0.3503	0.1853
FRP	FL	0.4654	0.1934	0.3379	0.8414	0.7605
FRP	mESC B61	0.2144	0.0011	0.0601	0.0375	<0.0001
FRP	mESC B62	0.183	0.0006	0.076	0.0242	0.0023
ACA	ACB	0.3804	0.8768	0.4572	0.8109	0.1571
ACA	VTA	0.4684	0.1031	0.674	0.095	0.0054
ACA	LHA	0.7102	0.8614	0.094	0.9796	0.5527
ACA	PVH	0.9997	0.49	0.0311	0.6055	0.8078
ACA	DMH	0.6864	0.596	0.0409	0.1907	0.1901
ACA	VMH	0.7715	0.9125	0.0307	0.2902	0.0349
ACA	ARH	0.0005	0.5996	0.0559	0.0048	0.0092
ACA	PB	0.5388	0.5037	0.3426	0.4184	0.0986
ACA	NTS	0.6072	0.8478	0.2607	0.7889	0.2094
ACA	DVC	0.8165	0.8851	0.123	0.7588	0.4211
ACA	ENT	0.6751	0.8862	0.9797	0.5797	0.39
ACA	CENT2	0.3667	0.2793	0.3354	0.6511	0.0212
ACA	CUL4,5	0.453	0.5441	0.1664	0.4498	0.0641
ACA	UVU	0.434	0.421	0.4199	0.6824	0.0513
ACA	CBN	0.3249	0.5879	0.3225	0.4481	0.1924
ACA	FL	0.3479	0.4382	0.3475	0.9803	0.7766
ACA	mESC B61	0.1473	0.0058	0.0575	0.0567	<0.0001
ACA	mESC B62	0.1238	0.0036	0.0729	0.0376	0.0025
ACB	VTA	0.8788	0.0744	0.7466	0.1526	<0.0001
ACB	LHA	0.6128	0.9844	0.3493	0.8308	0.0448
ACB	PVH	0.3802	0.5924	0.1551	0.4498	0.2411
ACB	DMH	0.2009	0.7075	0.1906	0.1217	0.9165
ACB	VMH	0.2435	0.964	0.1538	0.1947	0.4854
ACB	ARH	<0.0001	0.7114	0.2401	0.0022	0.2326
ACB	PB	0.7925	0.4102	0.8367	0.5687	0.0022
ACB	NTS	0.7161	0.9706	0.702	0.9773	0.0077
ACB	DVC	0.5186	0.9916	0.4222	0.5849	0.0267
ACB	ENT	0.6465	0.9905	0.4728	0.7531	0.0232
ACB	CENT2	0.9796	0.2163	0.8255	0.4893	0.3714
ACB	CUL4,5	0.8989	0.4463	0.5203	0.3197	0.6612
ACB	UVU	0.9243	0.3374	0.9496	0.5167	0.5918

Supplemental Table 7 | Cont.

Region 1	Region 2	Monogenic	Rare	Low-frequency	GWAS	Syndromic
ACB	CBN	0.9143	0.6988	0.8052	0.6035	0.0067
ACB	FL	0.9508	0.3524	0.8442	0.8301	0.0896
ACB	mESC B61	0.5654	0.0036	0.0086	0.0957	<0.0001
ACB	mESC B62	0.5057	0.0022	0.0116	0.0658	<0.0001
VTA	LHA	0.7235	0.0713	0.2086	0.1002	0.0281
VTA	PVH	0.4682	0.0207	0.0816	0.0288	0.0025
VTA	DMH	0.2595	0.0312	0.1032	0.0029	<0.0001
VTA	VMH	0.3103	0.082	0.0808	0.0064	<0.0001
VTA	ARH	<0.0001	0.0316	0.1347	<0.0001	<0.0001
VTA	PB	0.912	0.3348	0.5967	0.3894	0.2546
VTA	NTS	0.8327	0.0687	0.4805	0.1609	0.1245
VTA	DVC	0.6218	0.0762	0.2608	0.0481	0.0471
VTA	ENT	0.7595	0.0764	0.6927	0.2644	0.0535
VTA	CENT2	0.8587	0.5817	0.5868	0.0339	<0.0001
VTA	CUL4,5	0.9797	0.3047	0.3344	0.0153	<0.0001
VTA	UVU	0.9541	0.4074	0.6992	0.0377	<0.0001
VTA	CBN	0.7948	0.0303	0.5689	0.3621	0.1368
VTA	FL	0.8304	0.3909	0.6034	0.1	0.0123
VTA	mESC B61	0.4672	0.2533	0.0207	0.8135	0.2329
VTA	mESC B62	0.4135	0.1948	0.0273	0.6823	0.806
LHA	PVH	0.7099	0.606	0.6253	0.5877	0.4027
LHA	DMH	0.4384	0.7222	0.7078	0.1821	0.0572
LHA	VMH	0.5081	0.9483	0.622	0.2787	0.007
LHA	ARH	0.0001	0.726	0.8104	0.0044	0.0014
LHA	PB	0.8078	0.3992	0.4652	0.4333	0.2894
LHA	NTS	0.8866	0.9862	0.5796	0.8087	0.5078
LHA	DVC	0.889	0.9759	0.8937	0.7394	0.833
LHA	ENT	0.9621	0.9749	0.099	0.5973	0.7901
LHA	CENT2	0.5949	0.2091	0.474	0.6328	0.0038
LHA	CUL4,5	0.7046	0.4348	0.769	0.4346	0.0146
LHA	UVU	0.6809	0.3276	0.3828	0.6637	0.0111
LHA	CBN	0.5395	0.7134	0.4903	0.4635	0.4773
LHA	FL	0.5702	0.3424	0.4593	0.9993	0.7565
LHA	mESC B61	0.2802	0.0034	0.0004	0.0601	0.0007
LHA	mESC B62	0.2419	0.0021	0.0006	0.0401	0.0147
PVH	DMH	0.6866	0.8727	0.9096	0.4283	0.2858
PVH	VMH	0.7718	0.5616	0.9963	0.5884	0.0618
PVH	ARH	0.0005	0.8686	0.8037	0.0212	0.0182
PVH	PB	0.5385	0.1747	0.2236	0.185	0.0582
PVH	NTS	0.607	0.6181	0.2978	0.4329	0.1342
PVH	DVC	0.8162	0.5851	0.5341	0.834	0.2949
PVH	ENT	0.6748	0.5842	0.0331	0.2845	0.2702
PVH	CENT2	0.3665	0.077	0.2291	0.9487	0.0391
PVH	CUL4,5	0.4528	0.1952	0.4345	0.8109	0.1075
PVH	UVU	0.4338	0.1356	0.1742	0.9145	0.0878
PVH	CBN	0.3248	0.8819	0.2394	0.2023	0.1221

Supplemental Table 7 | Cont.

Region 1	Region 2	Monogenic	Rare	Low-frequency	GWAS	Syndromic
PVH	FL	0.3478	0.1434	0.22	0.5883	0.5982
PVH	mESC B61	0.1472	0.0006	<0.0001	0.0155	<0.0001
PVH	mESC B62	0.1237	0.0003	<0.0001	0.0095	0.0011
DMH	VMH	0.9097	0.6742	0.9058	0.8018	0.4223
DMH	ARH	0.0021	0.9959	0.8927	0.1304	0.1942
DMH	PB	0.3089	0.2311	0.2696	0.0342	0.0031
DMH	NTS	0.3591	0.7351	0.3534	0.115	0.0105
DMH	DVC	0.525	0.6997	0.6112	0.3165	0.0347
DMH	ENT	0.4109	0.6987	0.0434	0.0626	0.0303
DMH	CENT2	0.1921	0.1075	0.2759	0.3917	0.318
DMH	CUL4,5	0.249	0.2561	0.504	0.5803	0.5871
DMH	UVU	0.2361	0.1825	0.2128	0.3684	0.5215
DMH	CBN	0.1658	0.9906	0.2875	0.0388	0.0091
DMH	FL	0.1801	0.1923	0.2655	0.1824	0.1111
DMH	mESC B61	0.0645	0.0011	0.0001	0.0013	<0.0001
DMH	mESC B62	0.0526	0.0006	0.0002	0.0007	<0.0001
VMH	ARH	0.0014	0.678	0.8001	0.0779	0.6194
VMH	PB	0.3657	0.4363	0.2219	0.062	0.0002
VMH	NTS	0.4214	0.9346	0.2956	0.1851	0.0008
VMH	DVC	0.6014	0.9724	0.531	0.4528	0.0036
VMH	ENT	0.4782	0.9734	0.0327	0.1071	0.0031
VMH	CENT2	0.2333	0.2335	0.2273	0.5448	0.8443
VMH	CUL4,5	0.2984	0.4737	0.4317	0.7627	0.7953
VMH	UVU	0.2838	0.3606	0.1727	0.5166	0.8717
VMH	CBN	0.2029	0.6657	0.2375	0.0694	0.0007
VMH	FL	0.2195	0.3762	0.2183	0.2791	0.0168
VMH	mESC B61	0.0824	0.0042	<0.0001	0.0031	<0.0001
VMH	mESC B62	0.0678	0.0026	<0.0001	0.0017	<0.0001
ARH	PB	<0.0001	0.2331	0.3323	0.0003	<0.0001
ARH	NTS	<0.0001	0.739	0.4275	0.002	0.0001
ARH	DVC	0.0002	0.7035	0.7088	0.012	0.0007
ARH	ENT	0.0001	0.7026	0.0592	0.0007	0.0006
ARH	CENT2	<0.0001	0.1086	0.3394	0.0179	0.764
ARH	CUL4,5	<0.0001	0.2582	0.5937	0.0389	0.4496
ARH	UVU	<0.0001	0.1842	0.2662	0.0159	0.5105
ARH	CBN	<0.0001	0.9865	0.3528	0.0003	0.0001
ARH	FL	<0.0001	0.194	0.3275	0.0044	0.0039
ARH	mESC B61	<0.0001	0.0011	0.0002	<0.0001	<0.0001
ARH	mESC B62	<0.0001	0.0006	0.0003	<0.0001	<0.0001
PB	NTS	0.9198	0.3896	0.8598	0.5882	0.6909
PB	DVC	0.7019	0.4163	0.5506	0.2644	0.3959
PB	ENT	0.8448	0.417	0.3557	0.7985	0.4274
PB	CENT2	0.7729	0.6788	0.9886	0.2072	<0.0001
PB	CUL4,5	0.8919	0.9505	0.6621	0.1176	0.0005
PB	UVU	0.8666	0.8917	0.8864	0.2231	0.0003
PB	CBN	0.711	0.2265	0.9676	0.9597	0.7269

Supplemental Table 7 | Cont.

Region 1	Region 2	Monogenic	Rare	Low-frequency	GWAS	Syndromic
PB	FL	0.7454	0.915	0.9923	0.4328	0.171
PB	mESC B61	0.4024	0.0357	0.0047	0.2728	0.0199
PB	mESC B62	0.3535	0.0243	0.0064	0.2041	0.1663
NTS	DVC	0.7778	0.9622	0.6741	0.5655	0.6517
NTS	ENT	0.9243	0.9611	0.2716	0.7747	0.692
NTS	CENT2	0.6971	0.203	0.8711	0.4716	0.0004
NTS	CUL4,5	0.8129	0.4247	0.7945	0.3061	0.002
NTS	UVU	0.7881	0.3192	0.7494	0.4985	0.0014
NTS	CBN	0.6375	0.7263	0.8918	0.6235	0.9614
NTS	FL	0.6706	0.3337	0.8523	0.8081	0.3311
NTS	mESC B61	0.3484	0.0032	0.0027	0.1014	0.0065
NTS	mESC B62	0.3038	0.002	0.0038	0.0701	0.075
DVC	ENT	0.8515	0.999	0.1292	0.3893	0.9559
DVC	CENT2	0.5022	0.2202	0.5602	0.8846	0.0019
DVC	CUL4,5	0.604	0.4527	0.8728	0.6536	0.008
DVC	UVU	0.5819	0.3427	0.4596	0.9186	0.006
DVC	CBN	0.4515	0.691	0.578	0.2866	0.6172
DVC	FL	0.4795	0.3579	0.5442	0.7401	0.6025
DVC	mESC B61	0.223	0.0037	0.0007	0.027	0.0015
DVC	mESC B62	0.1906	0.0023	0.001	0.0171	0.0258
ENT	CENT2	0.6283	0.2207	0.3483	0.3144	0.0016
ENT	CUL4,5	0.7402	0.4535	0.1743	0.1904	0.0068
ENT	UVU	0.7161	0.3434	0.4347	0.3355	0.0051
ENT	CBN	0.5714	0.6901	0.335	0.8377	0.6566
ENT	FL	0.6029	0.3586	0.3607	0.5967	0.5646
ENT	mESC B61	0.3019	0.0038	0.0543	0.1764	0.0018
ENT	mESC B62	0.2615	0.0023	0.069	0.1272	0.0296
CENT2	CUL4,5	0.8787	0.6339	0.6725	0.7614	0.6485
CENT2	UVU	0.904	0.781	0.8751	0.9657	0.7204
CENT2	CBN	0.9347	0.105	0.9791	0.226	0.0003
CENT2	FL	0.9712	0.7585	0.9809	0.6334	0.0097
CENT2	mESC B61	0.5828	0.091	0.0045	0.0184	<0.0001
CENT2	mESC B62	0.5222	0.0652	0.0062	0.0114	<0.0001
CUL4,5	UVU	0.9744	0.8429	0.5621	0.7289	0.9219
CUL4,5	CBN	0.8145	0.2512	0.6918	0.13	0.0017
CUL4,5	FL	0.8503	0.866	0.6552	0.4351	0.0329
CUL4,5	mESC B61	0.4829	0.0306	0.0012	0.0078	<0.0001
CUL4,5	mESC B62	0.4282	0.0207	0.0017	0.0046	<0.0001
UVU	CBN	0.8395	0.1787	0.8545	0.2429	0.0012
UVU	FL	0.8755	0.9765	0.894	0.6643	0.0257
UVU	mESC B61	0.503	0.0493	0.0072	0.0207	<0.0001
UVU	mESC B62	0.447	0.0341	0.0097	0.0129	<0.0001
CBN	FL	0.9634	0.1883	0.96	0.463	0.3076
CBN	mESC B61	0.6402	0.001	0.0041	0.2513	0.0075
CBN	mESC B62	0.5768	0.0006	0.0057	0.1867	0.0832
FL	mESC B61	0.6078	0.0461	0.0048	0.06	0.0002

Supplemental Table 7 | Cont.

Region 1	Region 2	Monogenic	Rare	Low-frequency	GWAS	Syndromic
FL	mESC B62	0.5459	0.0317	0.0066	0.04	0.006
mESC B61	mESC B62	0.9276	0.8771	0.9145	0.8623	0.3433

Supplemental Table 8 | Frequency distribution of obesity-associated genes across all genetic categories. Table shows one-way ANOVA reporting the Benjamini, Krieger and Yekutieli two step false discovery rate corrected p-value. Relates to Supplemental Figure 5A.

Region 1	Region 2	Obesity
THy/PHy	F	0.1493
THy/PHy	M	0.0318
THy/PHy	H	0.762
THy/PHy	FRP	0.0595
THy/PHy	ACA	0.0348
THy/PHy	ACB	0.0353
THy/PHy	VTA	<0.0001
THy/PHy	LHA	0.0625
THy/PHy	PVH	0.3404
THy/PHy	DMH	0.928
THy/PHy	VMH	0.7456
THy/PHy	ARH	0.1536
THy/PHy	PB	0.0018
THy/PHy	NTS	0.0169
THy/PHy	DVC	0.0905
THy/PHy	ENT	0.0067
THy/PHy	CENT2	0.1453
THy/PHy	CUL4,5	0.2718
THy/PHy	UVU	0.1303
THy/PHy	CBN	0.0046
THy/PHy	FL	0.0275
THy/PHy	mESC B61	<0.0001
THy/PHy	mESC B62	<0.0001
F	M	0.0003
F	H	0.2547
F	FRP	0.6576
F	ACA	0.5035
F	ACB	0.5069
F	VTA	0.0042
F	LHA	0.6739
F	PVH	0.6251
F	DMH	0.1765
F	VMH	0.2638
F	ARH	0.0041
F	PB	0.0922
F	NTS	0.343
F	DVC	0.8016
F	ENT	0.2038
F	CENT2	0.9884
F	CUL4,5	0.7316
F	UVU	0.9433
F	CBN	0.1627
F	FL	0.4451

Supplemental Table 8 | Cont.

Region 1	Region 2	Obesity
F	mESC B61	0.0001
F	mESC B62	0.0001
M	H	0.0143
M	FRP	<0.0001
M	ACA	<0.0001
M	ACB	<0.0001
M	VTA	<0.0001
M	LHA	<0.0001
M	PVH	0.0019
M	DMH	0.0252
M	VMH	0.0135
M	ARH	0.4712
M	PB	<0.0001
M	NTS	<0.0001
M	DVC	0.0001
M	ENT	<0.0001
M	CENT2	0.0003
M	CUL4,5	0.0012
M	UVU	0.0003
M	CBN	<0.0001
M	FL	<0.0001
M	mESC B61	<0.0001
M	mESC B62	<0.0001
H	FRP	0.1136
H	ACA	0.0706
H	ACB	0.0715
H	VTA	<0.0001
H	LHA	0.1188
H	PVH	0.5154
H	DMH	0.8317
H	VMH	0.9827
H	ARH	0.0837
H	PB	0.0048
H	NTS	0.0369
H	DVC	0.1645
H	ENT	0.016
H	CENT2	0.2486
H	CUL4,5	0.4259
H	UVU	0.2262
H	CBN	0.0113
H	FL	0.0571
H	mESC B61	<0.0001
H	mESC B62	<0.0001
FRP	ACA	0.8213
FRP	ACB	0.8255
FRP	VTA	0.0156

Supplemental Table 8 | Cont.

Region 1	Region 2	Obesity
FRP	LHA	0.9821
FRP	PVH	0.3514
FRP	DMH	0.0727
FRP	VMH	0.1186
FRP	ARH	0.0009
FRP	PB	0.2147
FRP	NTS	0.6135
FRP	DVC	0.8478
FRP	ENT	0.4078
FRP	CENT2	0.6682
FRP	CUL4,5	0.4318
FRP	UVU	0.7098
FRP	CBN	0.3406
FRP	FL	0.7486
FRP	mESC B61	0.0007
FRP	mESC B62	0.0006
ACA	ACB	0.9957
ACA	VTA	0.0283
ACA	LHA	0.8039
ACA	PVH	0.247
ACA	DMH	0.0434
ACA	VMH	0.0741
ACA	ARH	0.0004
ACA	PB	0.3102
ACA	NTS	0.78
ACA	DVC	0.6761
ACA	ENT	0.5472
ACA	CENT2	0.5128
ACA	CUL4,5	0.3116
ACA	UVU	0.5499
ACA	CBN	0.4671
ACA	FL	0.9246
ACA	mESC B61	0.0016
ACA	mESC B62	0.0014
ACB	VTA	0.0279
ACB	LHA	0.8081
ACB	PVH	0.2492
ACB	DMH	0.0439
ACB	VMH	0.0749
ACB	ARH	0.0004
ACB	PB	0.3076
ACB	NTS	0.7759
ACB	DVC	0.68
ACB	ENT	0.5436
ACB	CENT2	0.5163
ACB	CUL4,5	0.3141

Supplemental Table 8 | Cont.

Region 1	Region 2	Obesity
ACB	UVU	0.5535
ACB	CBN	0.4638
ACB	FL	0.9204
ACB	mESC B61	0.0016
ACB	mESC B62	0.0013
VTA	LHA	0.0146
VTA	PVH	0.0008
VTA	DMH	<0.0001
VTA	VMH	<0.0001
VTA	ARH	<0.0001
VTA	PB	0.2384
VTA	NTS	0.0556
VTA	DVC	0.009
VTA	ENT	0.1114
VTA	CENT2	0.0044
VTA	CUL4,5	0.0014
VTA	UVU	0.0053
VTA	CBN	0.1425
VTA	FL	0.0358
VTA	mESC B61	0.3355
VTA	mESC B62	0.3131
LHA	PVH	0.3631
LHA	DMH	0.0764
LHA	VMH	0.124
LHA	ARH	0.001
LHA	PB	0.2066
LHA	NTS	0.5978
LHA	DVC	0.8654
LHA	ENT	0.3952
LHA	CENT2	0.6846
LHA	CUL4,5	0.445
LHA	UVU	0.7266
LHA	CBN	0.3294
LHA	FL	0.7317
LHA	mESC B61	0.0007
LHA	mESC B62	0.0006
PVH	DMH	0.3881
PVH	VMH	0.5294
PVH	ARH	0.0173
PVH	PB	0.0299
PVH	NTS	0.1508
PVH	DVC	0.4594
PVH	ENT	0.0785
PVH	CENT2	0.6148
PVH	CUL4,5	0.8842
PVH	UVU	0.5756

Supplemental Table 8 | Cont.

Region 1	Region 2	Obesity
PVH	CBN	0.0595
PVH	FL	0.2105
PVH	mESC B61	<0.0001
PVH	mESC B62	<0.0001
DMH	VMH	0.8149
DMH	ARH	0.1292
DMH	PB	0.0024
DMH	NTS	0.0215
DMH	DVC	0.109
DMH	ENT	0.0087
DMH	CENT2	0.1719
DMH	CUL4,5	0.3132
DMH	UVU	0.1548
DMH	CBN	0.006
DMH	FL	0.0344
DMH	mESC B61	<0.0001
DMH	mESC B62	<0.0001
VMH	ARH	0.0799
VMH	PB	0.0051
VMH	NTS	0.0389
VMH	DVC	0.1711
VMH	ENT	0.0169
VMH	CENT2	0.2576
VMH	CUL4,5	0.4386
VMH	UVU	0.2346
VMH	CBN	0.012
VMH	FL	0.06
VMH	mESC B61	<0.0001
VMH	mESC B62	<0.0001
ARH	PB	<0.0001
ARH	NTS	0.0001
ARH	DVC	0.0018
ARH	ENT	<0.0001
ARH	CENT2	0.0039
ARH	CUL4,5	0.0116
ARH	UVU	0.0033
ARH	CBN	<0.0001
ARH	FL	0.0003
ARH	mESC B61	<0.0001
ARH	mESC B62	<0.0001
PB	NTS	0.462
PB	DVC	0.152
PB	ENT	0.6797
PB	CENT2	0.0951
PB	CUL4,5	0.0427
PB	UVU	0.1068

Supplemental Table 8 | Cont.

Region 1	Region 2	Obesity
PB	CBN	0.7736
PB	FL	0.3574
PB	mESC B61	0.0322
PB	mESC B62	0.0287
NTS	DVC	0.4857
NTS	ENT	0.7469
NTS	CENT2	0.3504
NTS	CUL4,5	0.1966
NTS	UVU	0.3804
NTS	CBN	0.6542
NTS	FL	0.8534
NTS	mESC B61	0.004
NTS	mESC B62	0.0035
DVC	ENT	0.3078
DVC	CENT2	0.8129
DVC	CUL4,5	0.5524
DVC	UVU	0.8571
DVC	CBN	0.2522
DVC	FL	0.6084
DVC	mESC B61	0.0004
DVC	mESC B62	0.0003
ENT	CENT2	0.209
ENT	CUL4,5	0.1066
ENT	UVU	0.2302
ENT	CBN	0.9004
ENT	FL	0.6119
ENT	mESC B61	0.0106
ENT	mESC B62	0.0093
CENT2	CUL4,5	0.7207
CENT2	UVU	0.9549
CENT2	CBN	0.1671
CENT2	FL	0.4538
CENT2	mESC B61	0.0001
CENT2	mESC B62	0.0001
CUL4,5	UVU	0.6788
CUL4,5	CBN	0.082
CUL4,5	FL	0.2685
CUL4,5	mESC B61	<0.0001
CUL4,5	mESC B62	<0.0001
UVU	CBN	0.1852
UVU	FL	0.4886
UVU	mESC B61	0.0002
UVU	mESC B62	0.0001
CBN	FL	0.527
CBN	mESC B61	0.0151
CBN	mESC B62	0.0133

Supplemental Table 8 | Cont.

Region 1	Region 2	Obesity
FL	mESC B61	0.0022
FL	mESC B62	0.0019
mESC B61	mESC B62	0.9636

Supplementary Table 9 | WGCNA analysis of obesity-associated genes. Description of each gene including: color key and position in the WGCNA dendrogram, position in the hierarchical cluster dendrogram, module number, gene name following the heatmap order and genetic category. Relates to Fig. 5A. Heatmap in Fig 5A and table are in the same order.

Color key WGCNA dendrogram	WGCNA dendrogram	Hierarchical cluster dendrogram	Module #	Gene name in heatmap	Genetic category
	182	2	1	Tcf7l2	GWAS
	2	3	1	Sim1	Monogenic
	160	4	1	Smg6	GWAS
	28	10	1	Lepr	Monogenic/GWAS
	18	15	1	Bbs12	Syndromic
	64	16	1	Stk33	GWAS
	169	41	1	Pbrm1	GWAS
	175	44	1	Hmgcr	GWAS
	3	45	1	Plxna1	Rare variants
	188	46	1	Stk36	GWAS
	96	47	1	Plxna3	Rare variants
	73	48	1	Ptbp2	GWAS
	170	49	1	Zfp608	GWAS
	125	50	1	Fbxl19	GWAS
	104	51	1	Kctd15	GWAS
	14	53	1	Zfp668	GWAS
	117	54	1	Elavl4	GWAS
	105	56	1	Slc39a8	GWAS
	90	57	1	Ift74	Syndromic
	68	58	1	Nrp2	Rare variants
	158	59	1	Ehbp1	GWAS
	34	60	1	Nrp1	Rare variants
	78	61	1	Nav1	GWAS
	114	62	1	Fign	GWAS
	16	63	1	Zfhx3	Low variants
	177	64	1	Pgpep1	GWAS
	97	65	1	Tal1	GWAS
	184	66	1	Tfap2b	GWAS
	81	67	1	Gipr	GWAS/Rare
	32	68	1	Lmo1	GWAS
	120	72	1	Olig3	GWAS
	59	74	1	Lmx1b	GWAS
	91	75	1	Rpgrip1l	Syndromic
	66	78	1	Plxna2	Rare variants
	10	178	1	Iars2	GWAS
	111	179	1	Mkks	Syndromic
	50	182	1	Tmem160	GWAS

Supplementary Table 9 | Cont.

Color key WGCNA dendrogram	WGCNA dendrogram	Hierarchical cluster dendrogram	Module #	Gene name in heatmap	Genetic category
	147	34	2	Nudt3	GWAS
	57	70	2	Lztfl1	Syndromic
	153	80	2	Etv5	GWAS
	107	82	2	Polk	GWAS
	136	86	2	Nlrc3	GWAS
	106	87	2	Rab23	Syndromic
	24	88	2	Rcc1l	GWAS
	121	89	2	Tomm40	GWAS
	145	90	2	Adpgk	GWAS
	139	91	2	Gdf15	GWAS
	71	92	2	Hmga1	GWAS
	190	93	2	Zfp64	GWAS
	49	94	2	Fancl	GWAS
	29	95	2	Mtfr2	GWAS
	54	96	2	Mks1	Syndromic
	168	97	2	Gtf3a	GWAS
	79	98	2	Snrpd2	GWAS
	33	99	2	Ttll4	GWAS
	124	101	2	Kat8	GWAS
	130	102	2	Sfxn2	GWAS
	103	103	2	Usp37	GWAS
	86	104	2	Cnot9	GWAS
	142	105	2	Ino80e	GWAS
	146	106	2	Nup54	GWAS
	109	107	2	Rpl27a	GWAS
	152	109	2	Sema3d	Rare variants
	60	168	2	Sema3c	Rare variants
	46	169	2	Plxna4	Rare variants
	122	170	2	Rabep1	GWAS
	21	110	3	Rapgef3	Low variants
	180	111	3	Trim66	GWAS
	31	112	3	Ache	Low variants
	41	113	3	Rit2	GWAS
	70	114	3	Kcnk9	GWAS
	183	115	3	Faim2	GWAS
	164	116	3	Col4a3bp	GWAS
	154	117	3	Dnajc27	GWAS
	113	118	3	Hsd17b12	GWAS
	75	119	3	Gbe1	GWAS
	61	120	3	Scarb2	GWAS

Supplementary Table 9 | Cont.

Color key WGCNA dendrogram	WGCNA dendrogram	Hierarchical cluster dendrogram	Module #	Gene name in heatmap	Genetic category
	110	130	3	Efr3b	GWAS
	149	135	3	Ifngr1	GWAS
	176	136	3	Gnpda2	GWAS
	155	137	3	Rab21	Low variants
	108	138	3	Cep290	Syndromic
	23	140	3	Tmem18	GWAS
	47	147	3	Fto	GWAS
	100	148	3	Nt5c2	GWAS
	143	177	3	Ankdd1b	GWAS
	38	183	3	Cobll1	GWAS
	37	184	3	Cyp27a1	GWAS
	76	185	3	Pcdh9	GWAS
	55	186	3	Plcd4	GWAS
	30	187	3	Gprc5b	GWAS
	48	188	3	Ssfa2	GWAS
	89	189	3	Hhip	GWAS
	74	190	3	Tlr4	GWAS
	65	1	4	Gpr151	Rare variants
	27	14	4	Prkd1	GWAS
	17	79	4	Wdpcp	Syndromic
	140	108	4	Mc4r	Monogenic/GWAS
	19	128	4	Ralyl	GWAS
	15	151	4	Slc22a3	GWAS
	151	152	4	Cadm1	GWAS
	115	153	4	Grid1	GWAS
	156	154	4	Lingo2	GWAS
	36	155	4	Lrfn2	GWAS
	132	156	4	Adcy3	GWAS
	7	157	4	Bbs4	GWAS/Syndromic
	94	158	4	Ntrk2	Monogenic/GWAS
	127	159	4	Lrp1b	GWAS
	129	160	4	Park2	GWAS
	150	161	4	Entpd6	Low variants
	148	162	4	Pcsk1	Monogenic
	157	164	4	Bdnf	Monogenic/GWAS
	63	165	4	Grp	GWAS
	178	166	4	Agbl4	GWAS
	189	167	4	Doc2a	GWAS
	102	171	4	Erbb4	GWAS
	171	173	4	Mapk3	GWAS

Supplementary Table 9 | Cont.

Color key WGCNA dendrogram	WGCNA dendrogram	Hierarchical cluster dendrogram	Module #	Gene name in heatmap	Genetic category
	137	175	4	Cadm2	GWAS
	165	19	5	Inpp5e	Syndromic
	166	20	5	Cbln1	GWAS
	6	21	5	Kcnk3	GWAS
	80	22	5	Foxo3	GWAS
	99	23	5	Zfp142	GWAS
	8	35	5	Zfp169	Low variants
	187	37	5	Poc5	GWAS
	44	69	5	Mtif3	GWAS
	167	123	5	Ksr2	Low variants
	128	124	5	Nrxn3	GWAS
	72	125	5	Dmxl2	GWAS
	161	126	5	Frrs1l	GWAS
	26	131	5	Stx1b	GWAS
	133	132	5	Ypel3	GWAS
	95	133	5	Ric3	GWAS
	84	134	5	Scg3	GWAS
	101	139	5	Epb41l4b	GWAS
	11	180	5	Zbtb7b	Low variants
	5	18	6	Rasa2	GWAS
	92	76	6	Rarb	GWAS
	1	121	6	Negr1	GWAS
	53	122	6	Arl6	Syndromic
	159	127	6	Trim32	Syndromic
	126	129	6	Zfr2	Low variants
	83	141	6	Bbs5	Syndromic
	98	142	6	Ets2	GWAS
	9	143	6	Bbs1	Syndromic
	56	144	6	Bbs7	Syndromic
	174	145	6	Bbs2	Syndromic
	51	146	6	Ttc8	Syndromic
	62	149	6	Qpctl	GWAS
	144	38	7	Hif1an	GWAS
	69	39	7	Cep295	GWAS
	42	40	7	Zbtb10	GWAS
	118	83	7	Cdkal1	GWAS
	58	84	7	Alms1	Syndromic
	116	85	7	Mtch2	GWAS
	172	100	7	Zfp646	GWAS
	77	163	7	Tnni3k	GWAS

Supplementary Table 9 | Cont.

Color key WGCNA dendrogram	WGCNA dendrogram	Hierarchical cluster dendrogram	Module #	Gene name in heatmap	Genetic category
	52	172	7	Kctd13	GWAS
	186	174	7	Fhit	GWAS
	181	176	7	Stxbp6	GWAS
	173	24	8	Pex2	GWAS
	162	25	8	Ift27	Syndromic
	40	26	8	Txndc12	GWAS
	119	27	8	Fam57b	GWAS
	4	31	8	Sdccag8	Syndromic
	112	32	8	Sh2b1	Monogenic/GWAS
	131	33	8	Map2k5	GWAS
	22	36	8	Bbip1	Syndromic
	87	71	8	Sema3a	Rare variants
	185	150	8	Taok2	GWAS
	43	28	9	Irs1	GWAS
	45	29	9	Hip1	GWAS
	20	30	9	Sbk1	GWAS
	67	42	9	Creb1	GWAS
	134	43	9	Phip	Syndromic
	135	52	9	Ube2e3	GWAS
	25	55	9	Klf7	GWAS
	39	73	9	Skor1	GWAS
	93	5	10	Calcr	GWAS
	13	6	10	Bbs9	Syndromic
	82	7	10	Tub	Monogenic/GWAS
	12	8	10	Asb4	GWAS
	138	9	10	Pomc	Monogenic/GWAS
	141	11	10	Vkorc1	GWAS
	123	12	10	Bbs10	Syndromic
	85	13	10	Galnt10	GWAS
	163	17	11	Cyp17a1	GWAS
	35	77	11	Sec16b	GWAS
	179	81	11	Apobr	GWAS
	88	181	11	Sema3b	Rare variants

Supplemental Table 10 | Type 1 diabetes-associated genes.

Gene Name	Genetic Category
Bach2	GWAS
C1qtnf6	GWAS
Cars	GWAS
Cd69	GWAS
Cdk18	GWAS
Cenpw	GWAS
Clec16a	GWAS
Cobl	GWAS
Ctla4	GWAS
Ctsh	GWAS
Cux2	Genomes-based Imputation
Dlk1	GWAS
Efr3b	GWAS
Erap1	TaqMan Genotyping
Erbb3	GWAS
Fam189a2	GWAS
Fosl2	GWAS
Frmd3	GWAS
Glis3	GWAS
Herc2	GWAS
Htr1a	Genotyping and Sequencing
Ifih1	GWAS
Ifng	GWAS
Ikzf4	GWAS
Il10	GWAS
Il2	GWAS
Il27	GWAS
Il2ra	GWAS
Ins2	GWAS
Itpr3	TaqMan Genotyping
Kcnj11	GWAS
Kif5a	TaqMan Genotyping
Lmo7	GWAS
Lpar3	GWAS
Mapk14	GWAS
Ormdl3	GWAS
Pgm1	GWAS
Phtf1	GWAS
Pip4k2c	TaqMan Genotyping
Pkd1	GWAS
Plekha1	Dense Genotyping on the ImmunoChip
Pparg	GWAS
Ppil2	Dense Genotyping on the ImmunoChip
Prkcq	TaqMan Genotyping
Prkd2	GWAS
Ptpn2	GWAS

Supplemental Table 10 | Cont.

Gene Name	Genetic Category
Ptpn22	GWAS
Rasgrp1	GWAS
Rnaset2a	Dense Genotyping on the ImmunoChip
Rnls	TaqMan genotyping
Sh2b3	TaqMan genotyping
Skap2	TaqMan genotyping
Stat3	TaqMan genotyping
Stat4	TaqMan genotyping
Suox	GWAS
Tagap	GWAS
Tcf7l2	GWAS
Tnfaip3	TaqMan Genotyping
Tnfrsf11b	GWAS
Tyk2	GWAS
Ubash3a	GWAS

Supplemental Table 11 | Type 2 diabetes-associated genes.

Gene Name	Genetic Category
Abcb11	GWAS
Abcc8	MODY
Abo	Rare
Adamts9	GWAS
Adcy5	GWAS
Adra2a	GWAS
Agmo	GWAS
Ank1	GWAS
Ankrd55	GWAS
Ap3s2	GWAS
Appl1	MODY
Arap1	GWAS
Arl15	GWAS
Atg13	Low-frequency
Bcar1	GWAS
Bcdin3d	GWAS
Bcl11a	GWAS
Blk	MODY
C2cd4a	GWAS
C2cd4b	GWAS
Camk1d	GWAS
Capn10	GWAS
Ccnd2	GWAS, Low-frequency
Cdc123	GWAS
Cdkal1	GWAS
Cdkn2a	GWAS
Cdkn2b	GWAS
Cel	MODY
Cilp2	GWAS
Cobll1	GWAS
Cry2	GWAS
Dgkb	GWAS
Dpysl5	GWAS-Glycemic Traits
Dusp9	GWAS
Fads1	GWAS
Faf1	GWAS
Faim2	GWAS
Fitm2	GWAS
Foxa2	GWAS-Glycemic Traits
Fto	GWAS
G6pc2	GWAS
Gatad2a	GWAS
Gcc1	GWAS
Gck	GWAS, MODY
Gckr	GWAS
Gipr	GWAS, GWAS-Glycemic Traits

Supplemental Table 11 | Cont.

Gene Name	Genetic Category
Glis3	GWAS
Gpsm1	Rare
Grb10	GWAS-Glycemic Traits
Grb14	GWAS
Grk5	GWAS
Hectd4	GWAS-Glycemic Traits
Hhex	GWAS
Hmg20a	GWAS
Hmga2	GWAS
Hnf1a	GWAS, MODY, Rare
Hnf1b	GWAS, MODY
Hnf4a	GWAS, MODY
Ide	GWAS
Igf1	GWAS
Igf2	GWAS
Igf2bp2	GWAS
Ins2	GWAS, MODY
Irs1	GWAS, GWAS-Glycemic Traits
Itgb6	GWAS
Jazf1	GWAS
Kank1	Low-frequency
Kcnj11	GWAS, MODY
Kcnk16	GWAS
Kcnq1	GWAS
Klf11	MODY
Klf14	GWAS
Klhl42	GWAS
Lama1	GWAS
Larp6	GWAS-Glycemic Traits
Lgr5	GWAS
Lpp	GWAS
Lyplal1	GWAS-Glycemic Traits
Macf1	GWAS
Madd	GWAS, Low-frequency
Maea	GWAS
Mc4r	GWAS
Mphosph9	GWAS
Msmo1	GWAS-Glycemic Traits
Mtnr1b	GWAS
Neurod1	MODY
Notch2	GWAS
Nyap2	GWAS
Oasl1	GWAS
Pam	Rare, Low-frequency
Pax4	GWAS, MODY
Pcsk1	GWAS-Glycemic Traits

Supplemental Table 11 | Cont.

Gene Name	Genetic Category
Pdgfc	GWAS-Glycemic Traits
Pdx1	GWAS-Glycemic Traits, MODY, Rare
Pepd	GWAS
Pou5f1	GWAS
Pparg	GWAS
Ppip5k2	Low-frequency
Ppp1r3b	GWAS-Glycemic Traits
Prc1	GWAS
Prox1	GWAS
Psmd6	GWAS
Ptprd	GWAS
R3hdml	GWAS
Rasgrp1	GWAS
Rbms1	GWAS
Rnd3	GWAS
Rreb1	GWAS
Sdhaf4	GWAS
Sgcg	GWAS
Sgsm2	GWAS-Glycemic Traits, Low-frequency
Slc2a2	GWAS-Glycemic Traits
Slc30a8	GWAS, Rare
Snx7	GWAS-Glycemic Traits
Spry2	GWAS
Srr	GWAS
Srsf2	GWAS
Ssr1	GWAS
St6gal1	GWAS
Sugp1	GWAS
Taf11	GWAS-Glycemic Traits
Tbc1d30	Low-frequency
Tcerg1l	GWAS-Glycemic Traits
Tcf19	GWAS
Tcf7	GWAS
Tcf7l2	GWAS
Thada	GWAS
Tle1	GWAS
Tle4	GWAS
Tmem163	GWAS
Trp53inp1	GWAS
Tspan8	GWAS
Ube2e2	GWAS
Vps13c	GWAS, GWAS-Glycemic Traits
Vps26a	GWAS
Wfs1	GWAS
Zbed3	GWAS
Zfand3	GWAS

Supplemental Table 11 | Cont.

Gene Name	Genetic Category
Zfand6	GWAS
Zmiz1	GWAS

Supplemental Table 12 | Autism-associated genes.

Gene Name	Genetic Category
Adnp	Rare Single Gene Mutation, Syndromic, Functional
Adsl	Rare Single Gene Mutation, Syndromic
Aff2	Rare Single Gene Mutation, Syndromic
Ahdc1	Rare Single Gene Mutation, Syndromic
Aldh5a1	Rare Single Gene Mutation, Syndromic
Ank2	Rare Single Gene Mutation
Ank3	Rare Single Gene Mutation, Genetic Association
Ankrd11	Rare Single Gene Mutation, Syndromic
Ap2s1	Rare Single Gene Mutation
Arhgef9	Rare Single Gene Mutation, Syndromic
Arid1b	Rare Single Gene Mutation, Syndromic
Arx	Rare Single Gene Mutation, Syndromic
Ash1l	Rare Single Gene Mutation, Syndromic
Asxl3	Rare Single Gene Mutation, Syndromic
Atrx	Rare Single Gene Mutation, Syndromic
Auts2	Rare Single Gene Mutation, Syndromic, Genetic Association
Baz2b	Rare Single Gene Mutation
Bckdk	Rare Single Gene Mutation
Bcl11a	Rare Single Gene Mutation, Syndromic
Braf	Rare Single Gene Mutation, Syndromic
Brsk2	Rare Single Gene Mutation, Syndromic
Cacna1c	Rare Single Gene Mutation, Syndromic, Genetic Association, Functional
Cacna1e	Rare Single Gene Mutation
Cacna2d3	Rare Single Gene Mutation
Cask	Rare Single Gene Mutation, Syndromic
Cdkl5	Rare Single Gene Mutation, Syndromic
Celf4	Rare Single Gene Mutation, Functional
Champ1	Rare Single Gene Mutation, Syndromic
Chd2	Rare Single Gene Mutation, Syndromic
Chd3	Rare Single Gene Mutation, Syndromic
Chd7	Rare Single Gene Mutation, Syndromic
Chd8	Rare Single Gene Mutation, Syndromic, Functional
Cic	Rare Single Gene Mutation, Functional
Cnot3	Rare Single Gene Mutation, Syndromic
Coro1a	Rare Single Gene Mutation
Crebbp	Rare Single Gene Mutation, Syndromic, Genetic Association
Ctcf	Rare Single Gene Mutation, Syndromic, Functional
Ctnnb1	Rare Single Gene Mutation, Syndromic
Cul3	Rare Single Gene Mutation, Genetic Association
D5Ertd579e	Rare Single Gene Mutation
Ddx3x	Rare Single Gene Mutation, Syndromic
Deaf1	Rare Single Gene Mutation, Syndromic
Dhcr7	Rare Single Gene Mutation, Syndromic
Dip2a	Rare Single Gene Mutation, Functional
Dlg4	Rare Single Gene Mutation, Syndromic, Functional
Dmpk	Rare Single Gene Mutation, Syndromic

Supplemental Table 12 | Cont.

Gene Name	Genetic Category
Dnmt3a	Rare Single Gene Mutation, Syndromic, Genetic Association
Dpysl2	Rare Single Gene Mutation, Genetic Association, Functional
Dscam	Rare Single Gene Mutation, Genetic Association
Dync1h1	Rare Single Gene Mutation, Syndromic
Dyrk1a	Rare Single Gene Mutation, Syndromic, Functional
Ebf3	Rare Single Gene Mutation, Syndromic
Ehmt1	Rare Single Gene Mutation, Syndromic, Functional
Eif3g	Rare Single Gene Mutation
Elavl3	Rare Single Gene Mutation
Ep300	Rare Single Gene Mutation, Syndromic
Fmr1	Rare Single Gene Mutation, Syndromic, Genetic Association, Functional
Foxg1	Rare Single Gene Mutation, Syndromic, Genetic Association
Foxp1	Rare Single Gene Mutation, Syndromic, Genetic Association, Functional
Foxp2	Rare Single Gene Mutation, Genetic Association
Gabrb2	Rare Single Gene Mutation
Gabrb3	Rare Single Gene Mutation, Genetic Association
Gfap	Rare Single Gene Mutation
Gigyf1	Rare Single Gene Mutation
Gigyf2	Rare Single Gene Mutation
Gnai1	Rare Single Gene Mutation
Gria2	Rare Single Gene Mutation
Grin2b	Rare Single Gene Mutation, Syndromic, Genetic Association
Hdlbp	Rare Single Gene Mutation
Hectd4	Rare Single Gene Mutation
Hivep2	Syndromic, Functional
Hnrnph2	Rare Single Gene Mutation, Syndromic
Hnrnpu	Rare Single Gene Mutation, Syndromic, Functional
Hras	Rare Single Gene Mutation, Syndromic, Genetic Association
Iqsec2	Rare Single Gene Mutation, Syndromic, Functional
Irf2bpl	Rare Single Gene Mutation, Syndromic
Kansl1	Rare Single Gene Mutation, Syndromic
Katnal2	Rare Single Gene Mutation
Kcnb1	Rare Single Gene Mutation, Syndromic
Kcnq3	Rare Single Gene Mutation, Genetic Association
Kdm3b	Syndromic
Kdm5b	Rare Single Gene Mutation, Syndromic, Functional
Kdm6b	Rare Single Gene Mutation, Syndromic
Kmt2a	Rare Single Gene Mutation, Syndromic
Kmt2c	Rare Single Gene Mutation, Syndromic, Functional
Kmt2e	Rare Single Gene Mutation, Syndromic, Genetic Association
Kmt5b	Rare Single Gene Mutation, Syndromic
Ldb1	Rare Single Gene Mutation
Lrrc4c	Rare Single Gene Mutation
Lztr1	Rare Single Gene Mutation, Syndromic
Magel2	Rare Single Gene Mutation, Syndromic
Map1a	Rare Single Gene Mutation

Supplemental Table 12 | Cont.

Gene Name	Genetic Category
Mbd5	Rare Single Gene Mutation, Syndromic, Functional
Mboat7	Rare Single Gene Mutation, Syndromic
Mecp2	Rare Single Gene Mutation, Syndromic, Functional
Med13	Rare Single Gene Mutation, Syndromic
Med13l	Rare Single Gene Mutation, Syndromic
Meis2	Rare Single Gene Mutation, Syndromic
Mkx	Rare Single Gene Mutation
Myt1l	Rare Single Gene Mutation, Syndromic, Genetic Association
Naa15	Rare Single Gene Mutation, Syndromic
Nacc1	Rare Single Gene Mutation, Syndromic
Nbea	Rare Single Gene Mutation, Syndromic, Functional
Nckap1	Rare Single Gene Mutation
Ncoa1	Rare Single Gene Mutation
Nf1	Rare Single Gene Mutation, Syndromic, Genetic Association
Nipbl	Rare Single Gene Mutation, Syndromic
Nlgn2	Rare Single Gene Mutation, Functional
Nlgn3	Rare Single Gene Mutation
Nr3c2	Rare Single Gene Mutation, Syndromic
Nr4a2	Rare Single Gene Mutation
Nrxn1	Rare Single Gene Mutation, Syndromic, Genetic Association, Functional
Nrxn2	Rare Single Gene Mutation, Genetic Association
Nrxn3	Rare Single Gene Mutation, Genetic Association
Nsd1	Rare Single Gene Mutation, Syndromic
Nup155	Rare Single Gene Mutation
Pacs1	Rare Single Gene Mutation, Syndromic
Pax5	Rare Single Gene Mutation
Pcdh19	Rare Single Gene Mutation, Syndromic, Functional
Phf12	Rare Single Gene Mutation
Phf2	Rare Single Gene Mutation
Phf21a	Rare Single Gene Mutation, Syndromic
Phf3	Rare Single Gene Mutation
Phip	Rare Single Gene Mutation, Syndromic
Pogz	Rare Single Gene Mutation, Syndromic, Functional
Pomgnt1	Rare Single Gene Mutation, Syndromic
Ppp1r9b	Rare Single Gene Mutation
Ppp2r5d	Rare Single Gene Mutation, Syndromic
Ppp5c	Rare Single Gene Mutation
Prr12	Rare Single Gene Mutation, Syndromic
Psmd12	Syndromic
Ptchd1	Rare Single Gene Mutation, Genetic Association
Pten	Rare Single Gene Mutation, Syndromic, Functional
Ptk7	Rare Single Gene Mutation
Ptpn11	Rare Single Gene Mutation, Syndromic
Rai1	Rare Single Gene Mutation, Syndromic
Reln	Rare Single Gene Mutation, Syndromic, Genetic Association, Functional
Rere	Rare Single Gene Mutation, Syndromic, Genetic Association

Supplemental Table 12 | Cont.

Gene Name	Genetic Category
Rfx3	Rare Single Gene Mutation
Rims1	Rare Single Gene Mutation, Genetic Association
Rorb	Rare Single Gene Mutation, Syndromic, Functional
Satb1	Rare Single Gene Mutation
Scn1a	Rare Single Gene Mutation, Syndromic, Genetic Association
Scn2a	Rare Single Gene Mutation, Syndromic
Scn8a	Rare Single Gene Mutation, Syndromic
Setbp1	Rare Single Gene Mutation, Syndromic
Setd2	Rare Single Gene Mutation, Syndromic
Setd5	Rare Single Gene Mutation, Syndromic
Shank2	Rare Single Gene Mutation, Syndromic, Genetic Association, Functional
Shank3	Rare Single Gene Mutation, Syndromic, Genetic Association, Functional
Sin3a	Rare Single Gene Mutation, Syndromic
Ski	Rare Single Gene Mutation
Slc6a1	Rare Single Gene Mutation, Syndromic, Genetic Association
Slc9a6	Rare Single Gene Mutation, Syndromic, Functional
Smarcc2	Rare Single Gene Mutation, Syndromic, Functional
Sox5	Rare Single Gene Mutation, Syndromic, Genetic Association
Spast	Rare Single Gene Mutation
Srcap	Rare Single Gene Mutation
Srpr	Rare Single Gene Mutation
Stxbp1	Rare Single Gene Mutation, Syndromic, Functional
Syngap1	Rare Single Gene Mutation, Syndromic
Tanc2	Rare Single Gene Mutation, Syndromic
Taok1	Rare Single Gene Mutation, Syndromic
Tbck	Syndromic
Tbl1xr1	Rare Single Gene Mutation, Syndromic
Tbr1	Rare Single Gene Mutation, Genetic Association, Functional
Tcf20	Rare Single Gene Mutation, Syndromic
Tcf4	Rare Single Gene Mutation, Syndromic, Genetic Association
Tcf7l2	Rare Single Gene Mutation
Tek	Rare Single Gene Mutation
Tlk2	Rare Single Gene Mutation, Syndromic
Tm9sf4	Rare Single Gene Mutation
Traf7	Rare Single Gene Mutation, Syndromic
Trim23	Rare Single Gene Mutation
Trio	Rare Single Gene Mutation, Syndromic
Trip12	Rare Single Gene Mutation, Syndromic
Tsc1	Rare Single Gene Mutation, Syndromic
Tsc2	Rare Single Gene Mutation, Syndromic, Genetic Association
Tshz3	Rare Single Gene Mutation, Functional
Ube3a	Rare Single Gene Mutation, Syndromic, Genetic Association
Ubr1	Rare Single Gene Mutation
Upf3b	Rare Single Gene Mutation, Syndromic
Vezf1	Rare Single Gene Mutation
Vps13b	Rare Single Gene Mutation, Syndromic

Supplemental Table 12 | Cont.

Gene Name	Genetic Category
Wac	Rare Single Gene Mutation, Syndromic
Wdfy3	Rare Single Gene Mutation, Syndromic, Functional
Zbtb20	Rare Single Gene Mutation, Syndromic
Zfp292	Rare Single Gene Mutation, Syndromic
Zfp462	Rare Single Gene Mutation, Syndromic
Zmynd8	Rare Single Gene Mutation

Supplemental Table 13 | Breast cancer-associated genes.

Gene Name	Genetic Category
Abraxas1	GWAS
Adcy3	GWAS
Ahrr	GWAS
Akap9	GWAS
Amfr	GWAS
Ap4b1	GWAS
Arrdc3	GWAS
Ascc2	GWAS
Atf7ip	GWAS
Atg10	GWAS
Atm	Hereditary
Atp6ap1l	GWAS
Bard1	Hereditary
Bcl2l15	GWAS
Brca1	Hereditary
Brca2	Hereditary
Brip1	Hereditary
Casp8	GWAS
Cbx6	GWAS
Ccdc127	GWAS
Ccdc170	GWAS
Ccdc91	GWAS
Ccne1	GWAS
Cdca7l	GWAS
Cdh1	Hereditary
Cend1	GWAS
Cenpo	GWAS
Cfl1	GWAS
Chek2	Hereditary
Coq5	GWAS
Cox11	GWAS
Cul5	GWAS
Cyp51	GWAS
Dclre1b	GWAS
Dda1	GWAS
Dffa	GWAS
Dnajc27	GWAS
Efemp2	GWAS
Ell	GWAS
Enpp7	GWAS
Epcam	Hereditary
Esr1	GWAS
Gatad2a	GWAS
Hapln4	GWAS
Hlf	GWAS
Hspa4	GWAS

Supplemental Table 13 | Cont.

Gene Name	Genetic Category
Kcnn4	GWAS
L3mbtl3	GWAS
Lrrc25	GWAS
Lrrd1	GWAS
Mchr1	GWAS
Mdm4	GWAS
Mlh1	Hereditary
Mrps18c	GWAS
Msh2	Hereditary
Msh6	Hereditary
Nbn	Hereditary
Nf1	Hereditary
Nol12	GWAS
Npat	GWAS
Nudt17	GWAS
Ocel1	GWAS
Ogfod1	GWAS
Palb2	Hereditary
Pex14	GWAS
Pgpep1	GWAS
Pidd1	GWAS
Pik3c2b	GWAS
Pms2	Hereditary
Ppil3	GWAS
Prc1	GWAS
Pten	Hereditary
Ptpn22	GWAS
Rad51c	Hereditary
Rad51d	Hereditary
Ralb	GWAS
Rccd1	GWAS
Rhbdd3	GWAS
Rnf115	GWAS
Rps23	GWAS
Sdha	GWAS
Sirt5	GWAS
Siva1	GWAS
Smg9	GWAS
Ssbp4	GWAS
Stk11	Hereditary
Syne1	GWAS
Tefm	GWAS
Tgfbr2	GWAS
Tlr1	GWAS
Tm6sf2	GWAS
Tmem184b	GWAS

Supplemental Table 13 | Cont.

Gene Name	Genetic Category
Tnnt3	GWAS
Tns1	GWAS
Trp53	Hereditary
Wdr43	GWAS
Zbtb38	GWAS
Zfp703	GWAS

Supplemental Table 14 | Expression levels of besity-, T1D-, T2D-, autism-, breast cancer-associated genes across the brain regions of energy balance. TPM values for genes associated to each were reported for all brain regions and mouse embryonic stem cell samples. For each region TPM value from each replicate were aggregated to calculate the mean and SD.

Supplemental Table 15 | Genes associated with more than one disease.

Gene Name	Obesity	T1D	T2D	Autism	Breast Cancer
Adcy3	x				x
Cdkal1	x		x		
Cobll1	x		x		
Dnajc27	x				x
Efr3b	x	x			
Faim2	x		x		
Fto	x		x		
Gipr	x		x		
Irs1	x		x		
Mc4r	x		x		
Nrxn3	x			x	
Pcsk1	x		x		
Pgpep1	x				x
Phip	x			x	
Tcf7l2	x	x	x	x	

Supplementary Table 16 | WGCNA analysis of obesity-, T1D-, T2D-, autism- and breast cancer-associated genes. Description of each gene including: color key and position in the WGCNA dendrogram, position in the hierarchical cluster dendrogram, module number, gene name following the heatmap order and genetic category. Relates to Fig. 7A.

Color key WGCNA dendrogram	WGCNA dendrogram	Hierarchical cluster dendrogram	Module #	Gene name in heatmap	Genetic category
	451	5	1	Ifngr1	Obesity
	537	6	1	Ctsh	T1D
	631	8	1	Ptpn11	Autism
	435	57	1	Efr3b	Obesity/T1D
	391	85	1	Tlr4	Obesity
	315	111	1	Ube3a	Autism
	155	114	1	Mtif3	Obesity
	335	189	1	Lztfl1	Obesity
	9	195	1	Erbb4	Obesity
	46	196	1	Mapk3	Obesity
	267	197	1	Sema3c	Obesity
	178	216	1	Slc9a6	Autism
	322	218	1	Zfr2	Obesity
	591	228	1	Vps26a	T2D
	131	229	1	Cobl	T1D
	549	230	1	Etv5	Obesity
	414	276	1	Rabep1	Obesity
	174	304	1	Apobr	Obesity
	402	306	1	Pepd	T2D
	453	308	1	Rreb1	T2D
	395	310	1	Spry2	T2D
	553	325	1	Itpr3	T1D
	552	326	1	Tyk2	T1D
	95	337	1	Tbl1xr1	Autism
	107	342	1	Pdgfc	T2D
	337	343	1	Nacc1	Autism
	158	344	1	Tomm40	Obesity
	132	345	1	Mrps18c	Breast cancer
	449	346	1	Rcc1l	Obesity
	63	347	1	Ino80e	Obesity
	578	348	1	Eif3g	Autism
	308	349	1	Rpl27a	Obesity
	355	350	1	Atm	Breast cancer
	490	351	1	Pidd1	Breast cancer
	373	352	1	Siva1	Breast cancer
	645	353	1	Fancl	Obesity
	244	354	1	Stat4	T1D
	255	355	1	Tefm	Breast cancer

Supplementary Table 16 | Cont.

Color key WGCNA dendrogram	WGCNA dendrogram	Hierarchical cluster dendrogram	Module #	Gene name in heatmap	Genetic category
	514	356	1	Mdm4	Breast cancer
	27	357	1	Nup54	Obesity
	240	358	1	Ddx3x	Autism
	588	359	1	Naa15	Autism
	253	360	1	Pgm1	T1D
	521	361	1	Il10	T1D
	11	362	1	Npat	Breast cancer
	181	363	1	Rad51c	Breast cancer
	620	364	1	Brca2	Breast cancer
	290	365	1	Adsl	Autism
	62	366	1	Cars	T1D
	384	367	1	Thada	T2D
	281	368	1	Cenpw	T1D
	511	369	1	Trp53	Breast cancer
	593	370	1	Palb2	Breast cancer
	121	371	1	Mtfr2	Obesity
	311	372	1	Brca1	Breast cancer
	438	373	1	Brip1	Breast cancer
	582	374	1	Gatad2a	T2D/Breast cancer
	5	375	1	Nup155	Autism
	319	376	1	Trip12	Autism
	227	377	1	Zfp64	Obesity
	232	378	1	Rps23	Breast cancer
	567	379	1	Tcf7	T2D
	81	380	1	Trp53inp1	T2D
	268	381	1	Abraxas1	Breast cancer
	496	382	1	Msh2	Breast cancer
	84	383	1	Wdr43	Breast cancer
	498	384	1	Hdlbp	Autism
	526	385	1	Epcam	Breast cancer
	590	386	1	Hnf4a	T2D
	531	387	1	Hspa4	Breast cancer
	303	388	1	Hmga1	Obesity
	461	389	1	Bard1	Breast cancer
	472	390	1	Chek2	Breast cancer
	277	391	1	Hhex	T2D
	82	392	1	Ubash3a	T1D
	371	393	1	Ccne1	Breast cancer
	318	394	1	Ide	T2D
	564	395	1	Cdh1	Breast cancer

Supplementary Table 16 | Cont.

Color key WGCNA dendrogram	WGCNA dendrogram	Hierarchical cluster dendrogram	Module #	Gene name in heatmap	Genetic category
	363	396	1	Msh6	Breast cancer
	554	397	1	Hnf1a	T2D
	478	398	1	Gdf15	Obesity
	572	399	1	Cdkn2a	T2D
	530	400	1	Dusp9	T2D
	411	401	1	Pou5f1	T2D
	642	402	1	G6pc2	T2D
	133	403	1	Polk	Obesity
	389	404	1	Adpgk	Obesity
	517	405	1	Psmd12	Autism
	347	406	1	R3hdml	T2D
	1	407	1	Abo	T2D
	463	408	1	Skap2	T1D
	31	409	1	Ap3s2	T2D
	245	410	1	Psmd6	T2D
	455	411	1	Ppil3	Breast cancer
	304	412	1	Dhcr7	Autism
	360	415	1	Gtf3a	Obesity
	546	416	1	Snrpd2	Obesity
	275	418	1	Cenpo	Breast cancer
	477	420	1	Cdca7l	Breast cancer
	509	421	1	Prc1	T2D/Breast cancer
	302	422	1	Cnot9	Obesity
	78	425	1	Lyplal1	T2D
	544	426	1	Faf1	T2D
	26	427	1	Klf11	T2D
	622	428	1	Lama1	T2D
	91	429	1	Kdm5b	Autism
	122	430	1	Ptk7	Autism
	151	544	1	Pex2	Obesity
	262	606	1	Dync1h1	Autism
	471	619	1	Zfp703	Breast cancer
	598	628	1	Nudt3	Obesity
	201	629	1	Brsk2	Autism
	37	630	1	Map2k5	Obesity
	536	638	1	Phtf1	T1D
	314	639	1	Dpysl2	Autism
	450	640	1	Fto	Obesity/T2D
	312	641	1	Nf1	Autism/Breast cancer

Supplementary Table 16 | Cont.

Color key WGCNA dendrogram	WGCNA dendrogram	Hierarchical cluster dendrogram	Module #	Gene name in heatmap	Genetic category
	508	645	1	Ptprd	T2D
	213	646	1	Ank3	Autism
	397	647	1	Plxna4	Obesity
	100	649	1	Tgfbr2	Breast cancer
	586	42	2	Iars2	Obesity
	378	112	2	Macf1	T2D
	462	113	2	Rere	Autism
	13	184	2	Bbs9	Obesity
	527	185	2	Klf14	T2D
	626	193	2	Tub	Obesity
	418	305	2	Cdkn2b	T2D
	377	314	2	Erbb3	T1D
	352	339	2	Nol12	Breast cancer
	24	434	2	Sim1	Obesity
	506	435	2	Dlk1	T1D
	90	436	2	Magel2	Autism
	79	437	2	Lrrd1	Breast cancer
	273	438	2	Akap9	Breast cancer
	116	439	2	Ikzf4	T1D
	35	440	2	Deaf1	Autism
	331	441	2	Prr12	Autism
	45	442	2	Plxna1	Obesity
	4	443	2	Smg6	Obesity
	189	444	2	Hip1	Obesity
	229	446	2	Zmiz1	T2D
	254	447	2	Stk36	Obesity
	265	448	2	Ptbp2	Obesity
	338	449	2	Vezf1	Autism
	568	450	2	Plxna3	Obesity
	556	451	2	Zfp608	Obesity
	152	452	2	Zfp668	Obesity
	561	453	2	Ldb1	Autism
	589	454	2	Zbed3	T2D
	69	455	2	Adnp	Autism
	18	456	2	Fbxl19	Obesity
	285	457	2	Kctd15	Obesity
	190	458	2	Spast	Autism
	246	459	2	Dpysl5	T2D
	324	460	2	Rnd3	T2D
	270	461	2	Bach2	T1D

Supplementary Table 16 | Cont.

Color key WGCNA dendrogram	WGCNA dendrogram	Hierarchical cluster dendrogram	Module #	Gene name in heatmap	Genetic category
	320	462	2	Tle1	T2D
	522	463	2	Casp8	Breast cancer
	257	464	2	St6gal1	T2D
	492	487	2	Champ1	Autism
	415	502	2	Ctnnb1	Autism
	194	503	2	Pbrm1	Obesity
	294	504	2	Ppil2	T1D
	566	505	2	Zfp462	Autism
	263	509	2	C1qtnf6	T1D
	431	515	2	Arx	Autism
	474	516	2	Ccnd2	T2D
	437	517	2	Cel	T2D
	585	534	2	Bbs12	Obesity
	148	542	2	Prox1	T2D
	205	543	2	Rnls	T1D
	510	545	2	Ift27	Obesity
	139	546	2	Txndc12	Obesity
	60	547	2	Lrrc25	Breast cancer
	447	548	2	Dda1	Breast cancer
	173	549	2	Hmg20a	T2D
	576	550	2	Rai1	Autism
	516	551	2	Capn10	T2D
	479	552	2	L3mbtl3	Breast cancer
	280	553	2	Nrp1	Obesity
	502	554	2	Glis3	T1D/T2D
	55	555	2	Grb10	T2D
	272	556	2	Ift74	Obesity
	258	557	2	Nrp2	Obesity
	408	558	2	Phf2	Autism
	452	559	2	Zfand3	T2D
	339	560	2	Setbp1	Autism
	200	561	2	Ube2e3	Obesity
	614	562	2	Dmpk	Autism
	125	563	2	Notch2	T2D
	195	564	2	Prkd2	T1D
	59	566	2	Tlk2	Autism
	231	568	2	Tcf7l2	Obesity/T1D/T2D/ Autism
	221	569	2	Pgpep1	Obesity/Breast cancer

Supplementary Table 16 | Cont.

Color key WGCNA dendrogram	WGCNA dendrogram	Hierarchical cluster dendrogram	Module #	Gene name in heatmap	Genetic category
	427	570	2	Ppip5k2	T2D
	630	571	2	Fign	Obesity
	291	572	2	Zfhx3	Obesity
	83	573	2	Asxl3	Autism
	110	574	2	Nav1	Obesity
	606	575	2	Aff2	Autism
	12	576	2	Slc39a8	Obesity
	241	577	2	Lmx1b	Obesity
	113	578	2	Ebf3	Autism
	374	580	2	Pik3c2b	Breast cancer
	605	581	2	Gipr	Obesity/T2D
	68	582	2	Lmo1	Obesity
	396	584	2	Tfap2b	Obesity
	141	585	2	Pax5	Autism
	612	586	2	Tal1	Obesity
	369	593	2	Tshz3	Autism
	298	599	2	Rpgrip1l	Obesity
	64	600	2	Cfl1	Breast cancer
	348	601	2	Hmgcr	Obesity
	209	602	2	Sox5	Autism
	332	603	2	Rfx3	Autism
	446	604	2	Bcl11a	T2D/Autism
	500	605	2	Plxna2	Obesity
	410	607	2	Elavl4	Obesity
	627	608	2	Tbck	Autism
	609	609	2	Cask	Autism
	641	610	2	Ehbp1	Obesity
	123	618	2	Sema3a	Obesity
	607	620	2	Gpsm1	T2D
	494	621	2	Klf7	Obesity
	616	622	2	Kmt2e	Autism
	219	623	2	Elavl3	Autism
	249	624	2	Smarcc2	Autism
	135	625	2	Efemp2	Breast cancer
	456	626	2	Irs1	Obesity/T2D
	545	632	2	Arid1b	Autism
	43	648	2	Frmd3	T1D
	507	1	3	Cep290	Obesity
	161	46	3	Tmem184b	Breast cancer
	77	47	3	Zbtb38	Breast cancer

Supplementary Table 16 | Cont.

Color key WGCNA dendrogram	WGCNA dendrogram	Hierarchical cluster dendrogram	Module #	Gene name in heatmap	Genetic category
	32	48	3	Mkks	Obesity
	67	49	3	Iqsec2	Autism
	611	50	3	Scn8a	Autism
	489	51	3	Slc6a1	Autism
	341	52	3	Coq5	Breast cancer
	167	53	3	Ric3	Obesity
	385	54	3	Scg3	Obesity
	105	55	3	Amfr	Breast cancer
	252	56	3	Wdfy3	Autism
	93	58	3	Pex14	Breast cancer
	501	59	3	Vps13c	T2D
	214	60	3	Stx1b	Obesity
	118	61	3	Stxbp1	Autism
	23	62	3	Kcnj11	T1D/T2D
	128	63	3	Ypel3	Obesity
	413	64	3	Clec16a	T1D
	224	65	3	Dmxl2	Obesity
	597	66	3	Madd	T2D
	525	67	3	Ank2	Autism
	250	68	3	Gnai1	Autism
	202	69	3	Gabrb2	Autism
	464	70	3	Nrxn3	Obesity/Autism
	448	72	3	Rnf115	Breast cancer
	573	81	3	Sirt5	Breast cancer
	644	121	3	Pomgnt1	Autism
	218	124	3	Ankrd11	Autism
	114	128	3	Cry2	T2D
	426	129	3	Scn2a	Autism
	197	130	3	Cbx6	Breast cancer
	185	131	3	Nrxn1	Autism
	592	132	3	Cacna1c	Autism
	547	133	3	Hlf	Breast cancer
	405	134	3	Arl6	Obesity
	259	135	3	Ash1l	Autism
	127	136	3	Ksr2	Obesity
	198	137	3	Cacna1e	Autism
	416	138	3	Negr1	Obesity
	358	139	3	Shank3	Autism
	104	140	3	Braf	Autism
	150	141	3	Ncoa1	Autism

Supplementary Table 16 | Cont.

Color key WGCNA dendrogram	WGCNA dendrogram	Hierarchical cluster dendrogram	Module #	Gene name in heatmap	Genetic category
	569	142	3	Atp6ap1l	Breast cancer
	562	143	3	Grk5	T2D
	282	145	3	Ppp5c	Autism
	193	146	3	Frrs1l	Obesity
	188	148	3	Nrxn2	Autism
	204	149	3	Rims1	Autism
	488	150	3	Shank2	Autism
	366	153	3	Rasa2	Obesity
	208	154	3	Appl1	T2D
	433	155	3	Taok1	Autism
	575	160	3	Ccdc127	Breast cancer
	376	162	3	Epb41l4b	Obesity
	628	163	3	Ubr1	Autism
	66	164	3	Bbs5	Obesity
	400	165	3	Ets2	Obesity
	541	166	3	Tmem18	Obesity
	469	167	3	Vps13b	Autism
	548	168	3	Atg13	T2D
	523	169	3	Trim23	Autism
	261	235	3	Bdnf	Obesity
	466	236	3	Pip4k2c	T1D
	176	237	3	Pparg	T1D/T2D
	361	260	3	Hivep2	Autism
	187	269	3	Ocel1	Breast cancer
	86	278	3	Tbc1d30	T2D
	484	279	3	Hectd4	T2D/Autism
	560	280	3	Trim32	Obesity
	621	282	3	Mbd5	Autism
	467	340	3	Tle4	T2D
	306	413	3	Mks1	Obesity
	212	414	3	Ascc2	Breast cancer
	140	431	3	Adamts9	T2D
	382	525	3	Gckr	T2D
	618	527	3	Ppp2r5d	Autism
	276	565	3	Cyp51	Breast cancer
	143	616	3	Hnrnph2	Autism
	558	617	3	Tcf4	Autism
	54	2	4	Kank1	T2D
	635	3	4	Erap1	T1D
	75	4	4	Fam189a2	T1D

Supplementary Table 16 | Cont.

Color key WGCNA dendrogram	WGCNA dendrogram	Hierarchical cluster dendrogram	Module #	Gene name in heatmap	Genetic category
	540	7	4	Rab21	Obesity
	503	9	4	Aldh5a1	Autism
	570	10	4	Gnpda2	Obesity
	247	12	4	Ssfa2	Obesity
	637	13	4	Fitm2	T2D
	632	14	4	Gprc5b	Obesity
	368	15	4	Hapln4	Breast cancer
	186	16	4	Scn1a	Autism
	216	17	4	Cend1	Breast cancer
	412	18	4	Dnajc27	Obesity/Breast cancer
	648	20	4	Dip2a	Autism
	499	21	4	Pcdh9	Obesity
	432	23	4	Larp6	T2D
	454	24	4	Rapgef3	Obesity
	15	26	4	Prkcq	T1D
	579	27	4	Kcnk9	Obesity
	357	28	4	Sdha	Breast cancer
	53	29	4	Col4a3bp	Obesity
	359	30	4	Faim2	Obesity/T2D
	22	31	4	Ifih1	T1D
	14	32	4	Tns1	Breast cancer
	638	33	4	Hsd17b12	Obesity
	444	34	4	Gbe1	Obesity
	108	35	4	Scarb2	Obesity
	559	36	4	Ccdc91	Breast cancer
	353	37	4	Plcd4	Obesity
	321	38	4	Ankrd55	T2D
	398	39	4	Ache	Obesity
	159	40	4	Rit2	Obesity
	44	41	4	Cox11	Breast cancer
	404	43	4	Rad51d	Breast cancer
	596	44	4	Lgr5	T2D
	458	86	4	Gfap	Autism
	639	87	4	Hhip	Obesity
	475	88	4	Il2ra	T1D
	297	89	4	Tek	Autism
	617	90	4	Cobll1	Obesity/T2D
	154	91	4	Cyp27a1	Obesity
	482	92	4	Ankdd1b	Obesity

Supplementary Table 16 | Cont.

Color key WGCNA dendrogram	WGCNA dendrogram	Hierarchical cluster dendrogram	Module #	Gene name in heatmap	Genetic category
	96	200	4	Adra2a	T2D
	401	203	4	Trim66	Obesity
	423	205	4	Fads1	T2D
	230	219	4	Mc4r	Obesity/T2D
	206	220	4	Msmo1	T2D
	519	221	4	Slc30a8	T2D
	390	222	4	Maea	T2D
	56	335	4	Arl15	T2D
	70	336	4	Med13l	Autism
	76	338	4	Tcf20	Autism
	424	536	4	Cdc123	T2D
	260	541	4	Rnaset2a	T1D
	146	595	4	Sema3b	Obesity
	529	11	5	Dffa	Breast cancer
	242	19	5	Map1a	Autism
	289	22	5	Agmo	T2D
	61	25	5	Grb14	T2D
	383	84	5	Tmem160	Obesity
	587	199	5	Kif5a	T1D
	283	204	5	Atg10	Breast cancer
	278	206	5	Sdhaf4	T2D
	542	214	5	Park2	Obesity
	574	287	5	Nr3c2	Autism
	284	315	5	Zmynd8	Autism
	215	316	5	Atrx	Autism
	248	317	5	Atf7ip	Breast cancer
	325	318	5	Phf3	Autism
	425	319	5	Fmr1	Autism
	646	320	5	Gigyf2	Autism
	296	341	5	Cilp2	T2D
	417	423	5	Srsf2	T2D
	565	424	5	Ralb	Breast cancer
	169	432	5	Cul5	Breast cancer
	192	467	5	Phf21a	Autism
	326	468	5	Wac	Autism
	30	469	5	Baz2b	Autism
	625	470	5	Kmt5b	Autism
	392	471	5	Chd2	Autism
	80	472	5	Upf3b	Autism
	649	473	5	Crebbp	Autism

Supplementary Table 16 | Cont.

Color key WGCNA dendrogram	WGCNA dendrogram	Hierarchical cluster dendrogram	Module #	Gene name in heatmap	Genetic category
	177	474	5	Kmt2a	Autism
	238	475	5	Auts2	Autism
	445	476	5	Dyrk1a	Autism
	428	479	5	Hnrnpu	Autism
	441	483	5	Med13	Autism
	367	488	5	Creb1	Obesity
	504	489	5	Phip	Obesity/Autism
	74	490	5	Rccd1	Breast cancer
	636	491	5	Cep295	Obesity
	112	492	5	Dnmt3a	Autism
	333	493	5	Nipbl	Autism
	34	494	5	Zbtb10	Obesity
	295	495	5	Setd5	Autism
	220	496	5	Mlh1	Breast cancer
	228	497	5	Traf7	Autism
	20	498	5	Hmga2	T2D
	2	499	5	Igf2bp2	T2D
	624	500	5	Cnot3	Autism
	72	501	5	Sin3a	Autism
	144	506	5	Pogz	Autism
	643	507	5	Srcap	Autism
	407	508	5	Kdm6b	Autism
	88	510	5	Ehmt1	Autism
	102	511	5	Zfp292	Autism
	3	587	5	Skor1	Obesity
	647	588	5	Rbms1	T2D
	156	590	5	Olig3	Obesity
	130	592	5	Cul3	Autism
	619	71	6	Fam57b	Obesity
	349	73	6	Sgcg	T2D
	236	74	6	Tnfrsf11b	T1D
	222	75	6	Ank1	T2D
	533	76	6	Zbtb7b	Obesity
	217	77	6	Ptchd1	Autism
	286	78	6	Abcc8	T2D
	41	79	6	Kcnk3	Obesity
	162	80	6	Suox	T1D
	199	82	6	Inpp5e	Obesity
	403	83	6	Tmem163	T2D
	10	95	6	Sh2b3	T1D

Supplementary Table 16 | Cont.

Color key WGCNA dendrogram	WGCNA dendrogram	Hierarchical cluster dendrogram	Module #	Gene name in heatmap	Genetic category
	436	96	6	Reln	Autism
	47	97	6	Ski	Autism
	211	98	6	Itgb6	T2D
	535	99	6	Foxo3	Obesity
	538	100	6	Arap1	T2D
	580	101	6	Cbln1	Obesity
	615	102	6	Neurod1	T2D
	485	103	6	Bcl2l15	Breast cancer
	251	104	6	Ptpn22	T1D/Breast cancer
	103	105	6	Sugp1	T2D
	175	106	6	Tm6sf2	Breast cancer
	487	107	6	Bcar1	T2D
	316	108	6	Chd7	Autism
	21	109	6	Kmt2c	Autism
	434	110	6	Lpp	T2D
	439	115	6	Zfp169	Obesity
	97	118	6	Gcc1	T2D
	239	125	6	Poc5	Obesity
	476	126	6	D5Ertd579e	Autism
	101	127	6	Pkd1	T1D
	386	144	6	Smg9	Breast cancer
	354	147	6	Syne1	Breast cancer
	518	161	6	Klhl42	T2D
	375	322	6	Alms1	Obesity
	515	323	6	Mphosph9	T2D
	623	519	6	Nbn	Breast cancer
	50	521	6	Cic	Autism
	73	522	6	Stk11	Breast cancer
	388	531	6	Zfp142	Obesity
	168	532	6	Ell	Breast cancer
	505	627	6	Plekha1	T1D
	313	633	6	Trio	Autism
	370	636	6	Bbip1	Obesity
	106	637	6	Tsc2	Autism
	233	174	7	Pam	T2D
	601	187	7	Tagap	T1D
	38	188	7	Tcerg1l	T2D
	577	192	7	Ssbp4	Breast cancer
	468	201	7	Nudt17	Breast cancer

Supplementary Table 16 | Cont.

Color key WGCNA dendrogram	WGCNA dendrogram	Hierarchical cluster dendrogram	Module #	Gene name in heatmap	Genetic category
	145	202	7	Cdk18	T1D
	421	207	7	Pcsk1	Obesity/T2D
	126	208	7	Ogfod1	Breast cancer
	266	209	7	Entpd6	Obesity
	379	210	7	Mboat7	Autism
	524	211	7	Adcy3	Obesity/breast cancer
	443	212	7	Ntrk2	Obesity
	470	213	7	Lrp1b	Obesity
	406	215	7	Bbs4	Obesity
	334	217	7	Ralyl	Obesity
	493	223	7	Nt5c2	Obesity
	184	224	7	Rhbdd3	Breast cancer
	85	225	7	Dscam	Autism
	309	226	7	Pcdh19	Autism
	25	275	7	Ap2s1	Autism
	346	277	7	Ube2e2	T2D
	16	281	7	Herc2	T1D
	512	288	7	Grp	Obesity
	323	289	7	Agbl4	Obesity
	225	290	7	Doc2a	Obesity
	170	291	7	Slc22a3	Obesity
	532	292	7	Rorb	Autism
	603	293	7	Grid1	Obesity
	563	294	7	Lingo2	Obesity
	480	295	7	Lrfn2	Obesity
	165	296	7	Chd3	Autism
	36	297	7	Pacs1	Autism
	520	298	7	Sgsm2	T2D
	52	299	7	Tanc2	Autism
	137	300	7	Celf4	Autism
	179	301	7	Nlgn3	Autism
	94	302	7	Cadm1	Obesity
	119	303	7	Jazf1	T2D
	58	526	7	Gpr151	Obesity
	351	537	7	Taf11	T2D
	226	540	7	Prkd1	Obesity
	595	642	7	Qpctl	Obesity
	264	231	8	C2cd4a	T2D
	486	232	8	Tnni3k	Obesity

Supplementary Table 16 | Cont.

Color key WGCNA dendrogram	WGCNA dendrogram	Hierarchical cluster dendrogram	Module #	Gene name in heatmap	Genetic category
	581	233	8	C2cd4b	T2D
	350	234	8	Tbr1	Autism
	409	238	8	Sec16b	Obesity
	633	239	8	Mkx	Autism
	71	240	8	Stxbp6	Obesity
	602	251	8	Srr	T2D
	393	252	8	Grin2b	Autism
	99	253	8	Syngap1	Autism
	138	254	8	Pten	Autism/Breast cancer
	465	255	8	Cdkl5	Autism
	528	256	8	Rasgrp1	T1D/T2D
	235	257	8	Lztr1	Autism
	28	258	8	Tnfaip3	T1D
	157	259	8	Coro1a	Autism
	481	261	8	Nyap2	T2D
	497	262	8	Kcnq3	Autism
	6	263	8	Kcnb1	Autism
	364	264	8	Lrrc4c	Autism
	301	265	8	Hras	Autism
	234	266	8	Snx7	T2D
	380	267	8	Myt1l	Autism
	191	268	8	Fosl2	T1D
	163	270	8	Gabrb3	Autism
	171	271	8	Dlg4	Autism
	340	272	8	Gria2	Autism
	543	273	8	Arhgef9	Autism
	381	274	8	Ppp1r9b	Autism
	153	283	8	Camk1d	T2D
	299	284	8	Cadm2	Obesity
	29	285	8	Nckap1	Autism
	182	313	8	Arrdc3	Breast cancer
	305	611	8	Cux2	T1D
	98	612	8	Satb1	Autism
	356	613	8	Wdpcp	Obesity
	457	94	9	Sema3d	Obesity
	51	194	9	Fhit	Obesity
	583	198	9	Htr1a	T1D
	551	286	9	Kctd13	Obesity
	183	309	9	Mapk14	T1D

Supplementary Table 16 | Cont.

Color key WGCNA dendrogram	WGCNA dendrogram	Hierarchical cluster dendrogram	Module #	Gene name in heatmap	Genetic category
	328	321	9	Mtch2	Obesity
	243	324	9	Cdkal1	Obesity/T2D
	307	327	9	Ep300	Autism
	292	328	9	Pms2	Breast cancer
	111	329	9	Ptpn2	T1D
	387	330	9	Sfxn2	Obesity
	550	331	9	Kdm3b	Autism
	210	332	9	Nsd1	Autism
	147	333	9	Dclre1b	Breast cancer
	604	334	9	Kat8	Obesity
	149	417	9	Usp37	Obesity
	491	419	9	Ttll4	Obesity
	539	477	9	Zfp646	Obesity
	429	478	9	Ctcf	Autism
	256	480	9	Chd8	Autism
	460	481	9	Phf12	Autism
	422	482	9	Hif1an	Obesity
	180	513	9	Tcf19	T2D
	129	528	9	Nlrc3	Obesity
	49	530	9	Rab23	Obesity
	399	157	10	Ccdc170	Breast cancer
	115	170	10	Tspan8	T2D
	330	171	10	Calcr	Obesity
	329	172	10	Gck	T2D
	634	173	10	Hnf1b	T2D
	600	175	10	Ssr1	T2D
	459	176	10	Stat3	T1D
	495	177	10	Pdx1	T2D
	372	178	10	Esr1	Breast cancer
	420	179	10	Ifng	T1D
	87	180	10	Enpp7	Breast cancer
	117	181	10	Pomc	Obesity
	40	182	10	Asb4	Obesity
	440	183	10	Katnal2	Autism
	336	186	10	Tm9sf4	Autism
	571	307	10	Srpr	Autism
	237	535	10	Stk33	Obesity
	17	538	10	Bbs10	Obesity
	172	539	10	Galnt10	Obesity
	57	116	11	Bbs2	Obesity

Supplementary Table 16 | Cont.

Color key WGCNA dendrogram	WGCNA dendrogram	Hierarchical cluster dendrogram	Module #	Gene name in heatmap	Genetic category
	196	117	11	Zbtb20	Autism
	288	119	11	Ormdl3	T1D
	394	120	11	Ttc8	Obesity
	124	122	11	Nbea	Autism
	342	123	11	Nlgn2	Autism
	65	190	11	Bbs1	Obesity
	343	191	11	Bbs7	Obesity
	203	631	11	Mecp2	Autism
	594	634	11	Sdccag8	Obesity
	442	635	11	Sh2b1	Obesity
	109	643	11	Taok2	Obesity
	279	644	11	Tsc1	Autism
	92	152	12	Ahdc1	Autism
	473	445	12	Sbk1	Obesity
	365	484	12	Irf2bpl	Autism
	599	485	12	Kansl1	Autism
	430	486	12	Setd2	Autism
	327	518	12	Bckdk	Autism
	317	520	12	Ahrr	Breast cancer
	166	523	12	Bcdin3d	T2D
	207	524	12	Zfand6	T2D
	584	529	12	Ap4b1	Breast cancer
	557	533	12	Gigyf1	Autism
	274	241	13	Lmo7	T1D
	134	242	13	Adcy5	T2D
	136	243	13	Rarb	Obesity
	39	244	13	Cacna2d3	Autism
	362	245	13	Wfs1	T2D
	300	246	13	Dgkb	T2D
	48	247	13	Mchr1	Breast cancer
	269	248	13	Abcb11	T2D
	160	249	13	Foxp1	Autism
	42	250	13	Meis2	Autism
	555	465	14	Igf2	T2D
	608	466	14	Ppp1r3b	T2D
	483	579	14	Foxp2	Autism
	613	589	14	Tnnt3	Breast cancer
	7	591	14	Tlr1	Breast cancer
	142	596	14	Lepr	Obesity
	8	597	14	Vkorc1	Obesity

Supplementary Table 16 | Cont.

Color key WGCNA dendrogram	WGCNA dendrogram	Hierarchical cluster dendrogram	Module #	Gene name in heatmap	Genetic category
	513	598	14	Igf1	T2D
	271	45	15	Slc2a2	T2D
	610	93	15	Blk	T2D
	640	151	15	Cyp17a1	Obesity
	629	156	15	Kcnn4	Breast cancer
	419	158	15	Lpar3	T1D
	287	159	15	Il2	T1D
	223	227	15	Pax4	T2D
	33	311	15	Il27	T1D
	19	312	15	Oasl1	T2D
	534	433	15	Cd69	T1D
	344	512	15	Kcnq1	T2D
	293	514	15	Foxg1	Autism
	345	567	15	Kcnk16	T2D
	310	583	15	Foxa2	T2D
	89	594	15	Ins2	T1D/T2D
	164	614	15	Ctla4	T1D
	120	615	15	Nr4a2	Autism

Supplementary Table 17 | Clustering of obesity- and T1D-associated genes. Description of each gene including: module number, gene name and disease. Relates to Fig. 13A.

Module #	Gene Name	Disease
1	Adpgk	Obesity
1	Alms1	Obesity
1	Apobr	Obesity
1	Cars	T1D
1	Cdkal1	Obesity
1	Cenpw	T1D
1	Cep295	Obesity
1	Cnot9	Obesity
1	Cobl	T1D
1	Erbb3	T1D
1	Etv5	Obesity
1	Fancl	Obesity
1	Gdf15	Obesity
1	Gtf3a	Obesity
1	Hif1an	Obesity
1	Hmga1	Obesity
1	Il10	T1D
1	Ino80e	Obesity
1	Itpr3	T1D
1	Kat8	Obesity
1	Mapk14	T1D
1	Mks1	Obesity
1	Mtch2	Obesity
1	Mtfr2	Obesity
1	Nlrc3	Obesity
1	Nup54	Obesity
1	Pgm1	T1D
1	Polk	Obesity
1	Ptpn2	T1D
1	Rab23	Obesity
1	Rcc1l	Obesity
1	Rpl27a	Obesity
1	Sfxn2	Obesity
1	Skap2	T1D
1	Snrpd2	Obesity
1	Stat3	T1D
1	Stat4	T1D
1	Tomm40	Obesity
1	Ttll4	Obesity
1	Tyk2	T1D

Supplementary Table 17 | Cont.

Module #	Gene Name	Disease
1	Ubash3a	T1D
1	Usp37	Obesity
1	Zfp64	Obesity
1	Zfp646	Obesity
2	Asb4	Obesity
2	Bach2	T1D
2	Bbs10	Obesity
2	Bbs12	Obesity
2	Bbs9	Obesity
2	C1qtnf6	T1D
2	Cd69	T1D
2	Creb1	Obesity
2	Dlk1	T1D
2	Ehbp1	Obesity
2	Elavl4	Obesity
2	Fbxl19	Obesity
2	Fign	Obesity
2	Frmd3	T1D
2	Galnt10	Obesity
2	Gipr	Obesity
2	Glis3	T1D
2	Hip1	Obesity
2	Hmgcr	Obesity
2	Ift27	Obesity
2	Ift74	Obesity
2	Ikzf4	T1D
2	Ins2	T1D
2	Irs1	Obesity
2	Kctd15	Obesity
2	Klf7	Obesity
2	Lepr	Obesity
2	Lmo1	Obesity
2	Lmx1b	Obesity
2	Nav1	Obesity
2	Nrp1	Obesity
2	Nrp2	Obesity
2	Olig3	Obesity
2	Pbrm1	Obesity
2	Pgpep1	Obesity
2	Phip	Obesity
2	Plxna1	Obesity

Supplementary Table 17 | Cont.

Module #	Gene Name	Disease
2	Plxna2	Obesity
2	Plxna3	Obesity
2	Ppil2	T1D
2	Prkd2	T1D
2	Ptbp2	Obesity
2	Rnls	T1D
2	Rpgrip1l	Obesity
2	Sbk1	Obesity
2	Sh2b1	Obesity
2	Sim1	Obesity
2	Skor1	Obesity
2	Slc39a8	Obesity
2	Smg6	Obesity
2	Stk33	Obesity
2	Stk36	Obesity
2	Tal1	Obesity
2	Tcf7l2	Obesity/T1D
2	Tfap2b	Obesity
2	Txndc12	Obesity
2	Ube2e3	Obesity
2	Zbtb10	Obesity
2	Zfhx3	Obesity
2	Zfp608	Obesity
2	Zfp668	Obesity
3	Adcy3	Obesity
3	Agbl4	Obesity
3	Arl6	Obesity
3	Bbs1	Obesity
3	Bbs2	Obesity
3	Bbs4	Obesity
3	Bbs5	Obesity
3	Bbs7	Obesity
3	Bdnf	Obesity
3	Cadm1	Obesity
3	Cadm2	Obesity
3	Calcr	Obesity
3	Cdk18	T1D
3	Ctla4	T1D
3	Cux2	T1D
3	Cyp17a1	Obesity
3	Doc2a	Obesity

Supplementary Table 17 | Cont.

Module #	Gene Name	Disease
3	Entpd6	Obesity
3	Erbb4	Obesity
3	Ets2	Obesity
3	Fhit	Obesity
3	Fosl2	T1D
3	Gpr151	Obesity
3	Grid1	Obesity
3	Grp	Obesity
3	Herc2	T1D
3	Htr1a	T1D
3	Ifng	T1D
3	Il2	T1D
3	Kctd13	Obesity
3	Kif5a	T1D
3	Lingo2	Obesity
3	Lmo7	T1D
3	Lrfn2	Obesity
3	Lrp1b	Obesity
3	Lztfl1	Obesity
3	Map2k5	Obesity
3	Mapk3	Obesity
3	Mc4r	Obesity
3	Negr1	Obesity
3	Nt5c2	Obesity
3	Ntrk2	Obesity
3	Nudt3	Obesity
3	Ormdl3	T1D
3	Park2	Obesity
3	Pcsk1	Obesity
3	Phtf1	T1D
3	Pip4k2c	T1D
3	Plxna4	Obesity
3	Pomc	Obesity
3	Prkd1	Obesity
3	Qpctl	Obesity
3	Rabep1	Obesity
3	Ralyl	Obesity
3	Rarb	Obesity
3	Rasa2	Obesity
3	Rasgrp1	T1D
3	Sdccag8	Obesity

Supplementary Table 17 | Cont.

Module #	Gene Name	Disease
3	Sec16b	Obesity
3	Sema3c	Obesity
3	Slc22a3	Obesity
3	Stxbp6	Obesity
3	Tagap	T1D
3	Taok2	Obesity
3	Tnfaip3	T1D
3	Tnni3k	Obesity
3	Trim32	Obesity
3	Trim66	Obesity
3	Ttc8	Obesity
3	Tub	Obesity
3	Vkorc1	Obesity
3	Wdpcp	Obesity
3	Zfr2	Obesity
4	Ache	Obesity
4	Ankdd1b	Obesity
4	Bbip1	Obesity
4	Cbln1	Obesity
4	Cep290	Obesity
4	Clec16a	T1D
4	Cobll1	Obesity
4	Col4a3bp	Obesity
4	Ctsh	T1D
4	Cyp27a1	Obesity
4	Dmxl2	Obesity
4	Dnajc27	Obesity
4	Efr3b	Obesity/T1D
4	Epb41l4b	Obesity
4	Erap1	T1D
4	Faim2	Obesity
4	Fam189a2	T1D
4	Fam57b	Obesity
4	Foxo3	Obesity
4	Frrs1l	Obesity
4	Fto	Obesity
4	Gbe1	Obesity
4	Gnpda2	Obesity
4	Gprc5b	Obesity
4	Hhip	Obesity
4	Hsd17b12	Obesity

Supplementary Table 17 | Cont.

Module #	Gene Name	Disease
4	Iars2	Obesity
4	Ifih1	T1D
4	Ifngr1	Obesity
4	Il27	T1D
4	Il2ra	T1D
4	Inpp5e	Obesity
4	Kcnj11	T1D
4	Kcnk3	Obesity
4	Kcnk9	Obesity
4	Ksr2	Obesity
4	Lpar3	T1D
4	Mkks	Obesity
4	Mtif3	Obesity
4	Nrxn3	Obesity
4	Pcdh9	Obesity
4	Pex2	Obesity
4	Pkd1	T1D
4	Plcd4	Obesity
4	Plekha1	T1D
4	Poc5	Obesity
4	Pparg	T1D
4	Prkcq	T1D
4	Ptpn22	T1D
4	Rab21	Obesity
4	Rapgef3	Obesity
4	Ric3	Obesity
4	Rit2	Obesity
4	Rnaset2a	T1D
4	Scarb2	Obesity
4	Scg3	Obesity
4	Sema3a	Obesity
4	Sema3b	Obesity
4	Sema3d	Obesity
4	Sh2b3	T1D
4	Ssfa2	Obesity
4	Stx1b	Obesity
4	Suox	T1D
4	Tlr4	Obesity
4	Tmem160	Obesity
4	Tmem18	Obesity
4	Tnfrsf11b	T1D

Supplementary Table 17 | Cont.

Module #	Gene Name	Disease
4	Ypel3	Obesity
4	Zbtb7b	Obesity
4	Zfp142	Obesity
4	Zfp169	Obesity

Supplementary Table 18 | Clustering of obesity- and T2D-associated genes. Description of each gene including: module number, gene name and disease. Relates to Fig. 13B.

Module #	Gene Name	Disease
1	Abcb11	T2D
1	Ache	Obesity
1	Adcy3	Obesity
1	Adcy5	T2D
1	Adra2a	T2D
1	Agbl4	Obesity
1	Ankrd55	T2D
1	Arl6	Obesity
1	Bbs1	Obesity
1	Bbs2	Obesity
1	Bbs4	Obesity
1	Bbs5	Obesity
1	Bbs7	Obesity
1	Bdnf	Obesity
1	C2cd4a	T2D
1	C2cd4b	T2D
1	Cadm1	Obesity
1	Cadm2	Obesity
1	Calcr	Obesity
1	Camk1d	T2D
1	Cdc123	T2D
1	Col4a3bp	Obesity
1	Cry2	T2D
1	Dgkb	T2D
1	Dnajc27	Obesity
1	Doc2a	Obesity
1	Entpd6	Obesity
1	Erbb4	Obesity
1	Ets2	Obesity
1	Fads1	T2D
1	Faim2	Obesity/T2D
1	Fhit	Obesity
1	Fto	Obesity/T2D
1	Gbe1	Obesity
1	Gck	T2D
1	Gpr151	Obesity
1	Grb14	T2D
1	Grid1	Obesity
1	Grp	Obesity
1	Hectd4	T2D

Supplementary Table 18 | Cont.

Module #	Gene Name	Disease
1	Hnf1b	T2D
1	Ifngr1	Obesity
1	Jazf1	T2D
1	Kcnk9	Obesity
1	Kctd13	Obesity
1	Lingo2	Obesity
1	Lrfn2	Obesity
1	Lrp1b	Obesity
1	Lztfl1	Obesity
1	Maea	T2D
1	Mapk3	Obesity
1	Mc4r	Obesity/T2D
1	Msmo1	T2D
1	Negr1	Obesity
1	Nt5c2	Obesity
1	Ntrk2	Obesity
1	Nyap2	T2D
1	Pam	T2D
1	Park2	Obesity
1	Pcsk1	Obesity/T2D
1	Plxna4	Obesity
1	Pomc	Obesity
1	Prkd1	Obesity
1	Ptprd	T2D
1	Qpctl	Obesity
1	Rabep1	Obesity
1	Ralyl	Obesity
1	Rapgef3	Obesity
1	Rarb	Obesity
1	Rasa2	Obesity
1	Rasgrp1	T2D
1	Rit2	Obesity
1	Scarb2	Obesity
1	Sdhaf4	T2D
1	Sec16b	Obesity
1	Sema3c	Obesity
1	Sema3d	Obesity
1	Sgsm2	T2D
1	Slc22a3	Obesity
1	Slc2a2	T2D
1	Slc30a8	T2D

Supplementary Table 18 | Cont.

Module #	Gene Name	Disease
1	Snx7	T2D
1	Srr	T2D
1	Stxbp6	Obesity
1	Taf11	T2D
1	Taok2	Obesity
1	Tbc1d30	T2D
1	Tcerg1l	T2D
1	Tnni3k	Obesity
1	Trim32	Obesity
1	Trim66	Obesity
1	Ttc8	Obesity
1	Tub	Obesity
1	Ube2e2	T2D
1	Vkorc1	Obesity
1	Wfs1	T2D
1	Zfr2	Obesity
2	Asb4	Obesity
2	Bbs10	Obesity
2	Bbs12	Obesity
2	Bbs9	Obesity
2	Bcdin3d	T2D
2	Bcl11a	T2D
2	Capn10	T2D
2	Ccnd2	T2D
2	Cel	T2D
2	Creb1	Obesity
2	Dpysl5	T2D
2	Ehbp1	Obesity
2	Elavl4	Obesity
2	Fbxl19	Obesity
2	Fign	Obesity
2	Foxa2	T2D
2	Galnt10	Obesity
2	Gipr	Obesity/T2D
2	Glis3	T2D
2	Gpsm1	T2D
2	Grb10	T2D
2	Hip1	Obesity
2	Hmg20a	T2D
2	Hmgcr	Obesity
2	Ift27	Obesity

Supplementary Table 18 | Cont.

Module #	Gene Name	Disease
2	Ift74	Obesity
2	Igf1	T2D
2	Igf2	T2D
2	Ins2	T2D
2	Irs1	Obesity/T2D
2	Kcnk16	T2D
2	Kcnq1	T2D
2	Kctd15	Obesity
2	Klf14	T2D
2	Klf7	Obesity
2	Lepr	Obesity
2	Lmo1	Obesity
2	Lmx1b	Obesity
2	Macf1	T2D
2	Nav1	Obesity
2	Notch2	T2D
2	Nrp1	Obesity
2	Nrp2	Obesity
2	Nudt3	Obesity
2	Olig3	Obesity
2	Pbrm1	Obesity
2	Pgpep1	Obesity
2	Phip	Obesity
2	Plxna1	Obesity
2	Plxna2	Obesity
2	Plxna3	Obesity
2	Ppip5k2	T2D
2	Ppp1r3b	T2D
2	Prox1	T2D
2	Ptbp2	Obesity
2	Rbms1	T2D
2	Rnd3	T2D
2	Rpgrip1l	Obesity
2	Sbk1	Obesity
2	Sdccag8	Obesity
2	Sh2b1	Obesity
2	Sim1	Obesity
2	Skor1	Obesity
2	Slc39a8	Obesity
2	Smg6	Obesity
2	St6gal1	T2D

Supplementary Table 18 | Cont.

Module #	Gene Name	Disease
2	Stk33	Obesity
2	Stk36	Obesity
2	Tal1	Obesity
2	Tcf7l2	Obesity/T2D
2	Tfap2b	Obesity
2	Tle1	T2D
2	Txndc12	Obesity
2	Ube2e3	Obesity
2	Wdpcp	Obesity
2	Zbed3	T2D
2	Zfand3	T2D
2	Zfand6	T2D
2	Zfhx3	Obesity
2	Zfp608	Obesity
2	Zfp668	Obesity
2	Zmiz1	T2D
3	Abcc8	T2D
3	Agmo	T2D
3	Ank1	T2D
3	Ankdd1b	Obesity
3	Appl1	T2D
3	Arap1	T2D
3	Atg13	T2D
3	Bbip1	Obesity
3	Bcar1	T2D
3	Blk	T2D
3	Cbln1	Obesity
3	Cep290	Obesity
3	Cobll1	Obesity/T2D
3	Cyp17a1	Obesity
3	Cyp27a1	Obesity
3	Dmxl2	Obesity
3	Efr3b	Obesity
3	Epb41l4b	Obesity
3	Fam57b	Obesity
3	Fitm2	T2D
3	Foxo3	Obesity
3	Frrs1l	Obesity
3	Gcc1	T2D
3	Gckr	T2D
3	Gnpda2	Obesity

Supplementary Table 18 | Cont.

Module #	Gene Name	Disease
3	Gprc5b	Obesity
3	Grk5	T2D
3	Hhip	Obesity
3	Hsd17b12	Obesity
3	Iars2	Obesity
3	Inpp5e	Obesity
3	Itgb6	T2D
3	Kank1	T2D
3	Kcnj11	T2D
3	Kcnk3	Obesity
3	Klhl42	T2D
3	Ksr2	Obesity
3	Larp6	T2D
3	Lgr5	T2D
3	Lpp	T2D
3	Madd	T2D
3	Map2k5	Obesity
3	Mkks	Obesity
3	Mtif3	Obesity
3	Neurod1	T2D
3	Nrxn3	Obesity
3	Pcdh9	Obesity
3	Pex2	Obesity
3	Plcd4	Obesity
3	Poc5	Obesity
3	Pparg	T2D
3	Rab21	Obesity
3	Ric3	Obesity
3	Scg3	Obesity
3	Sema3a	Obesity
3	Sema3b	Obesity
3	Sgcg	T2D
3	Ssfa2	Obesity
3	Stx1b	Obesity
3	Sugp1	T2D
3	Tlr4	Obesity
3	Tmem160	Obesity
3	Tmem163	T2D
3	Tmem18	Obesity
3	Vps13c	T2D
3	Ypel3	Obesity

Supplementary Table 18 | Cont.

Module #	Gene Name	Disease
3	Zbtb7b	Obesity
3	Zfp142	Obesity
3	Zfp169	Obesity
4	Abo	T2D
4	Adamts9	T2D
4	Adpgk	Obesity
4	Alms1	Obesity
4	Ap3s2	T2D
4	Apobr	Obesity
4	Arl15	T2D
4	Cdkal1	Obesity/T2D
4	Cdkn2a	T2D
4	Cdkn2b	T2D
4	Cep295	Obesity
4	Cilp2	T2D
4	Cnot9	Obesity
4	Dusp9	T2D
4	Etv5	Obesity
4	Faf1	T2D
4	Fancl	Obesity
4	G6pc2	T2D
4	Gatad2a	T2D
4	Gdf15	Obesity
4	Gtf3a	Obesity
4	Hhex	T2D
4	Hif1an	Obesity
4	Hmga1	Obesity
4	Hmga2	T2D
4	Hnf1a	T2D
4	Hnf4a	T2D
4	Ide	T2D
4	Igf2bp2	T2D
4	Ino80e	Obesity
4	Kat8	Obesity
4	Klf11	T2D
4	Lama1	T2D
4	Lyplal1	T2D
4	Mks1	Obesity
4	Mphosph9	T2D
4	Mtch2	Obesity
4	Mtfr2	Obesity

Supplementary Table 18 | Cont.

Module #	Gene Name	Disease
4	Nlrc3	Obesity
4	Nup54	Obesity
4	Oasl1	T2D
4	Pax4	T2D
4	Pdgfc	T2D
4	Pdx1	T2D
4	Pepd	T2D
4	Polk	Obesity
4	Pou5f1	T2D
4	Prc1	T2D
4	Psmd6	T2D
4	R3hdml	T2D
4	Rab23	Obesity
4	Rcc1l	Obesity
4	Rpl27a	Obesity
4	Rreb1	T2D
4	Sfxn2	Obesity
4	Snrpd2	Obesity
4	Spry2	T2D
4	Srsf2	T2D
4	Ssr1	T2D
4	Tcf19	T2D
4	Tcf7	T2D
4	Thada	T2D
4	Tle4	T2D
4	Tomm40	Obesity
4	Trp53inp1	T2D
4	Tspan8	T2D
4	Ttll4	Obesity
4	Usp37	Obesity
4	Vps26a	T2D
4	Zbtb10	Obesity
4	Zfp64	Obesity
4	Zfp646	Obesity

Supplementary Table 19 | Clustering of obesity- and autism-associated genes. Description of each gene including: module number, gene name and disease. Relates to Fig. 13C.

Module #	Gene Name	Disease
1	Adpgk	Obesity
1	Adsl	Autism
1	Alms1	Obesity
1	Apobr	Obesity
1	Atrx	Autism
1	Cdkal1	Obesity
1	Cep295	Obesity
1	Chd8	Autism
1	Cnot3	Autism
1	Cnot9	Obesity
1	Ctcf	Autism
1	Ddx3x	Autism
1	Dhcr7	Autism
1	Dnmt3a	Autism
1	Eif3g	Autism
1	Ep300	Autism
1	Etv5	Obesity
1	Fancl	Obesity
1	Fmr1	Autism
1	Gdf15	Obesity
1	Gigyf1	Autism
1	Gigyf2	Autism
1	Gtf3a	Obesity
1	Hdlbp	Autism
1	Hif1an	Obesity
1	Hmga1	Obesity
1	Hnrnpu	Autism
1	Ino80e	Obesity
1	Kat8	Obesity
1	Kdm3b	Autism
1	Kdm5b	Autism
1	Med13l	Autism
1	Mks1	Obesity
1	Mtch2	Obesity
1	Mtfr2	Obesity
1	Naa15	Autism
1	Nacc1	Autism
1	Nipbl	Autism
1	Nlrc3	Obesity

Supplementary Table 19 | Cont.

Module #	Gene Name	Disease
1	Nsd1	Autism
1	Nup155	Autism
1	Nup54	Obesity
1	Phf12	Autism
1	Phf3	Autism
1	Polk	Obesity
1	Psmd12	Autism
1	Ptk7	Autism
1	Rab23	Obesity
1	Rcc1l	Obesity
1	Rpl27a	Obesity
1	Setd5	Autism
1	Sfxn2	Obesity
1	Sin3a	Autism
1	Snrpd2	Obesity
1	Srpr	Autism
1	Tbl1xr1	Autism
1	Tcf20	Autism
1	Tomm40	Obesity
1	Traf7	Autism
1	Trip12	Autism
1	Ttll4	Obesity
1	Usp37	Obesity
1	Zbtb10	Obesity
1	Zfp142	Obesity
1	Zfp64	Obesity
1	Zfp646	Obesity
1	Zmynd8	Autism
2	Ache	Obesity
2	Aldh5a1	Autism
2	Ank2	Autism
2	Ank3	Autism
2	Ankdd1b	Obesity
2	Ankrd11	Autism
2	Cbln1	Obesity
2	Cep290	Obesity
2	Cobll1	Obesity
2	Col4a3bp	Obesity
2	Cyp27a1	Obesity
2	D5Ertd579e	Autism
2	Dip2a	Autism

Supplementary Table 19 | Cont.

Module #	Gene Name	Disease
2	Dnajc27	Obesity
2	Efr3b	Obesity
2	Epb41l4b	Obesity
2	Faim2	Obesity
2	Fam57b	Obesity
2	Fhit	Obesity
2	Foxo3	Obesity
2	Frrs1l	Obesity
2	Fto	Obesity
2	Gabrb2	Autism
2	Gbe1	Obesity
2	Gfap	Autism
2	Gnai1	Autism
2	Gnpda2	Obesity
2	Gprc5b	Obesity
2	Hhip	Obesity
2	Hsd17b12	Obesity
2	Iars2	Obesity
2	Ifngr1	Obesity
2	Inpp5e	Obesity
2	Kcnk3	Obesity
2	Kcnk9	Obesity
2	Map1a	Autism
2	Mkks	Obesity
2	Mtif3	Obesity
2	Nrxn3	Obesity/Autism
2	Park2	Obesity
2	Pcdh9	Obesity
2	Pex2	Obesity
2	Plcd4	Obesity
2	Poc5	Obesity
2	Ppp5c	Autism
2	Ptchd1	Autism
2	Ptpn11	Autism
2	Rab21	Obesity
2	Rapgef3	Obesity
2	Reln	Autism
2	Ric3	Obesity
2	Rit2	Obesity
2	Scarb2	Obesity
2	Scg3	Obesity

Supplementary Table 19 | Cont.

Module #	Gene Name	Disease
2	Scn1a	Autism
2	Scn8a	Autism
2	Sema3b	Obesity
2	Sema3d	Obesity
2	Ski	Autism
2	Slc6a1	Autism
2	Ssfa2	Obesity
2	Stx1b	Obesity
2	Stxbp1	Autism
2	Tek	Autism
2	Tlr4	Obesity
2	Tmem160	Obesity
2	Tmem18	Obesity
2	Trim66	Obesity
2	Ubr1	Autism
2	Vkorc1	Obesity
2	Vps13b	Autism
2	Wdfy3	Autism
2	Ypel3	Obesity
2	Zbtb7b	Obesity
2	Zfp169	Obesity
3	Adnp	Autism
3	Aff2	Autism
3	Arid1b	Autism
3	Arx	Autism
3	Asb4	Obesity
3	Asxl3	Autism
3	Auts2	Autism
3	Baz2b	Autism
3	Bbip1	Obesity
3	Bbs10	Obesity
3	Bbs12	Obesity
3	Bbs9	Obesity
3	Bckdk	Autism
3	Bcl11a	Autism
3	Cask	Autism
3	Champ1	Autism
3	Chd2	Autism
3	Chd7	Autism
3	Cic	Autism
3	Creb1	Obesity

Supplementary Table 19 | Cont.

Module #	Gene Name	Disease
3	Crebbp	Autism
3	Ctnnb1	Autism
3	Cul3	Autism
3	Deaf1	Autism
3	Dmpk	Autism
3	Dync1h1	Autism
3	Dyrk1a	Autism
3	Ebf3	Autism
3	Ehbp1	Obesity
3	Ehmt1	Autism
3	Elavl3	Autism
3	Elavl4	Obesity
3	Fbxl19	Obesity
3	Fign	Obesity
3	Foxg1	Autism
3	Foxp2	Autism
3	Galnt10	Obesity
3	Gipr	Obesity
3	Hip1	Obesity
3	Hmgcr	Obesity
3	Ift27	Obesity
3	Ift74	Obesity
3	Irf2bpl	Autism
3	Irs1	Obesity
3	Kansl1	Autism
3	Katnal2	Autism
3	Kctd15	Obesity
3	Kdm6b	Autism
3	Klf7	Obesity
3	Kmt2a	Autism
3	Kmt2c	Autism
3	Kmt2e	Autism
3	Kmt5b	Autism
3	Ldb1	Autism
3	Lepr	Obesity
3	Lmo1	Obesity
3	Lmx1b	Obesity
3	Magel2	Autism
3	Med13	Autism
3	Meis2	Autism
3	Nav1	Obesity

Supplementary Table 19 | Cont.

Module #	Gene Name	Disease
3	Nrp1	Obesity
3	Nrp2	Obesity
3	Olig3	Obesity
3	Pax5	Autism
3	Pbrm1	Obesity
3	Pgpep1	Obesity
3	Phf2	Autism
3	Phf21a	Autism
3	Phip	Obesity/Autism
3	Plxna1	Obesity
3	Plxna2	Obesity
3	Plxna3	Obesity
3	Pogz	Autism
3	Prr12	Autism
3	Ptbp2	Obesity
3	Rai1	Autism
3	Rere	Autism
3	Rfx3	Autism
3	Rpgrip1l	Obesity
3	Satb1	Autism
3	Sbk1	Obesity
3	Sema3a	Obesity
3	Setbp1	Autism
3	Setd2	Autism
3	Sh2b1	Obesity
3	Sim1	Obesity
3	Skor1	Obesity
3	Slc39a8	Obesity
3	Smarcc2	Autism
3	Smg6	Obesity
3	Sox5	Autism
3	Spast	Autism
3	Srcap	Autism
3	Stk33	Obesity
3	Stk36	Obesity
3	Tal1	Obesity
3	Tbck	Autism
3	Tcf7l2	Obesity/Autism
3	Tfap2b	Obesity
3	Tlk2	Autism
3	Trio	Autism

Supplementary Table 19 | Cont.

Module #	Gene Name	Disease
3	Tsc2	Autism
3	Tshz3	Autism
3	Txndc12	Obesity
3	Ube2e3	Obesity
3	Upf3b	Autism
3	Vezf1	Autism
3	Wac	Autism
3	Zfhx3	Obesity
3	Zfp292	Autism
3	Zfp462	Autism
3	Zfp608	Obesity
3	Zfp668	Obesity
4	Adcy3	Obesity
4	Agbl4	Obesity
4	Ahdc1	Autism
4	Ap2s1	Autism
4	Arhgef9	Autism
4	Arl6	Obesity
4	Ash1l	Autism
4	Bbs1	Obesity
4	Bbs2	Obesity
4	Bbs4	Obesity
4	Bbs5	Obesity
4	Bbs7	Obesity
4	Bdnf	Obesity
4	Braf	Autism
4	Brsk2	Autism
4	Cacna1c	Autism
4	Cacna1e	Autism
4	Cacna2d3	Autism
4	Cadm1	Obesity
4	Cadm2	Obesity
4	Calcr	Obesity
4	Cdkl5	Autism
4	Celf4	Autism
4	Chd3	Autism
4	Coro1a	Autism
4	Cyp17a1	Obesity
4	Dlg4	Autism
4	Dmxl2	Obesity
4	Doc2a	Obesity

Supplementary Table 19 | Cont.

Module #	Gene Name	Disease
4	Dpysl2	Autism
4	Dscam	Autism
4	Entpd6	Obesity
4	Erbb4	Obesity
4	Ets2	Obesity
4	Foxp1	Autism
4	Gabrb3	Autism
4	Gpr151	Obesity
4	Gria2	Autism
4	Grid1	Obesity
4	Grin2b	Autism
4	Grp	Obesity
4	Hectd4	Autism
4	Hivep2	Autism
4	Hnrnph2	Autism
4	Hras	Autism
4	Iqsec2	Autism
4	Kcnb1	Autism
4	Kcnq3	Autism
4	Kctd13	Obesity
4	Ksr2	Obesity
4	Lingo2	Obesity
4	Lrfn2	Obesity
4	Lrp1b	Obesity
4	Lrrc4c	Autism
4	Lztfl1	Obesity
4	Lztr1	Autism
4	Map2k5	Obesity
4	Mapk3	Obesity
4	Mbd5	Autism
4	Mboat7	Autism
4	Mc4r	Obesity
4	Mecp2	Autism
4	Mkx	Autism
4	Myt1l	Autism
4	Nbea	Autism
4	Nckap1	Autism
4	Ncoa1	Autism
4	Negr1	Obesity
4	Nf1	Autism
4	Nlgn2	Autism

Supplementary Table 19 | Cont.

Module #	Gene Name	Disease
4	Nlgn3	Autism
4	Nr3c2	Autism
4	Nr4a2	Autism
4	Nrxn1	Autism
4	Nrxn2	Autism
4	Nt5c2	Obesity
4	Ntrk2	Obesity
4	Nudt3	Obesity
4	Pacs1	Autism
4	Pcdh19	Autism
4	Pcsk1	Obesity
4	Plxna4	Obesity
4	Pomc	Obesity
4	Pomgnt1	Autism
4	Ppp1r9b	Autism
4	Ppp2r5d	Autism
4	Prkd1	Obesity
4	Pten	Autism
4	Qpctl	Obesity
4	Rabep1	Obesity
4	Ralyl	Obesity
4	Rarb	Obesity
4	Rasa2	Obesity
4	Rims1	Autism
4	Rorb	Autism
4	Scn2a	Autism
4	Sdccag8	Obesity
4	Sec16b	Obesity
4	Sema3c	Obesity
4	Shank2	Autism
4	Shank3	Autism
4	Slc22a3	Obesity
4	Slc9a6	Autism
4	Stxbp6	Obesity
4	Syngap1	Autism
4	Tanc2	Autism
4	Taok1	Autism
4	Taok2	Obesity
4	Tbr1	Autism
4	Tcf4	Autism
4	Tm9sf4	Autism

Supplementary Table 19 | Cont.

Module #	Gene Name	Disease
4	Tnni3k	Obesity
4	Trim23	Autism
4	Trim32	Obesity
4	Tsc1	Autism
4	Ttc8	Obesity
4	Tub	Obesity
4	Ube3a	Autism
4	Wdpcp	Obesity
4	Zbtb20	Autism
4	Zfr2	Obesity

Supplementary Table 20 | Clustering of obesity- and breast cancer-associated genes. Description of each gene including: module number, gene name and disease. Relates to Fig. 13D.

Module #	Gene Name	Disease
1	Abraxas1	Breast cancer
1	Adpgk	Obesity
1	Alms1	Obesity
1	Apobr	Obesity
1	Arrdc3	Breast cancer
1	Ascc2	Breast cancer
1	Atf7ip	Breast cancer
1	Atm	Breast cancer
1	Bard1	Breast cancer
1	Brca1	Breast cancer
1	Brca2	Breast cancer
1	Brip1	Breast cancer
1	Ccne1	Breast cancer
1	Cdca7l	Breast cancer
1	Cdh1	Breast cancer
1	Cdkal1	Obesity
1	Cenpo	Breast cancer
1	Cep295	Obesity
1	Chek2	Breast cancer
1	Cnot9	Obesity
1	Cul5	Breast cancer
1	Dclre1b	Breast cancer
1	Ell	Breast cancer
1	Epcam	Breast cancer
1	Etv5	Obesity
1	Fancl	Obesity
1	Gatad2a	Breast cancer
1	Gdf15	Obesity
1	Gtf3a	Obesity
1	Hif1an	Obesity
1	Hmga1	Obesity
1	Hspa4	Breast cancer
1	Ino80e	Obesity
1	Kat8	Obesity
1	Mdm4	Breast cancer
1	Mks1	Obesity
1	Mlh1	Breast cancer
1	Mrps18c	Breast cancer
1	Msh2	Breast cancer

Supplementary Table 20 | Cont.

Module #	Gene Name	Disease
1	Msh6	Breast cancer
1	Mtch2	Obesity
1	Mtfr2	Obesity
1	Nbn	Breast cancer
1	Nlrc3	Obesity
1	Npat	Breast cancer
1	Nup54	Obesity
1	Palb2	Breast cancer
1	Pidd1	Breast cancer
1	Pms2	Breast cancer
1	Polk	Obesity
1	Ppil3	Breast cancer
1	Prc1	Breast cancer
1	Rab23	Obesity
1	Rad51c	Breast cancer
1	Ralb	Breast cancer
1	Rcc1l	Obesity
1	Rpl27a	Obesity
1	Rps23	Breast cancer
1	Sfxn2	Obesity
1	Siva1	Breast cancer
1	Snrpd2	Obesity
1	Tefm	Breast cancer
1	Tomm40	Obesity
1	Trp53	Breast cancer
1	Ttll4	Obesity
1	Usp37	Obesity
1	Wdr43	Breast cancer
1	Zbtb10	Obesity
1	Zfp64	Obesity
1	Zfp646	Obesity
2	Ahrr	Breast cancer
2	Akap9	Breast cancer
2	Ap4b1	Breast cancer
2	Asb4	Obesity
2	Bbip1	Obesity
2	Bbs10	Obesity
2	Bbs12	Obesity
2	Bbs9	Obesity
2	Casp8	Breast cancer
2	Cfl1	Breast cancer

Supplementary Table 20 | Cont.

Module #	Gene Name	Disease
2	Creb1	Obesity
2	Cyp51	Breast cancer
2	Dda1	Breast cancer
2	Efemp2	Breast cancer
2	Ehbp1	Obesity
2	Elavl4	Obesity
2	Fbxl19	Obesity
2	Fign	Obesity
2	Galnt10	Obesity
2	Gipr	Obesity
2	Hip1	Obesity
2	Hmgcr	Obesity
2	Ift27	Obesity
2	Ift74	Obesity
2	Irs1	Obesity
2	Kctd15	Obesity
2	Klf7	Obesity
2	L3mbtl3	Breast cancer
2	Lepr	Obesity
2	Lmo1	Obesity
2	Lmx1b	Obesity
2	Lrrc25	Breast cancer
2	Lrrd1	Breast cancer
2	Nav1	Obesity
2	Nol12	Breast cancer
2	Nrp1	Obesity
2	Nrp2	Obesity
2	Nudt3	Obesity
2	Olig3	Obesity
2	Pbrm1	Obesity
2	Pex2	Obesity
2	Pgpep1	Obesity/Breast cancer
2	Phip	Obesity
2	Pik3c2b	Breast cancer
2	Plxna1	Obesity
2	Plxna2	Obesity
2	Plxna3	Obesity
2	Ptbp2	Obesity
2	Rccd1	Breast cancer
2	Rpgrip1l	Obesity
2	Sbk1	Obesity

Supplementary Table 20 | Cont.

Module #	Gene Name	Disease
2	Sdccag8	Obesity
2	Sec16b	Obesity
2	Sema3a	Obesity
2	Sh2b1	Obesity
2	Sim1	Obesity
2	Skor1	Obesity
2	Slc39a8	Obesity
2	Smg6	Obesity
2	Stk11	Breast cancer
2	Stk33	Obesity
2	Stk36	Obesity
2	Tal1	Obesity
2	Tcf7l2	Obesity
2	Tfap2b	Obesity
2	Tgfbr2	Breast cancer
2	Tlr1	Breast cancer
2	Tm6sf2	Breast cancer
2	Tnnt3	Breast cancer
2	Txndc12	Obesity
2	Ube2e3	Obesity
2	Wdpcp	Obesity
2	Zfhx3	Obesity
2	Zfp608	Obesity
2	Zfp668	Obesity
2	Zfp703	Breast cancer
3	Amfr	Breast cancer
3	Ankdd1b	Obesity
3	Atp6ap1l	Breast cancer
3	Bcl2l15	Breast cancer
3	Cbln1	Obesity
3	Ccdc127	Breast cancer
3	Ccdc91	Breast cancer
3	Cend1	Breast cancer
3	Cep290	Obesity
3	Cobll1	Obesity
3	Col4a3bp	Obesity
3	Coq5	Breast cancer
3	Cox11	Breast cancer
3	Cyp17a1	Obesity
3	Cyp27a1	Obesity
3	Dffa	Breast cancer

Supplementary Table 20 | Cont.

Module #	Gene Name	Disease
3	Dmxl2	Obesity
3	Dnajc27	Obesity/Breast cancer
3	Efr3b	Obesity
3	Epb41l4b	Obesity
3	Faim2	Obesity
3	Fam57b	Obesity
3	Foxo3	Obesity
3	Frrs1l	Obesity
3	Gbe1	Obesity
3	Gnpda2	Obesity
3	Gprc5b	Obesity
3	Hapln4	Breast cancer
3	Hhip	Obesity
3	Hsd17b12	Obesity
3	Iars2	Obesity
3	Inpp5e	Obesity
3	Kcnk3	Obesity
3	Kcnk9	Obesity
3	Ksr2	Obesity
3	Map2k5	Obesity
3	Mkks	Obesity
3	Mtif3	Obesity
3	Nrxn3	Obesity
3	Pcdh9	Obesity
3	Pex14	Breast cancer
3	Plcd4	Obesity
3	Poc5	Obesity
3	Ptpn22	Breast cancer
3	Rab21	Obesity
3	Rad51d	Breast cancer
3	Ric3	Obesity
3	Rit2	Obesity
3	Rnf115	Breast cancer
3	Scarb2	Obesity
3	Scg3	Obesity
3	Sdha	Breast cancer
3	Sema3b	Obesity
3	Sirt5	Breast cancer
3	Smg9	Breast cancer
3	Ssfa2	Obesity
3	Stx1b	Obesity

Supplementary Table 20 | Cont.

Module #	Gene Name	Disease
3	Syne1	Breast cancer
3	Tlr4	Obesity
3	Tmem160	Obesity
3	Tmem18	Obesity
3	Tmem184b	Breast cancer
3	Tns1	Breast cancer
3	Ypel3	Obesity
3	Zbtb38	Breast cancer
3	Zbtb7b	Obesity
3	Zfp142	Obesity
3	Zfp169	Obesity
4	Ache	Obesity
4	Adcy3	Obesity/Breast cancer
4	Agbl4	Obesity
4	Arl6	Obesity
4	Atg10	Breast cancer
4	Bbs1	Obesity
4	Bbs2	Obesity
4	Bbs4	Obesity
4	Bbs5	Obesity
4	Bbs7	Obesity
4	Bdnf	Obesity
4	Cadm1	Obesity
4	Cadm2	Obesity
4	Calcr	Obesity
4	Cbx6	Breast cancer
4	Ccdc170	Breast cancer
4	Doc2a	Obesity
4	Enpp7	Breast cancer
4	Entpd6	Obesity
4	Erbb4	Obesity
4	Esr1	Breast cancer
4	Ets2	Obesity
4	Fhit	Obesity
4	Fto	Obesity
4	Gpr151	Obesity
4	Grid1	Obesity
4	Grp	Obesity
4	Hlf	Breast cancer
4	Ifngr1	Obesity
4	Kcnn4	Breast cancer

Supplementary Table 20 | Cont.

Module #	Gene Name	Disease
4	Kctd13	Obesity
4	Lingo2	Obesity
4	Lrfn2	Obesity
4	Lrp1b	Obesity
4	Lztfl1	Obesity
4	Mapk3	Obesity
4	Mc4r	Obesity
4	Mchr1	Breast cancer
4	Negr1	Obesity
4	Nf1	Breast cancer
4	Nt5c2	Obesity
4	Ntrk2	Obesity
4	Nudt17	Breast cancer
4	Ocel1	Breast cancer
4	Ogfod1	Breast cancer
4	Park2	Obesity
4	Pcsk1	Obesity
4	Plxna4	Obesity
4	Pomc	Obesity
4	Prkd1	Obesity
4	Pten	Breast cancer
4	Qpctl	Obesity
4	Rabep1	Obesity
4	Ralyl	Obesity
4	Rapgef3	Obesity
4	Rarb	Obesity
4	Rasa2	Obesity
4	Rhbdd3	Breast cancer
4	Sema3c	Obesity
4	Sema3d	Obesity
4	Slc22a3	Obesity
4	Ssbp4	Breast cancer
4	Stxbp6	Obesity
4	Taok2	Obesity
4	Tnni3k	Obesity
4	Trim32	Obesity
4	Trim66	Obesity
4	Ttc8	Obesity
4	Tub	Obesity
4	Vkorc1	Obesity
4	Zfr2	Obesity

Supplementary Table 21 | Clustering of obesity-, T1D-, T2D-, autism- and breast cancer-associated genes. Description of each gene including: module number, gene name and disease. Relates to Fig. 13E.

Module #	Gene Name	Disease
1	Abcb11	T2D
1	Adcy3	Obesity/Breast Cancer
1	Adcy5	T2D
1	Agbl4	Obesity
1	Ap2s1	Autism
1	Arhgef9	Autism
1	Arl6	Obesity
1	Atg13	T2D
1	Bbs1	Obesity
1	Bbs5	Obesity
1	Bdnf	Obesity
1	Braf	Autism
1	C2cd4a	T2D
1	C2cd4b	T2D
1	Cacna1c	Autism
1	Cacna1e	Autism
1	Cacna2d3	Autism
1	Cadm1	Obesity
1	Cadm2	Obesity
1	Camk1d	T2D
1	Cbx6	Breast Cancer
1	Cdkl5	Autism
1	Celf4	Autism
1	Chd3	Autism
1	Coro1a	Autism
1	Cry2	T2D
1	Ctla4	T1D
1	Cux2	T1D
1	Dgkb	T2D
1	Dlg4	Autism
1	Dmxl2	Obesity
1	Doc2a	Obesity
1	Erbb4	Obesity
1	Fosl2	T1D
1	Foxp1	Autism
1	Gabrb3	Autism
1	Gnai1	Autism
1	Gria2	Autism
1	Grid1	Obesity

Supplementary Table 21 | Cont.

Module #	Gene Name	Disease
1	Grin2b	Autism
1	Grp	Obesity
1	Hectd4	T2D/Autism
1	Herc2	T1D
1	Hivep2	Autism
1	Hlf	Breast Cancer
1	Hras	Autism
1	Htr1a	T1D
1	Il2	T1D
1	Iqsec2	Autism
1	Jazf1	T2D
1	Kcnb1	Autism
1	Kcnq3	Autism
1	Kctd13	Obesity
1	Kif5a	T1D
1	Lingo2	Obesity
1	Lmo7	T1D
1	Lrfn2	Obesity
1	Lrrc4c	Autism
1	Lztr1	Autism
1	Mapk3	Obesity
1	Mbd5	Autism
1	Mboat7	Autism
1	Mchr1	Breast Cancer
1	Mkx	Autism
1	Myt1l	Autism
1	Nbea	Autism
1	Nckap1	Autism
1	Negr1	Obesity
1	Nlgn2	Autism
1	Nlgn3	Autism
1	Nr3c2	Autism
1	Nr4a2	Autism
1	Nrxn1	Autism
1	Nyap2	T2D
1	Ocel1	Breast Cancer
1	Pacs1	Autism
1	Pax4	T2D
1	Pcsk1	Obesity/T2D
1	Pip4k2c	T1D
1	Plxna4	Obesity

Supplementary Table 21 | Cont.

Module #	Gene Name	Disease
1	Pomgnt1	Autism
1	Ppp1r9b	Autism
1	Pten	Autism/Breast Cancer
1	Rabep1	Obesity
1	Ralyl	Obesity
1	Rarb	Obesity
1	Rasgrp1	T1D/T2D
1	Rims1	Autism
1	Rorb	Autism
1	Scn2a	Autism
1	Sdccag8	Obesity
1	Sec16b	Obesity
1	Sema3c	Obesity
1	Sgsm2	T2D
1	Slc22a3	Obesity
1	Snx7	T2D
1	Srr	T2D
1	Stxbp6	Obesity
1	Syngap1	Autism
1	Tanc2	Autism
1	Tbc1d30	T2D
1	Tbr1	Autism
1	Tnfaip3	T1D
1	Tnni3k	Obesity
1	Trim23	Autism
1	Trim32	Obesity
1	Ube2e2	T2D
1	Wdpcp	Obesity
1	Wfs1	T2D
1	Zfr2	Obesity
2	Abcc8	T2D
2	Ahdc1	Autism
2	Amfr	Breast Cancer
2	Ank1	T2D
2	Ank2	Autism
2	Ankrd11	Autism
2	Appl1	T2D
2	Arap1	T2D
2	Ash1l	Autism
2	Atp6ap1l	Breast Cancer
2	Bbip1	Obesity

Supplementary Table 21 | Cont.

Module #	Gene Name	Disease
2	Bcar1	T2D
2	Bcl2l15	Breast Cancer
2	Brsk2	Autism
2	Cbln1	Obesity
2	Chd7	Autism
2	Cic	Autism
2	Clec16a	T1D
2	Cyp17a1	Obesity
2	D5Ertd579e	Autism
2	Ell	Breast Cancer
2	Epb41l4b	Obesity
2	Fam57b	Obesity
2	Foxo3	Obesity
2	Frrs1l	Obesity
2	Gabrb2	Autism
2	Gcc1	T2D
2	Gckr	T2D
2	Grk5	T2D
2	Hnrnph2	Autism
2	Il27	T1D
2	Inpp5e	Obesity
2	Itgb6	T2D
2	Kcnk3	Obesity
2	Klhl42	T2D
2	Kmt2c	Autism
2	Ksr2	Obesity
2	Lpp	T2D
2	Madd	T2D
2	Map2k5	Obesity
2	Mecp2	Autism
2	Mphosph9	T2D
2	Mtif3	Obesity
2	Ncoa1	Autism
2	Neurod1	T2D
2	Nrxn2	Autism
2	Nrxn3	Obesity/Autism
2	Nudt3	Obesity
2	Pex2	Obesity
2	Pkd1	T1D
2	Plekha1	T1D
2	Poc5	Obesity

Supplementary Table 21 | Cont.

Module #	Gene Name	Disease
2	Pparg	T1D/T2D
2	Ppp2r5d	Autism
2	Ppp5c	Autism
2	Ptchd1	Autism
2	Ptpn22	T1D/Breast Cancer
2	Rasa2	Obesity
2	Reln	Autism
2	Rnf115	Breast Cancer
2	Scg3	Obesity
2	Sema3a	Obesity
2	Sgcg	T2D
2	Sh2b1	Obesity
2	Sh2b3	T1D
2	Shank2	Autism
2	Shank3	Autism
2	Sirt5	Breast Cancer
2	Ski	Autism
2	Smg9	Breast Cancer
2	Stk11	Breast Cancer
2	Sugp1	T2D
2	Suox	T1D
2	Syne1	Breast Cancer
2	Taok1	Autism
2	Tcf4	Autism
2	Tm6sf2	Breast Cancer
2	Tmem163	T2D
2	Tmem184b	Breast Cancer
2	Tnfrsf11b	T1D
2	Trio	Autism
2	Tsc2	Autism
2	Ubr1	Autism
2	Vps13b	Autism
2	Vps13c	T2D
2	Wdfy3	Autism
2	Zbtb38	Breast Cancer
2	Zfp142	Obesity
2	Zfp169	Obesity
2	Zfp703	Breast Cancer
3	Ache	Obesity
3	Adra2a	T2D
3	Agmo	T2D

Supplementary Table 21 | Cont.

Module #	Gene Name	Disease
3	Aldh5a1	Autism
3	Ank3	Autism
3	Ankdd1b	Obesity
3	Ankrd55	T2D
3	Atg10	Breast Cancer
3	Bbs4	Obesity
3	Blk	T2D
3	Ccdc127	Breast Cancer
3	Ccdc91	Breast Cancer
3	Cdk18	T1D
3	Cdkn2b	T2D
3	Cend1	Breast Cancer
3	Cep290	Obesity
3	Cobll1	Obesity/T2D
3	Col4a3bp	Obesity
3	Coq5	Breast Cancer
3	Cox11	Breast Cancer
3	Ctsh	T1D
3	Cyp27a1	Obesity
3	Dffa	Breast Cancer
3	Dip2a	Autism
3	Dnajc27	Obesity/Breast Cancer
3	Efr3b	Obesity/T1D
3	Erap1	T1D
3	Fads1	T2D
3	Faim2	Obesity/T2D
3	Fam189a2	T1D
3	Fhit	Obesity
3	Fitm2	T2D
3	Fto	Obesity/T2D
3	Gbe1	Obesity
3	Gfap	Autism
3	Gnpda2	Obesity
3	Gprc5b	Obesity
3	Grb14	T2D
3	Hapln4	Breast Cancer
3	Hhip	Obesity
3	Hsd17b12	Obesity
3	Iars2	Obesity
3	Ifih1	T1D
3	Ifngr1	Obesity

Supplementary Table 21 | Cont.

Module #	Gene Name	Disease
3	Il2ra	T1D
3	Kank1	T2D
3	Kcnj11	T1D/T2D
3	Kcnk9	Obesity
3	Larp6	T2D
3	Lgr5	T2D
3	Lrp1b	Obesity
3	Map1a	Autism
3	Mc4r	Obesity/T2D
3	Mkks	Obesity
3	Msmo1	T2D
3	Ntrk2	Obesity
3	Nudt17	Breast Cancer
3	Ogfod1	Breast Cancer
3	Park2	Obesity
3	Pcdh9	Obesity
3	Pex14	Breast Cancer
3	Plcd4	Obesity
3	Prkcq	T1D
3	Ptpn11	Autism
3	Ptprd	T2D
3	Rab21	Obesity
3	Rad51d	Breast Cancer
3	Rapgef3	Obesity
3	Ric3	Obesity
3	Rit2	Obesity
3	Scarb2	Obesity
3	Scn1a	Autism
3	Scn8a	Autism
3	Sdha	Breast Cancer
3	Sdhaf4	T2D
3	Sema3b	Obesity
3	Sema3d	Obesity
3	Slc2a2	T2D
3	Slc30a8	T2D
3	Slc6a1	Autism
3	Slc9a6	Autism
3	Ssfa2	Obesity
3	Stx1b	Obesity
3	Stxbp1	Autism
3	Tek	Autism

Supplementary Table 21 | Cont.

Module #	Gene Name	Disease
3	Tgfbr2	Breast Cancer
3	Tlr4	Obesity
3	Tmem160	Obesity
3	Tmem18	Obesity
3	Tns1	Breast Cancer
3	Trim66	Obesity
3	Ypel3	Obesity
3	Zbtb7b	Obesity
4	Ap4b1	Breast Cancer
4	Asb4	Obesity
4	Bbs10	Obesity
4	Bbs2	Obesity
4	Bbs7	Obesity
4	Bbs9	Obesity
4	Calcr	Obesity
4	Ccdc170	Breast Cancer
4	Cdc123	T2D
4	Dda1	Breast Cancer
4	Dpysl2	Autism
4	Dscam	Autism
4	Enpp7	Breast Cancer
4	Entpd6	Obesity
4	Esr1	Breast Cancer
4	Ets2	Obesity
4	Galnt10	Obesity
4	Gck	T2D
4	Gpr151	Obesity
4	Hnf1b	T2D
4	Ifng	T1D
4	Katnal2	Autism
4	Kcnk16	T2D
4	Kcnn4	Breast Cancer
4	Klf14	T2D
4	Lpar3	T1D
4	Lrrc25	Breast Cancer
4	Lztfl1	Obesity
4	Macf1	T2D
4	Maea	T2D
4	Magel2	Autism
4	Nf1	Autism/Breast Cancer
4	Nt5c2	Obesity

Supplementary Table 21 | Cont.

Module #	Gene Name	Disease
4	Ormdl3	T1D
4	Pam	T2D
4	Pcdh19	Autism
4	Pdx1	T2D
4	Phtf1	T1D
4	Pomc	Obesity
4	Prkd1	Obesity
4	Prox1	T2D
4	Qpctl	Obesity
4	Rhbdd3	Breast Cancer
4	Rnaset2a	T1D
4	Rpgrip1l	Obesity
4	Sim1	Obesity
4	Ssbp4	Breast Cancer
4	Stat3	T1D
4	Stk33	Obesity
4	Taf11	T2D
4	Tagap	T1D
4	Taok2	Obesity
4	Tcerg1l	T2D
4	Tm9sf4	Autism
4	Tsc1	Autism
4	Tspan8	T2D
4	Ttc8	Obesity
4	Tub	Obesity
4	Ube3a	Autism
4	Vkorc1	Obesity
4	Zbtb20	Autism
5	Abo	T2D
5	Abraxas1	Breast Cancer
5	Adamts9	T2D
5	Adpgk	Obesity
5	Adsl	Autism
5	Alms1	Obesity
5	Ap3s2	T2D
5	Apobr	Obesity
5	Arl15	T2D
5	Arrdc3	Breast Cancer
5	Ascc2	Breast Cancer
5	Atm	Breast Cancer
5	Atrx	Autism

Supplementary Table 21 | Cont.

Module #	Gene Name	Disease
5	Bard1	Breast Cancer
5	Brca1	Breast Cancer
5	Brca2	Breast Cancer
5	Brip1	Breast Cancer
5	Cars	T1D
5	Ccne1	Breast Cancer
5	Cdca7l	Breast Cancer
5	Cdh1	Breast Cancer
5	Cdkal1	Obesity/T2D
5	Cdkn2a	T2D
5	Cenpo	Breast Cancer
5	Cenpw	T1D
5	Cep295	Obesity
5	Chd8	Autism
5	Chek2	Breast Cancer
5	Cilp2	T2D
5	Cnot3	Autism
5	Cnot9	Obesity
5	Cobl	T1D
5	Ctcf	Autism
5	Cul5	Breast Cancer
5	Dclre1b	Breast Cancer
5	Ddx3x	Autism
5	Dhcr7	Autism
5	Dusp9	T2D
5	Eif3g	Autism
5	Ep300	Autism
5	Epcam	Breast Cancer
5	Erbb3	T1D
5	Etv5	Obesity
5	Faf1	T2D
5	Fancl	Obesity
5	Fmr1	Autism
5	G6pc2	T2D
5	Gatad2a	T2D/Breast Cancer
5	Gdf15	Obesity
5	Gigyf2	Autism
5	Gtf3a	Obesity
5	Hdlbp	Autism
5	Hhex	T2D
5	Hif1an	Obesity

Supplementary Table 21 | Cont.

Module #	Gene Name	Disease
5	Hmga1	Obesity
5	Hmga2	T2D
5	Hnf1a	T2D
5	Hnf4a	T2D
5	Hnrnpu	Autism
5	Hspa4	Breast Cancer
5	Ide	T2D
5	Igf2bp2	T2D
5	Il10	T1D
5	Ino80e	Obesity
5	Itpr3	T1D
5	Kat8	Obesity
5	Kdm3b	Autism
5	Kdm5b	Autism
5	Klf11	T2D
5	Lama1	T2D
5	Lyplal1	T2D
5	Mapk14	T1D
5	Mdm4	Breast Cancer
5	Med13l	Autism
5	Mks1	Obesity
5	Mlh1	Breast Cancer
5	Mrps18c	Breast Cancer
5	Msh2	Breast Cancer
5	Msh6	Breast Cancer
5	Mtch2	Obesity
5	Mtfr2	Obesity
5	Naa15	Autism
5	Nacc1	Autism
5	Nbn	Breast Cancer
5	Nlrc3	Obesity
5	Npat	Breast Cancer
5	Nsd1	Autism
5	Nup155	Autism
5	Nup54	Obesity
5	Oasl1	T2D
5	Palb2	Breast Cancer
5	Pdgfc	T2D
5	Pepd	T2D
5	Pgm1	T1D
5	Phf12	Autism

Supplementary Table 21 | Cont.

Module #	Gene Name	Disease
5	Phf3	Autism
5	Pidd1	Breast Cancer
5	Pms2	Breast Cancer
5	Polk	Obesity
5	Pou5f1	T2D
5	Ppil3	Breast Cancer
5	Prc1	T2D/Breast Cancer
5	Psmd12	Autism
5	Psmd6	T2D
5	Ptk7	Autism
5	Ptpn2	T1D
5	R3hdml	T2D
5	Rab23	Obesity
5	Rad51c	Breast Cancer
5	Ralb	Breast Cancer
5	Rcc1l	Obesity
5	Rpl27a	Obesity
5	Rps23	Breast Cancer
5	Rreb1	T2D
5	Setd5	Autism
5	Sfxn2	Obesity
5	Sin3a	Autism
5	Siva1	Breast Cancer
5	Skap2	T1D
5	Snrpd2	Obesity
5	Spry2	T2D
5	Srpr	Autism
5	Srsf2	T2D
5	Ssr1	T2D
5	Stat4	T1D
5	Tbl1xr1	Autism
5	Tcf20	Autism
5	Tcf7	T2D
5	Tefm	Breast Cancer
5	Thada	T2D
5	Tle4	T2D
5	Tomm40	Obesity
5	Traf7	Autism
5	Trip12	Autism
5	Trp53	Breast Cancer
5	Trp53inp1	T2D

Supplementary Table 21 | Cont.

Module #	Gene Name	Disease
5	Ttll4	Obesity
5	Tyk2	T1D
5	Ubash3a	T1D
5	Usp37	Obesity
5	Vps26a	T2D
5	Wdr43	Breast Cancer
5	Zfp64	Obesity
5	Zfp646	Obesity
6	Adnp	Autism
6	Aff2	Autism
6	Ahrr	Breast Cancer
6	Akap9	Breast Cancer
6	Arid1b	Autism
6	Arx	Autism
6	Asxl3	Autism
6	Atf7ip	Breast Cancer
6	Auts2	Autism
6	Bach2	T1D
6	Baz2b	Autism
6	Bbs12	Obesity
6	Bcdin3d	T2D
6	Bckdk	Autism
6	Bcl11a	T2D/Autism
6	C1qtnf6	T1D
6	Capn10	T2D
6	Cask	Autism
6	Casp8	Breast Cancer
6	Ccnd2	T2D
6	Cd69	T1D
6	Cel	T2D
6	Cfl1	Breast Cancer
6	Champ1	Autism
6	Chd2	Autism
6	Creb1	Obesity
6	Crebbp	Autism
6	Ctnnb1	Autism
6	Cul3	Autism
6	Cyp51	Breast Cancer
6	Deaf1	Autism
6	Dlk1	T1D
6	Dmpk	Autism

Supplementary Table 21 | Cont.

Module #	Gene Name	Disease
6	Dnmt3a	Autism
6	Dpysl5	T2D
6	Dync1h1	Autism
6	Dyrk1a	Autism
6	Ebf3	Autism
6	Efemp2	Breast Cancer
6	Ehbp1	Obesity
6	Ehmt1	Autism
6	Elavl3	Autism
6	Elavl4	Obesity
6	Fbxl19	Obesity
6	Fign	Obesity
6	Foxa2	T2D
6	Foxg1	Autism
6	Foxp2	Autism
6	Frmd3	T1D
6	Gigyf1	Autism
6	Gipr	Obesity/T2D
6	Glis3	T1D/T2D
6	Gpsm1	T2D
6	Grb10	T2D
6	Hip1	Obesity
6	Hmg20a	T2D
6	Hmgcr	Obesity
6	Ift27	Obesity
6	Ift74	Obesity
6	Igf1	T2D
6	Igf2	T2D
6	Ikzf4	T1D
6	Ins2	T1D/T2D
6	Irf2bpl	Autism
6	Irs1	Obesity/T2D
6	Kansl1	Autism
6	Kcnq1	T2D
6	Kctd15	Obesity
6	Kdm6b	Autism
6	Klf7	Obesity
6	Kmt2a	Autism
6	Kmt2e	Autism
6	Kmt5b	Autism
6	L3mbtl3	Breast Cancer

Supplementary Table 21 | Cont.

Module #	Gene Name	Disease
6	Ldb1	Autism
6	Lepr	Obesity
6	Lmo1	Obesity
6	Lmx1b	Obesity
6	Lrrd1	Breast Cancer
6	Med13	Autism
6	Meis2	Autism
6	Nav1	Obesity
6	Nipbl	Autism
6	Nol12	Breast Cancer
6	Notch2	T2D
6	Nrp1	Obesity
6	Nrp2	Obesity
6	Olig3	Obesity
6	Pax5	Autism
6	Pbrm1	Obesity
6	Pgpep1	Obesity/Breast Cancer
6	Phf2	Autism
6	Phf21a	Autism
6	Phip	Obesity/Autism
6	Pik3c2b	Breast Cancer
6	Plxna1	Obesity
6	Plxna2	Obesity
6	Plxna3	Obesity
6	Pogz	Autism
6	Ppil2	T1D
6	Ppip5k2	T2D
6	Ppp1r3b	T2D
6	Prkd2	T1D
6	Prr12	Autism
6	Ptbp2	Obesity
6	Rai1	Autism
6	Rbms1	T2D
6	Rccd1	Breast Cancer
6	Rere	Autism
6	Rfx3	Autism
6	Rnd3	T2D
6	Rnls	T1D
6	Satb1	Autism
6	Sbk1	Obesity
6	Setbp1	Autism

Supplementary Table 21 | Cont.

Module #	Gene Name	Disease
6	Setd2	Autism
6	Skor1	Obesity
6	Slc39a8	Obesity
6	Smarcc2	Autism
6	Smg6	Obesity
6	Sox5	Autism
6	Spast	Autism
6	Srcap	Autism
6	St6gal1	T2D
6	Stk36	Obesity
6	Tal1	Obesity
6	Tbck	Autism
6	Tcf19	T2D
6	Tcf7l2	Obesity/T1D/T2D/Autism
6	Tfap2b	Obesity
6	Tle1	T2D
6	Tlk2	Autism
6	Tlr1	Breast Cancer
6	Tnnt3	Breast Cancer
6	Tshz3	Autism
6	Txndc12	Obesity
6	Ube2e3	Obesity
6	Upf3b	Autism
6	Vezf1	Autism
6	Wac	Autism
6	Zbed3	T2D
6	Zbtb10	Obesity
6	Zfand3	T2D
6	Zfand6	T2D
6	Zfhx3	Obesity
6	Zfp292	Autism
6	Zfp462	Autism
6	Zfp608	Obesity
6	Zfp668	Obesity
6	Zmiz1	T2D
6	Zmynd8	Autism
